# Eco-Friendly and Efficient Synthesis of 2-Hydroxy-3-Hydrazono-Chromones Through *α*,*β*-C(sp^2^)–H Bond Difunctionalization/Chromone Annulation Reaction of *o*-Hydroxyaryl Enaminones, Water, and Aryldiazonium Salts

**DOI:** 10.3390/molecules30061194

**Published:** 2025-03-07

**Authors:** Xiaohong Wang, Menglin Peng, Yijin Wang, Siyu Song, Ying Xu, Li Chen, Fuchao Yu

**Affiliations:** Faculty of Life Science and Technology, Kunming University of Science and Technology, Kunming 650500, China; wangxiaohong@stu.kust.edu.cn (X.W.); 15680220116@163.com (M.P.); 13987717845@139.com (Y.W.); songsiyu083@163.com (S.S.); 13609171778@163.com (Y.X.)

**Keywords:** 2,3-disubstituted chromones, *o*-hydroxyaryl enaminones, aryldiazonium salts, eco friendly, chromone annulation reaction

## Abstract

A novel, eco-friendly, and efficient method for constructing 2,3-disubstituted chromone skeletons from readily available water, *o*-hydroxyaryl enaminones (*o*-HPEs), and aryldiazonium salts has been developed under mild reaction conditions. This *α*,*β*-C(sp^2^)–H bond difunctionalization/chromone annulation reaction strategy is achieved by building two C(sp^3^)–O bonds and a C(sp^2^)-N bond, which provides a practical pathway for the preparation of 2-hydroxy-3-hydrazono-chromones in moderate to excellent yields, enabling broad substrate scope and good functional group tolerance, as well as gram-scale synthesis. This protocol offers a valuable tool for synthesizing diverse functionalized chromones with potential applications in drug discovery and industrial synthesis.

## 1. Introduction

**Figure 1:**
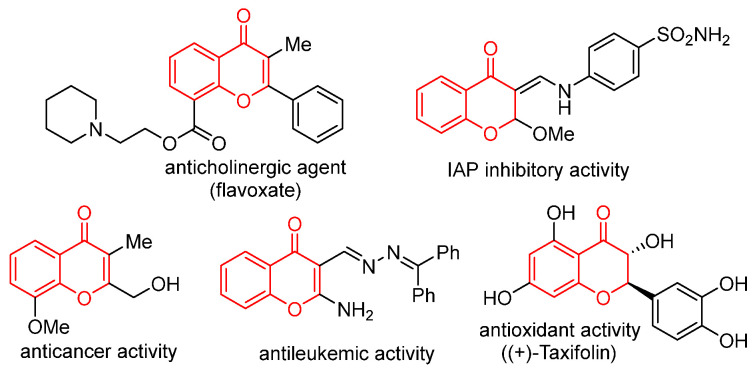
Selected biologically active 2,3-disubstituted chromones.

2,3-Disubstituted chromones are an important class of *O*-heterocyclic compounds that are widely distributed in the skeleton of natural products and drug molecules [1,2,3,4,5]. In the field of medicinal chemistry, chromones exhibit a wide range of pharmacological properties [6,7,8,9,10], including anti-inflammatory, antioxidant, anticholinergic, and anticancer activities, making them valuable lead compounds for drug discovery (Figure 1). Consequently, numerous efforts for developing novel and efficient strategies to these 2,3-disubstituted chromone molecules have been developed. In recent decades, conventional synthetic approaches toward 2,3-disubstituted chromones synthesis primarily involve the following chromone annulation reactions: (1) starting from 3-substituted chromones through a four-component reaction or transition metal (e.g., Pd)-catalyzed functionalization reactions [11,12]; (2) cyclization reactions such as the Kostanecki–Robinson reaction and Baker–Venkataraman rearrangement [13,14]; (3) two-step electrophilic cyclization or Suzuki cross-coupling reaction using 1-(2-methoxyphenyl)but-2-yn-1-one as the starting material [15]; (4) transition metal-catalyzed or electro-catalyzed coupling reactions of salicylaldehydes with alkynes [16]; (5) Claisen condensation of *o*-hydroxyphenylacetone derivatives with esters[17]; and (6) Pd-catalyzed S_N_Ar reactions of 2-fluorobromobenzenes with ketones [18]. Despite the development of numerous synthetic methods for 2,3-disubstituted chromones, several challenges remain to be addressed, including the requirement for specialized and pre-functionalized substrates, the need for transition metal catalysts, and harsh reaction conditions. Consequently, there is an urgent need to develop new, green, and efficient methods for constructing such compounds.

**Scheme 1:**
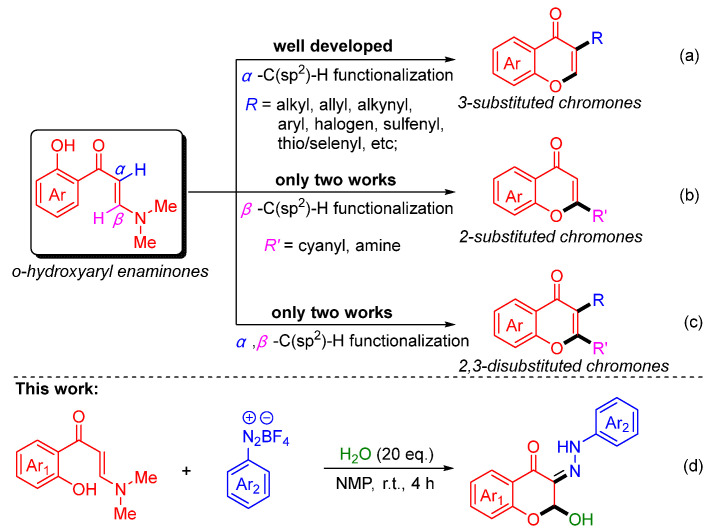
(**a**) The construction methods of 3-substituted chromones from *o*-HPEs; (**b**) The construction methods of 2-substituted chromones from *o*-HPEs; (**c**) The construction methods of 2,3-disubstituted chromones from *o*-HPEs; (**d**) This work: the construction methods of 2-hydroxy-3-hydrazono-chromones from *o*-HPEs.

*o*-Hydroxyaryl enaminones (*o*-HPEs), as a structurally unique class of enaminone compounds, are widely used in the synthesis of chromone derivatives due to their ease of preparation and high reactivity [19,20]. In this context, the distinctive conjugated structure of o-HPEs makes their vinyl α-position highly susceptible to attack by various electrophiles for the simple and efficient construction of 3-substituted chromones through cascade vinyl *α*-C(sp^2^)–H bond functionalization/intramolecular chromone annulation process (Scheme 1a). Various vinyl *α*-C(sp^2^)–H bond functionalizations include trifluoromethylation [21,22,23], alkylation [24,25,26,27], allylation [28,29], alkenylation [30], arylation [31,32,33], acylation [34,35], amination [36,37], halogenation [38,39], thiolation [40,41,42], selenylation [43,44], and phosphorylation [45], acyloxylation [46], sulfonylation [47,48], etc. In contrast, the vinyl *β*-position of *o*-HPEs is relatively inert but can be activated under specific conditions to construct 2-substituted chromone frameworks through the selective cascade vinyl *β*-C(sp^2^)–H bond functionalization/intramolecular chromone annulation process (Scheme 1b). In 2020, Wan’s group developed a copper-catalyzed tandem cyclization reaction of *o*-HPEs with secondary amines for the construction of 2-nitrogenated chromones [49]. In 2023, they further discovered that *o*-HPEs could undergo *β*-cyanation in the presence of potassium ferricyanide and iodine, enabling the introduction of cyanyl at the 2-position of chromones [50]. Nevertheless, these forementioned *α*- or *β*-C(sp^2^)–H bond functionalization/intramolecular chromone annulation reactions of *o*-HPEs only form a new bond at the 2- or 3-position of vinyl on the newly formed chromone skeleton to construct 2-substituted or 3-substituted chromones. On the other hand, regarding the *α*,*β*-C(sp^2^)–H bond difunctionalization/chromone annulation reaction of *o*-HPEs, it has recently been disclosed that two new bonds simultaneously form at the 2- and 3-position of vinyl on the newly formed chromone skeleton to construct 2,3-disubstituted chromones (Scheme 1c). At this stage, such difunctionalization/chromone annulation reactions are scarce and remain underdeveloped. Recently, Yang’s group reported the first example of a difunctionalization reaction at both the *α*- and *β*-positions of *o*-HPEs using tert-butyl nitrite as an electrophile and water as a nucleophile, leading to the synthesis of 2,3-disubstituted chromones [51]. Subsequently, in 2024, our group developed a similar difunctionalization strategy using aryldiazonium salts as electrophiles and alcohols as nucleophiles [52]. As a part of our ongoing interest in chromone annulation reactions [24,25,52] and enaminones chemistry [53,54,55,56], we report herein a novel *α*,*β*-C(sp^2^)–H bond difunctionalization/chromone annulation reaction of *o*-HPEs, aryldiazonium salts, and water (Scheme 1d). Notably, the synthesis approach is greener and milder to efficiently yield structurally diverse 2-hydroxy-3-hydrazono-chromones.

## 2. Results and Discussion

We commenced this study by exploring this cascade *α*,*β*-C(sp^2^)–H bond difunctionalization/chromone annulation reaction, and *o*-hydroxyarylenaminone **1a** and *p*-methoxy diazonium salt **2a** were chosen as model substrates to evaluate the reaction condition (Table 1). Initially, the reaction was carried out in THF with water (20 equiv.) as an additive, stirring at room temperature in air for 4 h, yielding the target 2-hydroxy-3-hydrazono-chromone **3a** in 65% yield. To further improve the reaction yield, various solvents were screened, including 2-MeTHF, 1,4-dioxane, 1,3-dioxane, EA, CHCl_3_, DCE, toluene, chlorobenzene, DMF, DMA, and NMP (entries 2–12). The results showed that **3a** was not formed in CHCl_3_, toluene, or chlorobenzene, while the highest yield was 88% in NMP. Subsequently, the amount of water was optimized. Without additional water, the yield was only 40% (entry 13), and by increasing or decreasing the amount of water, the yield was slightly lower (entries 14 and 15). The equivalent of diazonium salt **2a** was also screened, with 1.5 equivalents found to be optimal (entries 16 and 17). Additionally, the reaction temperature, atmosphere, and time were also investigated (entries 18–23). The optimal reaction condition was determined as follows: **1a** (0.1 mmol), **2a** (0.15 mmol), and water (20 equiv.) in NMP at room temperature under air for 4 h.

**Scheme 2:**
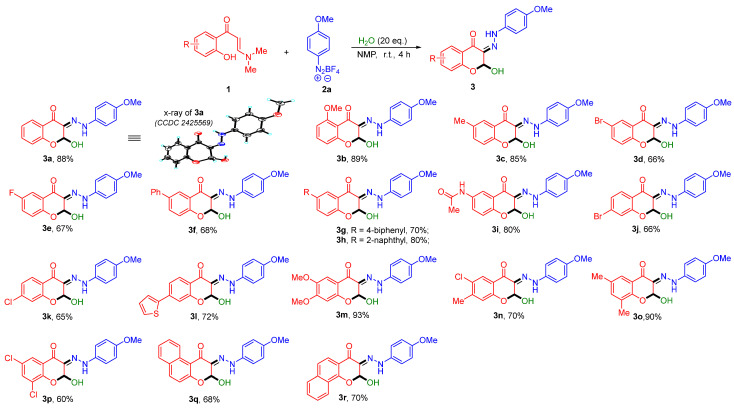
Scope of *o*-hydroxyaryl enaminones *^a,b^*. *^a^* Reaction conditions: **1** (0.3 mmol), **2** (0.45 mmol), and H_2_O (20 equiv.) in 3 mL of NMP, stirred at room temperature for 4.0 h. *^b^* Isolated yields.

Under the optimized reaction conditions, we initially investigated the substrate scope of *o*-HPEs with aryldiazonium salt **2a** (Scheme 2). The results demonstrated excellent substrate compatibility of *o*-HPEs to afford the corresponding 2-hydroxy-3-hydrazono-chromones **3a**–**3p** in 60–93% yields, which exhibited broad tolerance to both electron-donating groups (OMe, Me) and electron-withdrawing groups (Br, F, phenyl, biphenyl, naphthyl, amide, and thiophene) on the aromatic ring of *o*-HPEs. Generally, *o*-HPEs with substituents at the 6-position (**3b**), 5-position (**3c**–**3i**), or 4-position (**3j**–**3l**) all afforded the target products in moderate to excellent yields. Furthermore, *o*-HPEs with multiple substituents also performed well in this transformation (**3m**–**3p**). In addition, 1-naphthyl and 2-naphthyl replacement of the benzene ring of *o*-HPEs were further evaluated. The reaction also proceeded smoothly, providing the corresponding target compounds **3q**–**3r** in 68–70% yields. The structure of **3a** was unequivocally confirmed by X-ray crystallographic analysis (CCDC 2425569).

**Scheme 3:**
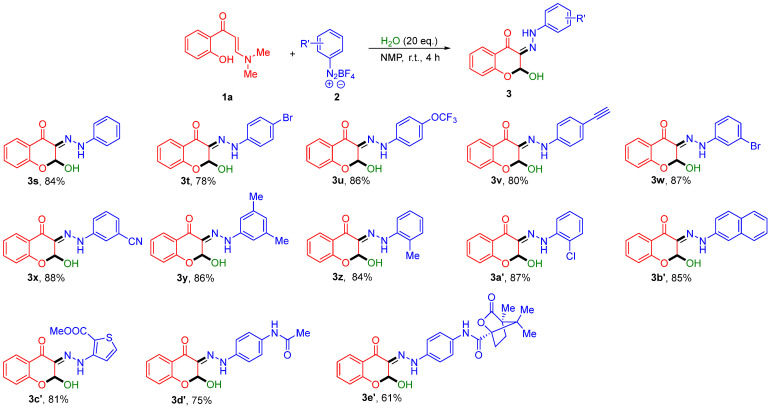
Scope of aryldiazonium salts *^a,b^*. *^a^* Reaction conditions: **1** (0.3 mmol), **2** (0.45 mmol), and H_2_O (20 equiv.) in 3 mL of NMP, stirred at room temperature for 4.0 h. *^b^* Isolated yields.

Subsequently, the scope of aryldiazonium salts **2** was also explored in the reaction using *o*-HPEs **1a** as a partner (Scheme 3). For various aryl diazonium salts bearing halogen (Cl and Br), OCF_3_, alkynyl, cyano, and methyl substituents at *ortho*, *meta*, or *para*-positions, the desired 2-hydroxy-3-hydrazono-chromone products (**3s**–**3a′**) could be furnished in excellent yields (78–88%). Moreover, 2-naphthyl- and 3-thienyl-substituted components were also compatible with this transformation, providing the corresponding compounds **3b′**–**3c′** in 81–85% yields. Notably, even diazonium salts bearing amide substituents on the benzene ring were well-tolerated, especially (−)-camphanic acid-derived diazonium salt, affording compounds **3d′**–**3e′** in 61–75% yields. This remarkable performance with amide-containing substrates further highlights the excellent substrate generality of this transformation, which can serve as a valuable tool for the synthesis of various functionalized compounds.

**Scheme 4:**
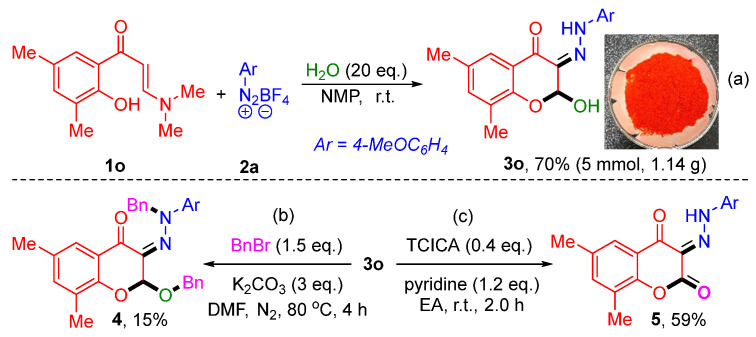
(**a**) Gram-scale synthesis; (**b**) Benzylation derivatization; (**c**) Oxidation derivatization.

To further demonstrate the potential practicability of this method, a gram-scale synthesis and several further transformations were implemented (Scheme 4). First, a 5.0 mmol scale reaction was carried out under the standard reaction conditions to afford the desired product **3o** in 70% (1.14 g) (Scheme 4a). Next, some further transformations of 2-hydroxy-3-hydrazono-chromones **3** were performed by benzylation and oxidation reactions. A bibenzylated product **4** in 15% yield was prepared by the reaction of **3o** with benzyl bromide in the presence of K_2_CO_3_ (Scheme 4b). A chromane-2,4-dione derivative **5** with 59% yield could be synthetized by the treatment of **3o** with trichloroisocyanuric acid (TCICA) and pyridine in ethyl acetate (EA) at room temperature (Scheme 4c).

**Scheme 5:**
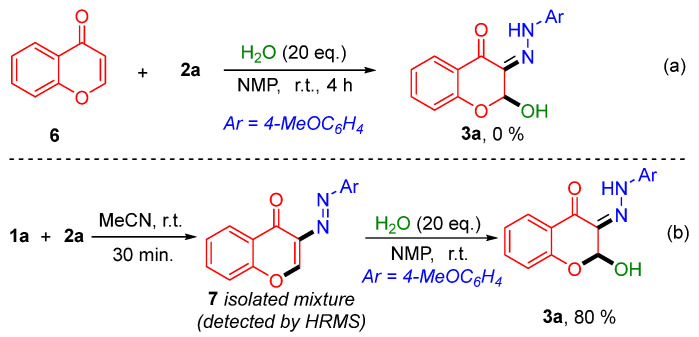
(**a**) Intermediate validation experiments; (**b**) Intermediate-capture experiments.

To gain a deeper understanding of the reaction mechanism, a series of control experiments were conducted. Initially, the reaction of chromone **6** and *p*-methoxy diazonium salt **2a** was carried out under the standard reaction conditions, and the target product **3a** was not observed (Scheme 5a). Subsequently, *o*-HPE **1a** and *p*-methoxy diazonium salt **2a** were reacted in acetonitrile at room temperature for 30 min, leading to the formation of an unstable intermediate **7**, and then treated with water (20 equiv.) under the standard reaction conditions, resulting in the target compound **3a** in 80% yield (Scheme 5b). These results confirmed that the formation of **3a** was achieved through the initial coupling of the diazonium salt with the *o*-HPE, followed by cyclization to form the chromone skeleton.

**Scheme 6:**
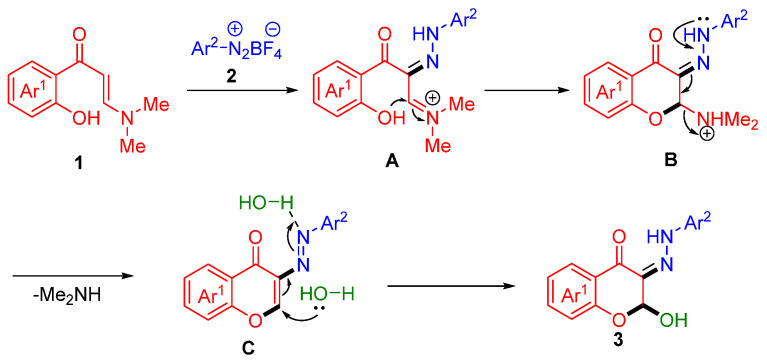
Proposed mechanism.

Based on the above experimental results and previous reports [51,52], a plausible mechanism for this cascade *α*,*β*-C(sp^2^)–H bond difunctionalization/chromone annulation reaction was proposed in Scheme 6. First, diazonium salts **2** are coupled with *α*-position of *o*-HPEs **1** to form intermediate A, then undergo intramolecular cyclization and deamination to yield intermediate C, which possesses the chromone skeleton. The nitrogen atom of intermediate C forms an intermolecular hydrogen bond with water, activating the N=N double bond. Finally, another molecule of water attacks the 2-position of the chromone skeleton to form the target compound **3**.

## 3. Materials and Methods

### 3.1. General Information

All the compounds were fully characterized by spectroscopic data. The NMR spectra were recorded on a DRX600 (^1^H: 600 MHz, ^13^C: 150 MHz) and an AV500 (^1^H: 500 MHz, ^13^C: 125 MHz), chemical shifts (*δ*) are expressed in ppm, *J* values are given in Hz, and deuterated DMSO-*d*_6_ and Pyridine-*d*_6_ were used as solvents. The reactions were monitored by thin-layer chromatography (TLC) using silica gel GF_254_. The melting points were determined on an XT-4A melting point apparatus and are uncorrected. HRMs were performed on an Agilent LC/MS TOF instrument (From Agilent Technologies Inc., Santa Clara, CA, USA).

All the chemicals and solvents were used as received without further purification, unless otherwise stated. Column chromatography was performed on silica gel (200–300 mesh).

*o*-Hydroxyphenylenaminones **1** were prepared according to the literature [57,58,59], and aryldiazonium salts **2** were prepared according to the literature [60,61]. Other reagents were purchased from Energy Chemical and Adamas-beta^®^ (From Shanghai, China).

### 3.2. Synthetic Procedures

#### 3.2.1. Synthetic Procedures of 2-Hydroxy-3-Hydrazono-Chromones 3

*o*-Hydroxyarylenaminones **1** (0.3 mmol), aryldiazonium salts **2** (0.45 mmol), H_2_O (20 equiv.), and NMP (3.0 mL) were charged into 10 mL Ace Glass pressure tubes, and the mixture was stirred at r.t. for 4.0 h until *o*-hydroxyarylenaminones were completely consumed. EtOAc (50 mL × 2) were added. The organic phase was washed with water (30 mL), dried over Na_2_SO_4_, concentrated, and purified by flash column chromatography to afford 2-hydroxy-3-hydrazono-chromones **3**.

#### 3.2.2. Synthetic Procedures of Bibenzylated 2-Hydroxy-3-Hydrazono-Chromone 4

2-Hydroxy-3-hydrazono-chromone **3o** (0.3 mmol), benzyl bromide (0.45 mmol), potassium carbonate (3.0 equiv.), and DMF (5.0 mL) were charged into 10 mL Ace Glass pressure tubes, and the mixture was stirred at 80 °C for 4.0 h until **3o** was completely consumed. EtOAc (5 mL × 2) were added. The organic phase was washed with water (10 mL), dried over Na_2_SO_4_, concentrated, and purified by flash column chromatography to afford bibenzylated 2-hydroxy-3-hydrazono-chromone **4**.

#### 3.2.3. Synthetic Procedures of Chromane-2,4-Dione 5

2-Hydroxy-3-hydrazono-chromone **3o** (0.3 mmol), trichloroisocyanuric acid (0.4 equiv.), pyridine (1.2 equiv.), and ethyl acetate (3.0 mL) were charged into 10 mL Ace Glass pressure tubes, and the mixture was stirred at r.t. for 2.0 h until **3o** was completely consumed. EtOAc (5 mL × 2) were added. The organic phase was washed with water (10 mL), dried over Na_2_SO_4_, concentrated, and purified by flash column chromatography to afford chromane-2,4-dione **5**.

### 3.3. Characterization of Products

**2-Hydroxy-3-(2-(4-methoxyphenyl)hydrazineylidene)chroman-4-one (3a).** V_Petroleum ether_/V_Ethyl acetate_ = 10:1, R*_f_* = 0.2; Red solid: 79 mg (88%); mp = 154–155 °C; ^1^H NMR (600 MHz, DMSO-*d*_6_): *δ* = 13.99 (s, 1H, OH), 7.88 (d, *J* = 7.4 Hz, 1H, NH), 7.77 (d, *J* = 5.5 Hz, 1H, ArH), 7.62 (t, *J* = 7.2 Hz, 1H, ArH), 7.43 (d, *J* = 8.5 Hz, 2H, ArH), 7.19–7.16 (m, 1H, ArH), 7.10 (d, *J* = 8.1 Hz, 1H, ArH), 6.98 (d, *J* = 8.6 Hz, 2H, ArH), 6.09 (d, *J* = 5.5 Hz, 1H, O-CH), 3.76 (s, 3H, ArOCH_3_); ^13^C NMR (150 MHz, DMSO-*d*_6_): *δ* = 177.0, 156.8, 156.8, 136.5, 136.1, 129.3, 126.9, 122.6, 122.3, 119.2, 117.0, 117.0, 115.3, 115.3, 95.9, 55.8; HRMS (TOF ES+): *m*/*z* calcd for C_16_H_14_N_2_NaO_4_^+^ [(M+Na)^+^], 321.0846, found, 321.0858.

**2-Hydroxy-5-methoxy-3-(2-(4-methoxyphenyl)hydrazineylidene)chroman-4-one (3b).** V_Petroleum ether_/V_Ethyl acetate_ = 5:1, R*_f_* = 0.2; Red solid: 88 mg (89%); mp = 199–200 °C; ^1^H NMR (600 MHz, DMSO-*d*_6_): *δ* = 14.09 (s, 1H, OH), 7.67 (d, *J* = 5.7 Hz, 1H, NH), 7.50 (t, *J* = 8.3 Hz, 1H, ArH), 7.39 (d, *J* = 8.9 Hz, 2H, ArH), 6.95 (d, *J* = 8.9 Hz, 2H, ArH), 6.76 (d, *J* = 8.4 Hz, 1H, ArH), 6.64 (d, *J* = 8.2 Hz, 1H, ArH), 5.95 (d, *J* = 5.7 Hz, 1H, O-CH), 3.85 (s, 3H, ArOCH_3_), 3.74 (s, 3H, ArOCH_3_); ^13^C NMR (150 MHz, DMSO-*d*_6_): *δ* = 176.8, 160.9, 157.9, 156.0, 136.2, 136.1, 129.2, 116.2, 116.2, 114.8, 114.8, 112.1, 110.8, 105.5, 95.4, 56.0, 55.4; HRMS (TOF ES+): *m*/*z* calcd for C_17_H_16_N_2_NaO_5_^+^ [(M+Na)^+^], 351.0951, found, 351.0960.

**2-Hydroxy-3-(2-(4-methoxyphenyl)hydrazineylidene)-6-methylchroman-4-one (3c).** V_Petroleum ether_/V_Ethyl acetate_ = 10:1, R*_f_* = 0.2; Red solid: 80 mg (85%); mp = 164–165 °C; ^1^H NMR (600 MHz, DMSO-*d*_6_): *δ* = 13.97 (s, 1H, OH), 7.69 (d, *J* = 5.7 Hz, 1H, NH), 7.64 (s, 1H, ArH), 7.42–7.40 (m, 3H, ArH), 7.00–6.96 (m, 3H, ArH), 6.04 (d, *J* = 5.6 Hz, 1H, O-CH), 3.75 (s, 3H, ArOCH_3_), 2.31 (s, 3H, ArCH_3_); ^13^C NMR (150 MHz, DMSO-*d*_6_): *δ* = 176.8, 156.3, 154.4, 137.0, 135.7, 131.2, 129.1, 126.0, 121.6, 118.6, 116.5, 116.5, 114.9, 114.9, 95.4, 55.4, 20.2; HRMS (TOF ES+): *m*/*z* calcd for C_17_H_16_N_2_NaO_4_^+^ [(M+Na)^+^], 335.1002, found, 335.1006.

**6-Bromo-2-hydroxy-3-(2-(4-methoxyphenyl)hydrazineylidene)chroman-4-one (3d).** V_Petroleum ether_/V_Ethyl acetate_ = 10:1, R*_f_* = 0.2; Red solid: 75 mg (66%); mp = 180–181 °C; ^1^H NMR (600 MHz, DMSO-*d*_6_): *δ* = 13.95 (s, 1H, OH), 7.91–7.89 (m, 1H, ArH), 7.88 (d, *J* = 5.9 Hz, 1H, NH), 7.76–7.73 (m, 1H, ArH), 7.45–7.42 (m, 2H, ArH), 7.10–7.08 (m, 1H, ArH), 6.97 (d, *J* = 9.3 Hz, 2H, ArH), 6.10 (d, *J* = 5.6 Hz, 1H, O-CH), 3.75 (s, 3H, ArOCH_3_); ^13^C NMR (150 MHz, DMSO-*d*_6_): *δ* = 174.9, 156.7, 155.5, 138.3, 135.5, 128.4, 128.3, 123.6, 121.4, 116.9, 116.9, 114.9, 114.9, 113.9, 95.7, 55.4; HRMS (TOF ES+): *m*/*z* calcd for C_16_H_13_BrN_2_NaO_4_^+^ [(M+Na)^+^], 398.9951, found, 398.9987.

**6-Fluoro-2-hydroxy-3-(2-(4-methoxyphenyl)hydrazineylidene)chroman-4-one (3e).** V_Petroleum ether_/V_Ethyl acetate_ = 10:1, R*_f_* = 0.2; Red solid: 64 mg (68%); mp = 160–161 °C; ^1^H NMR (600 MHz, DMSO-*d*_6_): *δ* = 13.97 (s, 1H, OH), 7.80 (d, *J* = 5.9 Hz, 1H, NH), 7.56–7.54 (m, 1H, ArH), 7.51–7.47 (m, 1H, ArH), 7.44 (d, *J* = 8.6 Hz, 2H, ArH), 7.17–7.15 (m, 1H, ArH), 6.98 (d, *J* = 8.9 Hz, 2H, ArH), 6.08 (d, *J* = 5.8 Hz, 1H, O-CH), 3.75 (s, 3H, ArOCH_3_); ^13^C NMR (150 MHz, DMSO-*d*_6_): *δ* = 175.4, 157.1 (C–F, *J* = 238.5 Hz), 156.6, 152.7, 135.6, 128.5, 123.3 (C–F, *J* = 24.4 Hz), 122.6 (C–F, *J* = 6.5 Hz), 120.8 (C–F, *J* = 7.9 Hz), 116.8, 116.8, 114.9, 114.9, 111.3 (C–F, *J* = 23.6 Hz), 95.5, 55.4; ^19^F NMR (470 MHz, DMSO-*d*_6_) *δ* = -120.9; HRMS (TOF ES+): *m*/*z* calcd for C_16_H_13_FN_2_NaO_4_^+^ [(M+Na)^+^], 339.0752, found, 339.0756.

**2-Hydroxy-3-(2-(4-methoxyphenyl)hydrazineylidene)-6-phenylchroman-4-one (3f).** V_Petroleum ether_/V_Ethyl acetate_ = 10:1, R*_f_* = 0.1; Red solid: 76 mg (68%); mp = 185–186 °C; ^1^H NMR (500 MHz, DMSO-*d*_6_): *δ* = 14.04 (s, 1H, OH), 8.07 (d, *J* = 2.4 Hz, 1H,ArH), 7.92–7.90 (m, 1H, ArH), 7.82 (d, *J* = 5.8 Hz, 1H, NH), 7.67 (d, *J* = 9.0 Hz, 2H, ArH), 7.50–7.47 (m, 2H, ArH), 7.43 (d, *J* = 9.0 Hz, 2H, ArH), 7.39–7.36 (m, 1H, ArH), 7.20 (d, *J* = 8.5 Hz, 1H, ArH), 6.99–6.97 (m, 2H, ArH), 6.13 (d, *J* = 5.8 Hz, 1H, O-CH), 3.75 (s, 3H, ArOCH_3_); ^13^C NMR (125 MHz, DMSO-*d*_6_): *δ* = 176.4, 156.4, 155.9, 138.9, 135.6, 134.4, 134.2, 129.1, 129.1, 128.9, 127.5, 126.4, 126.4, 123.9, 122.1, 119.4, 116.6, 116.6, 114.9, 114.9, 95.6, 55.4; HRMS (TOF ES+): *m*/*z* calcd for C_22_H_18_N_2_NaO_4_^+^ [(M+Na)^+^], 397.1159, found, 397.1156.

**6-([1,1′-Biphenyl]-4-yl)-2-hydroxy-3-(2-(4-methoxyphenyl)hydrazineylidene)chroman-4-one (3g).** V_Petroleum ether_/V_Ethyl acetate_ = 10:1, R*_f_* = 0.1; Red solid: 95 mg (70%); mp = 225–226 °C; ^1^H NMR (500 MHz, Pyridine-*d*_5_): *δ* = 14.65 (s, 1H, OH), 8.56–8.55 (m, 1H, ArH), 7.95–7.92 (m, 1H, NH), 7.79–7.74 (m, 7H, ArH), 7.53–7.48 (m, 4H, ArH), 7.44–7.39 (m, 2H, ArH), 7.07–7.04 (m, 2H, ArH), 6.83 (s, 1H, O-CH), 3.69 (s, 3H, ArOCH_3_); ^13^C NMR (125 MHz, Pyridine-*d*_5_): *δ* = 178.4, 157.7, 157.7, 141.3, 140.7, 139.5, 137.0, 135.2, 135.0, 130.9, 129.9, 129.9, 128.4, 128.4, 128.3, 128.0, 128.0, 127.8, 127.8, 125.6, 120.5, 117.4, 117.4, 115.7, 115.7, 97.7, 55.9, 55.9; HRMS (TOF ES+): *m*/*z* calcd for C_28_H_22_N_2_NaO_4_^+^ [(M+Na)^+^], 473.1472, found, 473.1469.

**2-Hydroxy-3-(2-(4-methoxyphenyl)hydrazineylidene)-6-(naphthalen-2-yl)chroman-4-one (3h).** V_Petroleum ether_/V_Ethyl acetate_ = 10:1, R*_f_* = 0.1; Red solid: 102 mg (80%); mp = 187–188 °C; ^1^H NMR (600 MHz, DMSO-*d*_6_): *δ* = 14.07 (s, 1H, OH), 8.22 (s, 2H, ArH), 8.07–8.05 (m, 1H, ArH), 8.02 (d, *J* = 8.3 Hz, 2H, ArH), 7.94 (d, *J* = 7.8 Hz, 1H, NH), 7.87 (d, *J* = 5.9 Hz, 1H, ArH), 7.84 (d, *J* = 10.4 Hz, 1H, ArH), 7.55–7.52 (m, 2H, ArH), 7.43 (d, *J* = 6.7 Hz, 2H, ArH), 7.24 (d, *J* = 8.5 Hz, 1H, ArH), 6.98 (d, *J* = 8.9 Hz, 2H, ArH), 6.14 (d, *J* = 5.8 Hz, 1H, O-CH), 3.75 (s, 3H, ArOCH_3_); ^13^C NMR (150 MHz, DMSO-*d*_6_): *δ* = 176.4, 156.5, 156.1, 136.3, 135.7, 134.7, 134.0, 133.4, 132.3, 128.9, 128.8, 128.3, 127.6, 126.6, 126.3, 125.0, 124.8, 124.2, 122.2, 119.6, 116.6, 116.6, 114.9, 114.9, 95.7, 55.4; HRMS (TOF ES+): *m*/*z* calcd for C_26_H_20_N_2_NaO_4_^+^ [(M+Na)^+^], 447.1315, found, 447.1313.

***N*-(2-Hydroxy-3-(2-(4-methoxyphenyl)hydrazineylidene)-4-oxochroman-6-yl)acetamide (3i).** V_Petroleum ether_/V_Ethyl acetate_ = 3:1, R*_f_* = 0.2; Red solid: 85 mg (80%); mp > 250 °C; ^1^H NMR (600 MHz, DMSO-*d*_6_): *δ* = 13.97 (s, 1H, OH), 10.08 (s, 1H, ArNH), 8.14 (d, *J* = 2.7 Hz, 1H, N=NH), 7.74–7.72 (m, 1H, ArH), 7.70 (d, *J* = 5.9 Hz, 1H, ArH), 7.42 (d, *J* = 8.5 Hz, 2H, ArH), 7.05 (d, *J* = 8.8, 1H, ArH), 6.97 (d, *J* = 8.6 Hz, 2H, ArH), 6.04 (d, *J* = 5.8 Hz, 1H, O-CH), 3.75 (s, 3H, ArOCH_3_), 2.05 (s, 3H, C-CH_3_); ^13^C NMR (150 MHz, DMSO-*d*_6_): *δ* = 176.5, 168.3, 156.4, 152.1, 135.7, 133.9, 129.0, 127.4, 121.7, 119.0, 116.6, 116.6, 115.9, 115.9, 114.9, 95.4, 55.4, 23.9; HRMS (TOF ES+): *m*/*z* calcd for C_18_H_17_N_3_NaO_5_^+^ [(M+Na)^+^], 378.1060, found, 378.1064.

**7-Bromo-2-hydroxy-3-(2-(4-methoxyphenyl)hydrazineylidene)chroman-4-one (3j).** V_Petroleum ether_/V_Ethyl acetate_ = 10:1, R*_f_* = 0.2; Red solid: 75 mg (66%); mp = 190–191 °C; ^1^H NMR (600 MHz, DMSO-*d*_6_): *δ* = 13.96 (s, 1H, OH), 7.91 (d, *J* = 5.9 Hz, 1H, NH), 7.78 (d, *J* = 8.2 Hz, 1H, ArH), 7.43–7.42 (m, 2H, ArH), 7.38–7.35 (m, 2H, ArH), 6.97 (d, *J* = 9.1 Hz, 2H, ArH), 6.11 (d, *J* = 5.9 Hz, 1H, O-CH), 3.75 (s, 3H, ArOCH_3_); ^13^C NMR (150 MHz, DMSO-*d*_6_): *δ* = 175.6, 156.8, 156.5, 135.6, 129.2, 128.5, 128.2, 125.5, 121.5, 121.2, 116.7,116.7, 114.9, 114.9, 96.1, 55.4; HRMS (TOF ES+): *m*/*z* calcd for C_16_H_13_BrN_2_NaO_4_^+^ [(M+Na)^+^], 398.9951, found, 398.9944.

**7-Chloro-2-hydroxy-3-(2-(4-methoxyphenyl)hydrazineylidene)chroman-4-one (3k).** V_Petroleum ether_/V_Ethyl acetate_ = 10:1, R*_f_* = 0.2; Red solid: 65 mg (65%); mp = 183–184 °C; ^1^H NMR (600 MHz, DMSO-*d*_6_): *δ* = 13.96 (s, 1H, OH), 7.92 (d, *J* = 5.9 Hz, 1H, NH), 7.85 (d, *J* = 8.5 Hz, 1H, ArH), 7.41 (d, *J* = 8.6 Hz, 2H, ArH), 7.22 (d, *J* = 7.1 Hz, 2H, ArH), 6.96 (d, *J* = 8.5 Hz, 2H, ArH), 6.11 (d, *J* = 5.8 Hz, 1H, O-CH), 3.74 (s, 3H, ArOCH_3_); ^13^C NMR (150 MHz, DMSO-*d*_6_): *δ* = 175.5, 157.0, 156.6, 140.2, 135.6, 128.4, 128.2, 122.7, 120.9, 118.6, 116.7, 116.7, 114.9, 114.9, 96.1, 55.4; HRMS (TOF ES+): *m*/*z* calcd for C_16_H_13_ClN_2_NaO_4_^+^ [(M+Na)^+^], 355.0456, found, 355.0460.

**2-Hydroxy-3-(2-(4-methoxyphenyl)hydrazineylidene)-7-(thiophen-2-yl)chroman-4-one (3l).** V_Petroleum ether_/V_Ethyl acetate_ = 10:1, R*_f_* = 0.2; Red solid: 82 mg (72%); mp = 197–198 °C; ^1^H NMR (500 MHz, DMSO-*d*_6_): *δ* = 14.04 (s, 1H, OH), 8.02 (d, *J* = 2.4 Hz, 1H, ArH), 7.92–7.90 (m, 1H, ArH), 7.83 (d, *J* = 5.9 Hz, 1H, NH), 7.55 (d, *J* = 5.1 Hz, 1H, ArH), 7.50 (d, *J* = 3.6 Hz, 1H, ArH), 7.43 (d, *J* = 9.0 Hz, 2H, ArH), 7.17–7.14 (m, 2H, ArH), 6.98 (d, *J* = 9.0 Hz, 2H, ArH), 6.12 (d, *J* = 5.9 Hz, 1H, O-CH), 3.76 (s, 3H, ArOCH_3_); ^13^C NMR (125 MHz, DMSO-*d*_6_): *δ* = 176.0, 156.5, 155.8, 142.1, 135.6, 133.0, 128.8, 128.7, 128.0, 125.6, 123.7, 122.4, 122.0, 119.6, 116.6, 116.6, 114.9, 114.9, 95.7, 55.4; HRMS (TOF ES+): *m*/*z* calcd for C_20_H_16_N_2_NaO_4_S^+^ [(M+Na)^+^], 403.0723, found, 403.0721.

**2-Hydroxy-6,7-dimethoxy-3-(2-(4-methoxyphenyl)hydrazineylidene)chroman-4-one (3m).** V_Petroleum ether_/V_Ethyl acetate_ = 5:1, R*_f_* = 0.1; Red solid: 100 mg (93%); mp = 179–180 °C; ^1^H NMR (600 MHz, DMSO-*d*_6_): *δ* = 13.90 (s, 1H, OH), 7.71 (d, *J* = 6.0 Hz, 1H, NH), 7.34 (d, *J* = 8.6 Hz, 2H, ArH), 7.22 (s, 1H, ArH), 6.96 (d, *J* = 8.5 Hz, 2H, ArH), 6.65 (s, 1H, ArH), 6.01 (d, *J* = 5.8 Hz, 1H, O-CH), 3.85 (s, 3H, ArOCH_3_), 3.78 (s, 3H, ArOCH_3_), 3.74 (s, 3H, ArOCH_3_); ^13^C NMR (150 MHz, DMSO-*d*_6_): *δ* = 175.8, 156.3, 156.0, 153.1, 144.7, 135.9, 129.0, 116.1, 116.1 114.9, 114.9, 113.7, 106.4, 101.5, 95.8, 56.3, 55.8, 55.4; HRMS (TOF ES+): *m*/*z* calcd for C_18_H_18_N_2_NaO_6_^+^ [(M+Na)^+^], 381.1057, found, 381.1061.

**6-Chloro-2-hydroxy-3-(2-(4-methoxyphenyl)hydrazineylidene)-7-methylchroman-4-one (3n).** V_Petroleum ether_/V_Ethyl acetate_ = 10:1, R*_f_* = 0.2; Red solid: 73 mg (70%); mp = 183–184 °C; ^1^H NMR (600 MHz, DMSO-*d*_6_): *δ* = 13.92 (s, 1H, OH), 7.82 (d, *J* = 5.9 Hz, 1H, NH), 7.74 (s, 1H, ArH), 7.42 (d, *J* = 8.6 Hz, 2H, ArH), 7.14 (s, 1H, ArH), 6.97 (d, *J* = 8.6 Hz, 2H, ArH), 6.07 (d, *J* = 5.8 Hz, 1H, O-CH), 3.75 (s, 3H, ArOCH_3_), 2.37 (s, 3H, ArCH_3_); ^13^C NMR (150 MHz, DMSO-*d*_6_): *δ* = 175.1, 156.5, 154.9, 144.3, 135.6, 128.4, 126.9, 125.6, 121.3, 121.2, 116.7, 116.7, 114.9, 114.9, 95.7, 55.4, 20.3; HRMS (TOF ES+): *m*/*z* calcd for C_17_H_15_ClN_2_NaO_4_^+^ [(M+Na)^+^], 369.0613, found, 369.0611.

**2-Hydroxy-3-(2-(4-methoxyphenyl)hydrazineylidene)-6,8-dimethylchroman-4-one (3o).** V_Petroleum ether_/V_Ethyl acetate_ = 10:1, R*_f_* = 0.2; Red solid: 88mg (90%); mp = 134–135 °C; ^1^H NMR (600 MHz, DMSO-*d*_6_): *δ* = 13.98 (s, 1H, OH), 7.62 (s, 1H, NH), 7.47 (s, 1H, ArH), 7.40 (d, *J* = 8.6 Hz, 2H, ArH), 7.29 (s, 1H, ArH), 6.96 (d, *J* = 8.6 Hz, 2H, ArH), 6.08 (s, 1H, O-CH), 3.74 (s, 3H, ArOCH_3_), 2.27 (s, 3H, ArCH_3_), 2.20 (s, 3H, ArCH_3_); ^13^C NMR (150 MHz, DMSO-*d*_6_): *δ* = 177.2, 156.3, 152.7, 137.8, 135.8, 130.5, 129.1, 127.3, 123.6, 121.4, 116.5, 116.5, 114.9, 114.9, 95.2, 55.4, 20.2, 15.6; HRMS (TOF ES+): *m*/*z* calcd for C_18_H_18_N_2_NaO_4_^+^ [(M+Na)^+^], 349.1159, found, 349.1169.

**6,8-Dichloro-2-hydroxy-3-(2-phenylhydrazineylidene)chroman-4-one (3p).** V_Petroleum ether_/V_Ethyl acetate_ = 10:1, R*_f_* = 0.2; Red solid: 66 mg (60%); mp = 198–199 °C; ^1^H NMR (600 MHz, DMSO-*d*_6_): *δ* = 13.97 (s, 1H, OH), 8.15 (d, *J* = 6.1 Hz, 1H, ArH), 7.94 (d, *J* = 2.7 Hz, 1H, ArH), 7.74 (d, *J* = 2.6 Hz, 1H, NH), 7.46 (d, *J* = 8.6 Hz, 2H, ArH), 6.98 (d, *J* = 8.5 Hz, 2H, ArH), 6.24 (d, *J* = 6.0 Hz, 1H, O-CH), 3.76 (s, 3H, ArOCH_3_); ^13^C NMR (150 MHz, DMSO-*d*_6_): *δ* = 173.9, 156.9, 150.8 135.4, 134.6, 127.7, 126.1, 124.4, 124.0, 124.0, 117.1, 117.1, 114.9, 114.9, 96.3, 55.4; HRMS (TOF ES+): *m*/*z* calcd for C_16_H_12_Cl_2_N_2_NaO_4_^+^ [(M+Na)^+^], 389.0066, found, 389.0069.

**3-Hydroxy-2-(2-(4-methoxyphenyl)hydrazineylidene)-2,3-dihydro-1H-benzo[*f*]chromen-1-one (3q).** V_Petroleum ether_/V_Ethyl acetate_ = 10:1, R*_f_* = 0.1; Red solid: 71 mg (68%); mp = 187–188 °C; ^1^H NMR (600 MHz, DMSO-*d*_6_): *δ* =14.04 (s, 1H, OH), 8.31 (d, *J* = 8.3 Hz, 1H, ArH), 8.01 (d, *J* = 6.1 Hz, 1H, NH), 7.97 (d, *J* = 8.1 Hz, 1H, ArH), 7.86 (d, *J* = 8.6 Hz, 1H, ArH), 7.73–7.71 (m, 1H, ArH), 7.66–7.62 (m, 2H, ArH), 7.43 (d, *J* = 9.0 Hz, 2H, ArH), 6.98 (d, *J* = 8.9 Hz, 2H, ArH), 6.33 (d, *J* = 6.0 Hz, 1H, O-CH), 3.75 (s, 3H, ArOCH_3_); ^13^C NMR (150 MHz, DMSO-*d*_6_): *δ* = 176.5, 156.3, 154.4, 136.9, 135.8, 129.9, 128.7, 128.2, 126.8, 124.9, 123.4, 121.8, 121.2, 116.5, 116.5, 116.3, 114.9, 114.9, 96.2, 55.4; HRMS (TOF ES+): *m*/*z* calcd for C_20_H_16_N_2_NaO_4_^+^ [(M+Na)^+^], 371.1002, found, 371.1010.

**2-Hydroxy-3-(2-(4-methoxyphenyl)hydrazineylidene)-2,3-dihydro-4*H*-benzo[*h*]chromen-4-one (3r).** V_Petroleum ether_/V_Ethyl acetate_ = 10:1, R*_f_* = 0.2; Red solid: 73 mg (70%); mp = 190–191 °C; ^1^H NMR (600 MHz, DMSO-*d*_6_): *δ* =14.03 (s, 1H, OH), 8.31 (d, *J* = 8.3 Hz, 1H, ArH), 8.01 (d, *J* = 6.2 Hz, 1H, NH), 7.97 (d, *J* = 8.2 Hz, 1H, ArH), 7.86 (d, *J* = 8.6 Hz, 1H, ArH), 7.72 (t, *J* = 7.5 Hz, 1H, ArH), 7.66–7.63 (m, 2H, ArH), 7.44 (d, *J* = 6.9 Hz, 2H, ArH), 6.98 (d, *J* = 8.8 Hz, 2H, ArH), 6.34 (d, *J* = 6.1 Hz, 1H, O-CH), 3.75 (s, 3H, ArOCH_3_); ^13^C NMR (150 MHz, DMSO-*d*_6_): *δ* = 176.5, 156.3, 154.4, 136.9, 135.8, 129.9, 128.7, 128.2, 126.8, 124.9, 123.4, 121.8, 121.2, 116.5, 116.5, 116.3, 114.9, 114.9, 96.2, 55.4; HRMS (TOF ES+): *m*/*z* calcd for C_20_H_16_N_2_NaO_4_^+^ [(M+Na)^+^], 371.1002, found, 371.1011.

**2-Hydroxy-3-(2-phenylhydrazineylidene)chroman-4-one (3s).** V_Petroleum ether_/V_Ethyl acetate_ = 10:1, R*_f_* = 0.2; Red solid: 78 mg (86%); mp = 170–171 °C; ^1^H NMR (500 MHz, DMSO-*d*_6_): *δ* = 13.78 (s, 1H, OH), 7.90–7.88 (m, 1H, ArH), 7.84 (d, *J* = 5.7 Hz, 1H, NH), 7.65–7.61 (m, 1H, ArH), 7.46–7.44 (m, 2H, ArH), 7.40–7.37 (m, 2H, ArH), 7.20–7.16 (m, 1H, ArH), 7.12–7.08 (m, 2H, ArH), 6.10 (d, *J* = 5.8 Hz, 1H, O-CH); ^13^C NMR (125 MHz, DMSO-*d*_6_): *δ* = 177.3, 156.6, 142.2, 136.4, 129.9, 129.6, 129.6, 126.6, 124.0, 122.3, 121.8, 118.8, 115.1, 115.1, 95.5; HRMS (TOF ES+): *m*/*z* calcd for C_15_H_13_N_2_O_3_ ^+^ [(M+H)^+^], 269.0921, found, 269.0926.

**3-(2-(4-Bromophenyl)hydrazineylidene)-2-hydroxychroman-4-one (3t).** V_Petroleum ether_/V_Ethyl acetate_ = 10:1, R*_f_* = 0.2; Red solid: 81 mg (78%); mp = 161–162 °C; ^1^H NMR (500 MHz, DMSO-*d*_6_): *δ* = 13.64 (s, 1H, OH), 7.89 (s, 2H, ArH), 7.64 (s, 1H, NH), 7.54 (s, 2H, ArH), 7.43 (s, 2H, ArH), 7.14 (d, *J* = 35.4 Hz, 2H, ArH), 6.08 (s, 1H, O-CH); ^13^C NMR (125 MHz, DMSO-*d*_6_): *δ* = 177.4, 156.7, 141.8, 136.6, 132.3, 132.3, 130.5, 126.7, 122.3, 121.8, 118.9, 117.1, 117.1, 115.6, 95.6; HRMS (TOF ES+): *m*/*z* calcd for C_15_H_11_BrN_2_NaO_3_^+^ [(M+Na)^+^], 368.9845, found, 368.9847.

**2-Hydroxy-3-(2-(4-(trifluoromethoxy)phenyl)hydrazineylidene)chroman-4-one (3u).** V_Petroleum ether_/V_Ethyl acetate_ = 10:1, R*_f_* = 0.2; Red solid: 91 mg (86%); mp = 173–174 °C; ^1^H NMR (500 MHz, DMSO-*d*_6_): *δ* = 13.65 (s, 1H, OH), 7.90–7.87 (m, 2H, ArH), 7.66–7.62 (m, 1H, NH), 7.56 (d, *J* = 8.8 Hz, 2H, ArH), 7.37 (d, *J* = 8.5 Hz, 2H, ArH), 7.18 (t, *J* = 7.5 Hz, 1H, ArH), 7.11 (d, *J* = 8.3 Hz, 1H, ArH), 6.09 (d, *J* = 5.3 Hz, 1H, O-CH); ^13^C NMR (125 MHz, DMSO-*d*_6_): *δ* = 177.4, 156.7, 144.2, 141.6, 136.6, 130.6, 126.7, 122.5, 122.5, 122.3, 121.8, 120.2 (C-F, *J* = 256.0 Hz), 118.9, 116.4, 116.4, 95.6; ^19^F NMR (470 MHz, DMSO-*d*_6_) *δ* = -57.1; HRMS (TOF ES+): *m*/*z* calcd for C_16_H_11_F_3_N_2_NaO_4_^+^ [(M+Na)^+^], 375.0563, found, 375.0563.

**3-(2-(4-Ethynylphenyl)hydrazineylidene)-2-hydroxychroman-4-one (3v).** V_Petroleum ether_/V_Ethyl acetate_ = 10:1, R*_f_* = 0.1; Red solid: 70 mg (80%); mp = 179–180 °C; ^1^H NMR (600 MHz, DMSO-*d*_6_): *δ* = 13.66 (s, 1H, OH), 7.91 (d, *J* = 5.8 Hz, 1H, NH), 7.89–7.87 (m, 1H, ArH), 7.66–7.63 (m, 1H, ArH), 7.48–7.45 (m, 4H, ArH), 7.19–7.17 (m, 1H, ArH), 7.11 (d, *J* = 8.3 Hz, 1H, ArH), 6.09 (d, *J* = 5.8 Hz, 1H, O-CH), 4.15 (s, 1H, C≡CH); ^13^C NMR (150 MHz, DMSO-*d*_6_): *δ* = 177.5, 156.8, 142.7, 136.7, 133.2, 133.2, 130.9, 126.7, 122.4, 121.8, 119.0, 116.7, 115.2, 115.2, 95.6, 83.6, 80.6; HRMS (TOF ES+): *m*/*z* calcd for C_17_H_12_N_2_NaO_3_^+^ [(M+Na)^+^], 315.0740, found, 315.0746.

**3-(2-(3-Bromophenyl)hydrazineylidene)-2-hydroxychroman-4-one (3w).** V_Petroleum ether_/V_Ethyl acetate_ = 10:1, R*_f_* = 0.2; Red solid: 90 mg (87%); mp = 191–192 °C; ^1^H NMR (500 MHz, DMSO-*d*_6_): *δ* = 13.54 (s, 1H, OH), 7.89–7.87 (m, 2H, ArH), 7.70 (s, 1H, NH), 7.64 (t, *J* = 7.9 Hz, 1H, ArH), 7.45 (d, *J* = 8.1 Hz, 1H, ArH), 7.33–7.29 (m, 1H, ArH), 7.24 (d, *J* = 7.9 Hz, 1H, ArH), 7.19–7.16 (m, 1H, ArH), 7.11 (d, *J* = 8.4 Hz, 1H, ArH), 6.10 (d, *J* = 5.8 Hz, 1H, O-CH); ^13^C NMR (125 MHz, DMSO-*d*_6_): *δ* = 177.5, 156.7, 144.1, 136.6, 131.4, 130.9, 126.7, 126.1, 122.5, 122.3, 121.7, 118.9, 117.4, 114.2, 95.5; HRMS (TOF ES+): *m*/*z* calcd for C_15_H_12_N_2_BrO_3_^+^ [(M+H)^+^], 347.0026, found, 347.0031.

**3-(2-(2-Hydroxy-4-oxochroman-3-ylidene)hydrazineyl)benzonitrile (3x).** V_Petroleum ether_/V_Ethyl acetate_ = 10:1, R*_f_* = 0.1; Yellow solid: 77 mg (88%); mp = 208–209 °C; ^1^H NMR (500 MHz, DMSO-*d*_6_): *δ* = 13.51 (s, 1H, OH), 7.93 (d, *J* = 5.7 Hz, 1H, NH), 7.90–7.88 (m, 2H, ArH), 7.81–7.78 (m, 1H, ArH), 7.67–7.63 (m, 1H, ArH), 7.58–7.54 (m, 1H, ArH), 7.51–7.49 (m, 1H, ArH), 7.20–7.17 (m, 1H, ArH), 7.12 (d, *J* = 8.3 Hz, 1H, ArH), 6.11 (d, *J* = 5.6 Hz, 1H, O-CH); ^13^C NMR (125 MHz, DMSO-*d*_6_): *δ* = 177.7, 156.9, 143.4, 136.8, 131.4, 130.8, 126.9, 126.7, 122.4, 121.7, 119.7, 119.0, 118.7, 118.0, 112.2, 95.6; HRMS (TOF ES+): *m*/*z* calcd for C_16_H_12_N_3_O_3_^+^ [(M+H)^+^], 294.0873, found, 294.0880.

**3-(2-(3,5-Dimethylphenyl)hydrazineylidene)-2-hydroxychroman-4-one (3y).** V_Petroleum ether_/V_Ethyl acetate_ = 10:1, R*_f_* = 0.2; Red solid: 76 mg (86%); mp = 167–168 °C; ^1^H NMR (500 MHz, DMSO-*d*_6_): *δ*= 13.80 (s, 1H, OH), 7.89–7.86 (m, 1H, ArH), 7.81 (d, *J* = 5.8 Hz, 1H, NH), 7.64–7.60 (m, 1H, ArH), 7.19–7.16 (m, 1H, ArH), 7.10 (d, *J* = 8.3 Hz, 1H, ArH), 7.05 (s, 2H, ArH), 6.74 (s, 1H, ArH), 6.09 (d, *J* = 5.7 Hz, 1H, O-CH), 2.27 (s, 6H, ArCH_3_); ^13^C NMR (125 MHz, DMSO-*d*_6_): *δ* = 177.2, 156.5, 142.1, 138.9, 138.9, 136.3, 129.7, 126.6, 125.8, 122.2, 121.8, 118.8, 112.8, 112.8, 95.5, 21.0, 21.0; HRMS (TOF ES+): *m*/*z* calcd for C_17_H_17_N_2_O_3_^+^ [(M+H)^+^], 297.1234, found, 297.1239.

**2-Hydroxy-3-(2-(2-methylnaphthalen-1-yl)hydrazineylidene)chroman-4-one (3z).** V_Petroleum ether_/V_Ethyl acetate_ = 10:1, R*_f_* = 0.2; Red solid: 71 mg (84%); mp = 206–207 °C; ^1^H NMR (500 MHz, DMSO-*d*_6_): *δ* = 14.09 (s, 1H, OH), 7.91 (d, *J* = 7.8 Hz, 1H, ArH), 7.86 (d, *J* = 5.8 Hz, 1H, NH), 7.64–7.61 (m, 2H, ArH), 7.30–7.23 (m, 2H, ArH), 7.19–7.16 (m, 1H, ArH), 7.11 (d, *J* = 8.3 Hz, 1H, NH), 7.04–7.01 (m, 1H, ArH), 6.12 (d, *J* = 5.8 Hz, 1H, O-CH), 2.32 (s, 3H, ArCH_3_); ^13^C NMR (125 MHz, DMSO-*d*_6_): *δ* = 177.5, 156.6, 140.0, 136.5, 130.9, 130.8, 127.4, 126.7, 123.9, 123.5, 122.3, 121.8, 118.8, 113.3, 95.4, 16.5; HRMS (TOF ES+): *m*/*z* calcd for C_16_H_15_N_2_O_3_^+^ [(M+H)^+^], 283.1077, found, 283.1083.

**3-(2-(2-Chlorophenyl)hydrazineylidene)-2-hydroxychroman-4-one (3a’).** V_Petroleum ether_/V_Ethyl acetate_ = 10:1, R*_f_* = 0.2; Yellow solid: 79 mg (87%); mp = 208–209 °C; ^1^H NMR (500 MHz, DMSO-*d*_6_): *δ* = 14.07 (s, 1H, OH), 7.95 (d, *J* = 5.9 Hz, 1H, NH), 7.93–7.91 (m, 1H, ArH), 7.74 (d, *J* = 8.3 Hz, 1H, ArH), 7.67–7.64 (m, 1H, ArH), 7.52 (d, *J* = 8.0 Hz, 1H, ArH), 7.41 (t, *J* = 7.9 Hz, 1H, ArH), 7.20–7.17 (m, 1H, ArH), 7.13–7.09 (m, 2H, ArH), 6.12 (d, *J* = 5.9 Hz, 1H, O-CH); ^13^C NMR (125 MHz, DMSO-*d*_6_): *δ* = 178.1, 156.8, 138.5, 136.9, 132.3, 129.7, 128.7, 126.9, 124.5, 122.4, 121.6, 119.0, 118.9, 115.0, 95.3; HRMS (TOF ES+): *m*/*z* calcd for C_15_H_12_ClN_2_O_3_^+^ [(M+H)^+^], 303.0531, found, 303.0538.

**2-Hydroxy-3-(2-(naphthalen-2-yl)hydrazineylidene)chroman-4-one (3b’).** V_Petroleum ether_/V_Ethyl acetate_ = 10:1, R*_f_* = 0.2; Red solid: 81 mg (85%); mp = 177–178 °C; ^1^H NMR (500 MHz, DMSO-*d*_6_): *δ* = 14.00 (s, 1H, OH), 7.95 (d, *J* = 9.0 Hz, 1H, NH), 7.92–7.86 (m, 5H, ArH), 7.72 (d, *J* = 8.9 Hz, 1H, ArH), 7.66–7.63 (m, 1H, ArH), 7.51–7.48 (m, 1H, ArH), 7.42–7.39 (m, 1H, ArH), 7.21–7.18 (m, 1H, ArH), 7.12 (d, *J* = 8.3 Hz, 1H, ArH), 6.16 (d, *J* = 5.7 Hz, 1H, O-CH); ^13^C NMR (125 MHz, DMSO-*d*_6_): *δ* =177.3, 156.6, 140.0, 136.5, 133.7, 130.4, 130.4, 129.6, 127.9, 127.3, 127.0, 126.7, 124.9, 122.3, 121.9, 118.9, 115.8, 110.9, 95.6; HRMS (TOF ES+): *m*/*z* calcd for C_19_H_15_N_2_O_3_^+^ [(M+H)^+^], 319.1077, found, 319.1086.

**Methyl-3-(2-(2-hydroxy-4-oxochroman-3-ylidene)hydrazineyl)thiophene-2-carboxylate (3c’).** V_Petroleum ether_/V_Ethyl acetate_ = 10:1, R*_f_* = 0.1; Red solid: 81 mg (81%); mp = 202–203 °C; ^1^H NMR (500 MHz, DMSO-*d*_6_): *δ* = 14.49 (s, 1H, OH), 7.97 (d, *J* = 5.8 Hz, 1H, NH), 7.92–7.90 (m, 2H, ArH), 7.67–7.63 (m, 1H, ArH), 7.46 (d, *J* = 5.5 Hz, 1H, ArH), 7.20–7.17 (m, 1H, ArH), 7.11 (d, *J* = 8.3 Hz, 1H, ArH), 6.06 (d, *J* = 5.9 Hz, 1H, O-CH), 3.88 (s, 3H, O-CH_3_); ^13^C NMR (125 MHz, DMSO-*d*_6_): *δ* = 177.5, 162.7, 156.8, 148.9, 136.9, 134.1, 132.2, 126.9, 122.4, 121.8, 119.0, 118.3, 106.7, 95.4, 52.2; HRMS (TOF ES+): *m*/*z* calcd for C_15_H_13_N_2_O_5_S^+^ [(M+H)^+^], 333.0540, found, 333.0545.

***N*-(4-(2-(2-Hydroxy-4-oxochroman-3-ylidene)hydrazineyl)phenyl)acetamide (3d’).** V_Petroleum ether_/V_Ethyl acetate_ = 3:1, R*_f_* = 0.2; Red solid: 73 mg (75%); mp = 230–231 °C; ^1^H NMR (600 MHz, DMSO-*d*_6_): *δ* = 13.93 (s, 1H, OH), 10.00 (s, 1H, Ar-NH), 7.88–7.86(m, 1H, ArH), 7.81 (d, *J* = 5.7 Hz, 1H, N=NH), 7.63–7.62 (m, 1H, ArH), 7.61–7.58 (m, 2H, ArH), 7.41–7.39 (m, 2H, ArH), 7.19–7.16 (m, 1H, ArH), 7.10 (d, *J* = 8.2 Hz, 1H, ArH), 6.08 (d, *J* = 5.8 Hz, 1H, O-CH), 2.03 (s, 3H, C-CH_3_); ^13^C NMR (150 MHz, DMSO-*d*_6_): *δ* = 176.9, 168.3, 156.5, 137.5, 136.3, 136.0, 129.4, 126.6, 122.3, 121.9, 120.2, 120.2, 118.8, 115.6, 115.6, 95.5, 24.1; HRMS (TOF ES+): *m*/*z* calcd for C_17_H_15_N_3_NaO_4_^+^ [(M+Na)^+^], 348.0955, found, 348.0962.

***N*-(4-(2-(2-Hydroxy-4-oxochroman-3-ylidene)hydrazineyl)phenyl)-4,7,7-trimethyl-3-oxo-2-oxabicyclo[2.2.1]heptane-1-carboxamide (3e’).** V_Petroleum ether_/V_Ethyl acetate_ = 5:1, R*_f_* = 0.2; Red solid: 86 mg (61%); mp = 230–231 °C; ^1^H NMR (500 MHz, DMSO-*d*_6_): *δ* = 13.88 (s, 1H, OH), 9.88 (s, 1H, C-NH), 7.88 (d, *J* = 7.8 Hz, 1H, ArH), 7.84 (d, *J* = 5.7 Hz, 1H, N=NH), 7.73 (d, *J* = 8.4 Hz, 2H, ArH), 7.62 (s, 1H, ArH)), 7.42 (d, *J* = 8.5 Hz, 2H, ArH), 7.19–7.16 (m, 1H, ArH), 7.10 (d, *J* = 8.4 Hz, 1H, ArH), 6.08 (s, 1H, O-CH), 2.03–1.87 (m, 3H, C-CH_2_), 1.58 (s, 1H C-CH_2_), 1.05–1.02 (m, 6H, C-CH_3_), 0.90 (s, 3H, C-CH_3_); ^13^C NMR (125 MHz, DMSO-*d*_6_): *δ* = 178.2, 177.1, 165.3, 156.6, 138.6, 136.4, 134.3, 129.7, 126.6, 122.3, 122.3, 121.9, 121.4, 118.9, 115.2, 115.2, 95.5, 92.0, 54.7, 53.7, 30.1, 28.5, 16.7, 16.5, 9.7.; HRMS (TOF ES+): *m*/*z* calcd for C_25_H_26_N_3_O_6_^+^ [(M+H)^+^], 464.1816, found, 464.1822.

**3-(2-Benzyl-2-(4-methoxyphenyl)hydrazineylidene)-2-(benzyloxy)-6,8-dimethylchroman-4-one (4).** V_Petroleum ether_/V_Ethyl acetate_ = 5:1, R*_f_* = 0.2; Red solid: 23 mg (15%); mp = 140–141 °C; ^1^H NMR (600 MHz, DMSO-*d*_6_): *δ* = 7.66 (d, *J* = 8.4 Hz, 2H, ArH), 7.53 (d, *J* = 7.5 Hz, 2H, ArH), 7.45–7.42 (m, 2H, ArH), 7.40–7.37 (m, 1H, ArH), 7.33–7.27 (m, 6H, ArH), 7.13 (s, 1H, ArH), 7.08 (d, *J* = 8.5 Hz, 2H, ArH), 6.61 (s, 1H, O-CH), 5.89 (d, *J* = 12.0 Hz, 1H, N-CH_2_), 5.76 (d, *J* = 12.0 Hz, 1H, N-CH_2_), 4.75 (d, *J* = 3.3 Hz, 2H, N-CH_2_), 3.83 (s, 3H, ArOCH_3_), 2.23 (d, *J* = 13.7 Hz, 6H, ArCH_3_); ^13^C NMR (150 MHz, DMSO-*d*_6_): *δ* = 161.0, 151.9, 148.7, 146.9, 137.5, 137.2, 134.1, 130.5, 128.6, 128.6, 128.4, 128.3, 128.3, 128.2, 128.2, 128.1, 128.1, 127.8, 127.8, 125.8, 124.0, 124.0, 122.1, 118.7, 114.7, 114.7, 93.7, 77.3, 69.6, 55.6, 20.5, 15.2; HRMS (TOF ES+): *m*/*z* calcd for C_32_H_30_N_2_O_4_Na^+^ [(M+Na)^+^], 529.2098, found, 529.2111.

**3-(2-(4-Methoxyphenyl)hydrazineylidene)-6,8-dimethylchromane-2,4-dione (5).** V_Petroleum ether_/V_Ethyl acetate_ = 5:1, R*_f_* = 0.2; Red solid: 57 mg (59%); mp = 133–134 °C; ^1^H NMR (600 MHz, DMSO-*d*_6_): *δ* = 8.42 (s, 1H, NH), 7.32 (s, 2H, ArH), 7.13–7.10 (m, 2H, ArH), 6.88 (d, *J* = 9.1 Hz, 2H, ArH), 3.69 (s, 3H, ArOCH_3_), 2.34 (s, 3H, CH_3_), 2.14 (s, 3H, CH_3_); ^13^C NMR (125 MHz, DMSO-*d*_6_): *δ* = 182.5, 160.2, 155.7, 143.4, 136.0, 134.8, 133.7, 131.2, 130.5, 128.6, 121.7, 116.4, 116.4, 114.7, 114.7, 55.3, 20.3, 16.0; HRMS (TOF ES+): *m*/*z* calcd for C_18_H_16_N_2_O_4_Na^+^ [(M+Na)^+^], 347.1002, found, 347.0993.

## 4. Conclusions

In summary, we have developed a novel method for the simultaneous functionalization of the *α*- and *β*-positions of *o*-hydroxyaryl enaminones, utilizing water as a nucleophile and diazonium salts as an electrophilic reagent, to efficiently construct a 2,3-disubstituted chromone skeleton. This approach features a broad substrate scope, is environmentally friendly, and operates efficiently without the need for any metal catalysts. The scalability of the reaction was demonstrated through gram-scale experiments, highlighting its potential for industrial synthesis applications.

## Figures and Tables

**Table 1:** Optimization of reaction conditions *^a^*.

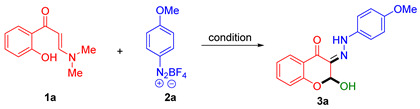						
Entry	Additive (Equiv.)	Solvent	*T* (^o^C)	Atm.	Time (h)	Yield (%) *^b^*
1	H_2_O (20)	THF	r.t.	Air	4	65
2	H_2_O (20)	2-MeTHF	r.t.	Air	4	50
3	H_2_O (20)	1,4-dioxane	r.t.	Air	4	53
4	H_2_O (20)	1,3-dioxolane	r.t.	Air	4	30
5	H_2_O (20)	EA	r.t.	Air	4	48
6	H_2_O (20)	CHCl_3_	r.t.	Air	4	trace
7	H_2_O (20)	DCE	r.t.	Air	4	41
8	H_2_O (20)	Toluene	r.t.	Air	4	n.r.
9	H_2_O (20)	PhCl	r.t.	Air	4	n.r.
10	H_2_O (20)	DMF	r.t.	Air	4	61
11	H_2_O (20)	DMA	r.t.	Air	4	60
**12**	**H_2_O (20)**	**NMP**	**r.t.**	**Air**	**4**	**88**
13	/	NMP	r.t.	Air	4	40
14	H_2_O (10)	NMP	r.t.	Air	4	70
15	H_2_O (30)	NMP	r.t.	Air	4	72
16 *^c^*	H_2_O (20)	NMP	r.t.	Air	4	76
17 *^d^*	H_2_O (20)	NMP	r.t.	Air	4	84
18	H_2_O (20)	NMP	30	Air	4	82
19	H_2_O (20)	NMP	50	Air	4	85
20	H_2_O (20)	NMP	r.t.	N_2_	4	86
21	H_2_O (20)	NMP	r.t.	O_2_	4	87
22	H_2_O (20)	NMP	r.t.	Air	2	75
23	H_2_O (20)	NMP	r.t.	Air	6	83

*^a^* Reaction conditions: **1a** (0.1 mmol), **2a** (0.15 mmol), and additive in 1 mL of solvent. *^b^* Isolated yields. *^c^* **2a** (1.0 equiv.) *^d^* **2a** (2.0 equiv.).

## Data Availability

Data are contained within the article or Supplementary Materials.

## Supplementary Materials

The following supporting information can be downloaded at: https://www.mdpi.com/article/10.3390/molecules30061194/s1. Figure S1: X-Ray crystal structure of 3a; Table S1: Crystal data and structure refinement for 3a; Figures S2–S69: 1H NMR and 13C NMR spectra.
