# Supplementary material for: Eco-Friendly and Efficient Synthesis of 2-Hydroxy-3-Hydrazono-Chromones Through α,β-C(sp2)–H Bond Difunctionalization/Chromone Annulation Reaction of o-Hydroxyaryl Enaminones, Water, and Aryldiazonium Salts
Source: Molecules. 2025 Mar 7;30(6):1194. doi: 10.3390/molecules30061194 (PMC11944599; doi:10.3390/molecules30061194)
Supplement: Supplementary file 1 [file molecules-30-01194-s001.zip › molecules-3519068-supplementary.pdf]

Supporting Information

Eco-Friendly and Efficient Synthesis of 2-Hydroxy-3-hydrazono-chromones through *α,β*-C(sp^2)−H Bond Difunctionalization/Chromone Annulation Reaction of *o*-Hydroxyaryl Enaminones, Water, and Aryldiazonium Salts

Xiaohong Wang [1,†], Menglin Peng [1,†], Yijin Wang [1], Siyu Song [1], Ying Xu [1], Li Chen [1,*] and Fuchao Yu [1,*]

[1] Faculty of Life Science and Technology, Kunming University of Science and Technology, Kunming 650500, China;

1. X-ray Structure and Data of 3a (CCDC 2425569).

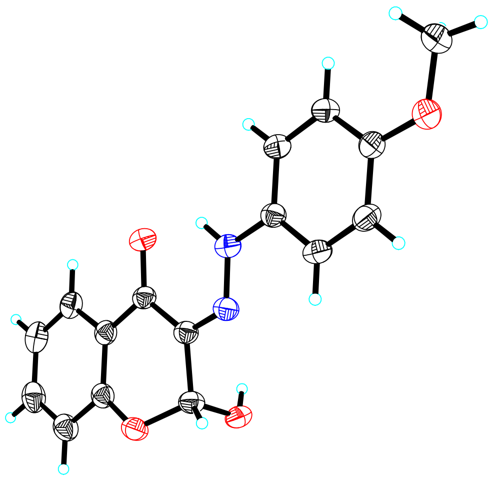

Figure S1 X-Ray crystal structure of **3a.**

Table S1 Crystal data and structure refinement for **3a.**

Empirical formula	$C_{16}H_{14}N_2O_4$	
Formula weight	298.29	
Temperature	299.00 K	
Crystal system, space group	monoclinic, P21/n	
Unit cell dimensions	a = 9.4649(3) A	alpha = 90 deg.
	b = 11.9597(4) A	beta = 106.3270 deg.
	c = 12.8114(4) A	gamma = 90 deg.
Volume	1391.73(8) A^3	
Z, Calculated density	4, 1.424 Mg/m^3	
Absorption coefficient	0.865 mm^-1	
F(000)	624.0	
Theta range for data collection	10.318 to 136.724 deg.	
Limiting indices	-11<=h<=10, -14<=k<=14, -15<=l<=15	
Reflections collected / unique	15064 / 2530 [R(int) = 0.0372, R(sigma)= 0.0262]	
Data/restraints/parameters	2530 / 0 / 202	
Goodness-of-fit on F^2	1.068	
Final R indices [I>2sigma(I)]	R1 = 0.0349, wR2 = 0.1048	
R indices (all data)	R1 = 0.0395, wR2 = 0.1070	
Largest diff. peak and hole	0.17 and -0.17 e.A^-3	

2. ^{1}H NMR and ^{13}C NMR spectra for spectroscopic data.

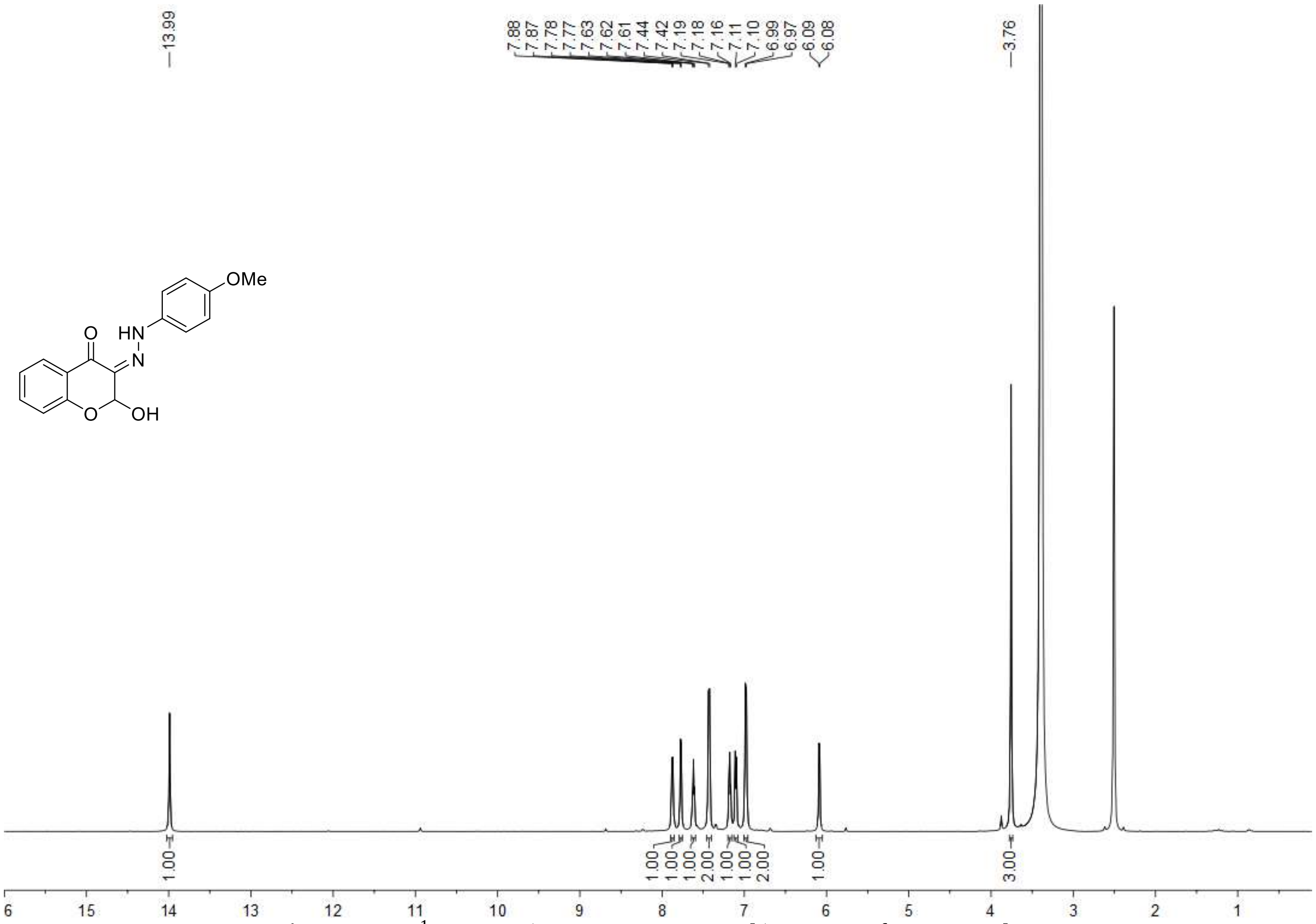

Figure S2. ^{1}H NMR (600 MHz, DMSO-d_6) spectra of compound **3a**

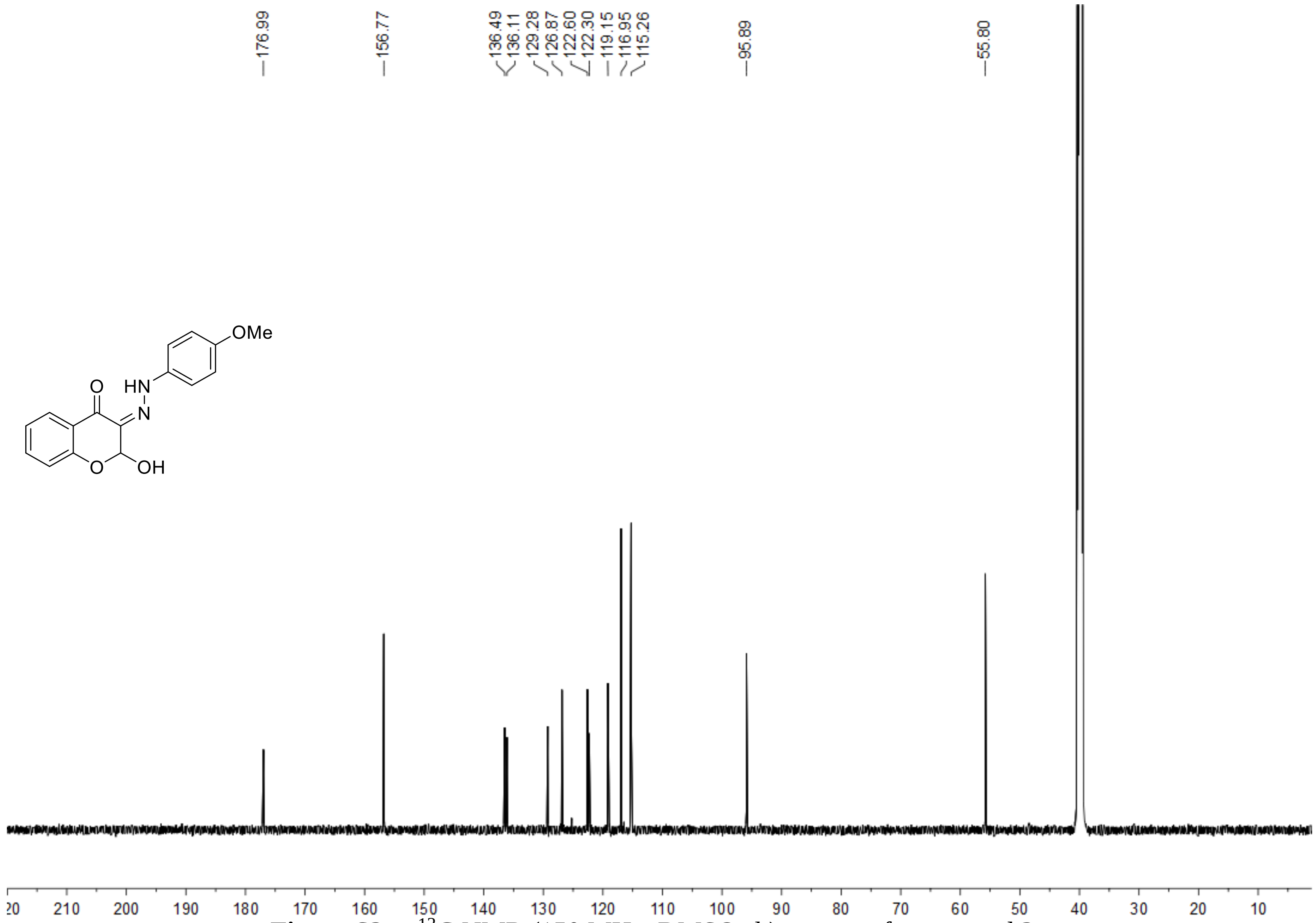

Figure S3. ^{13}C NMR (150 MHz, DMSO-d_6) spectra of compound **3a**

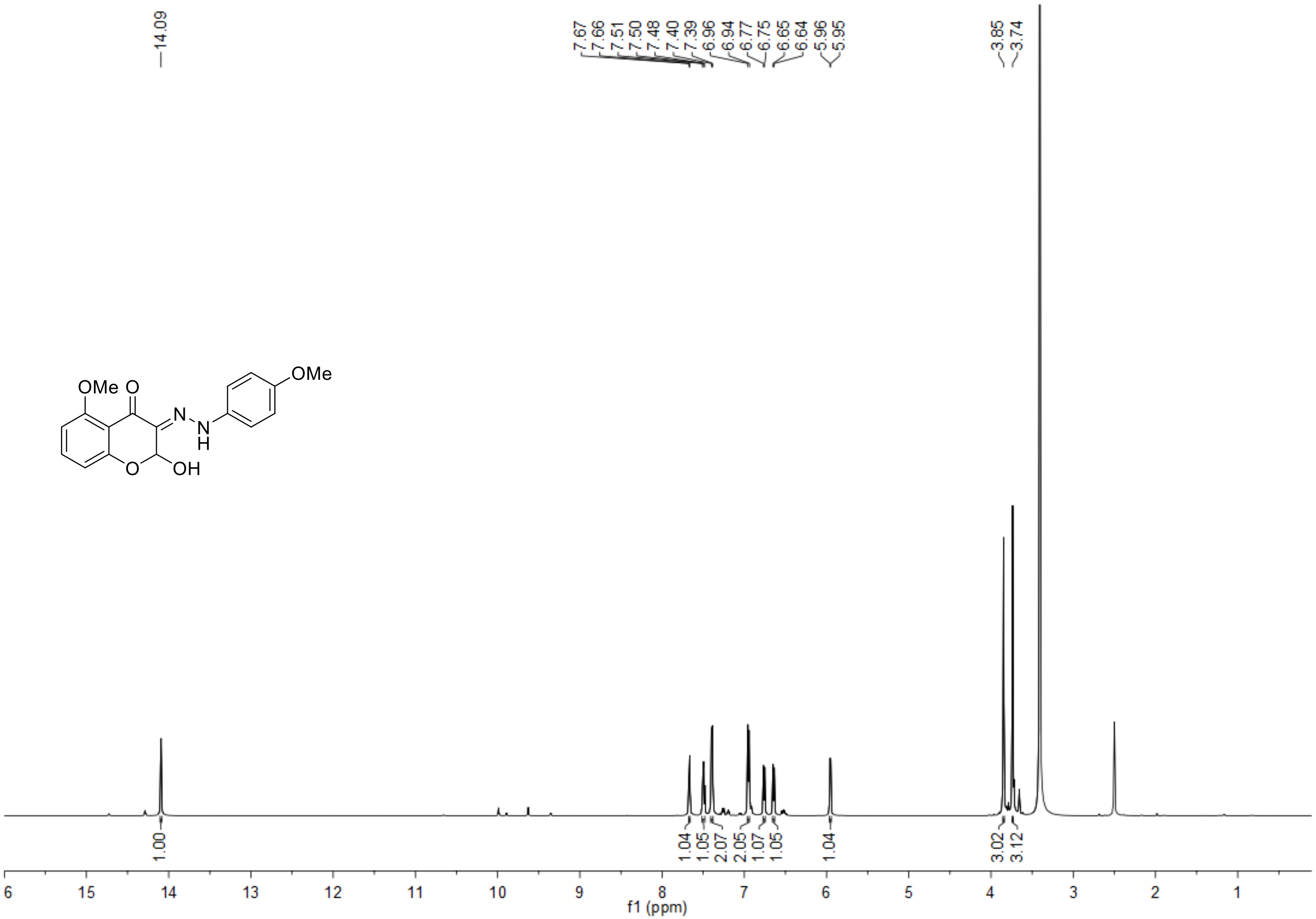

Figure S4. ^{1}H NMR (600 MHz, DMSO-d_6) spectra of compound **3b**

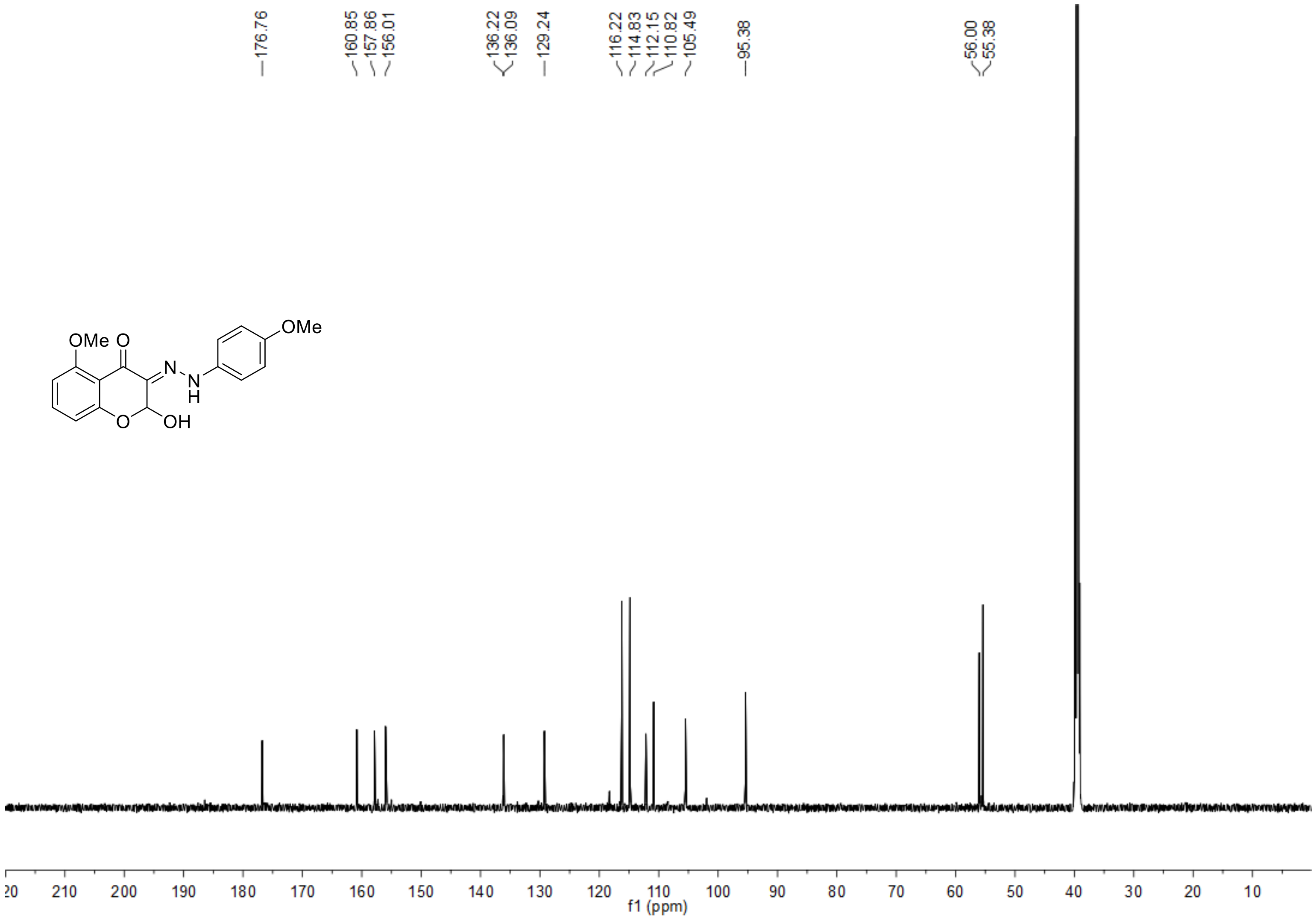

Figure S5. ^{13}C NMR (150 MHz, DMSO-d_6) spectra of compound **3b**

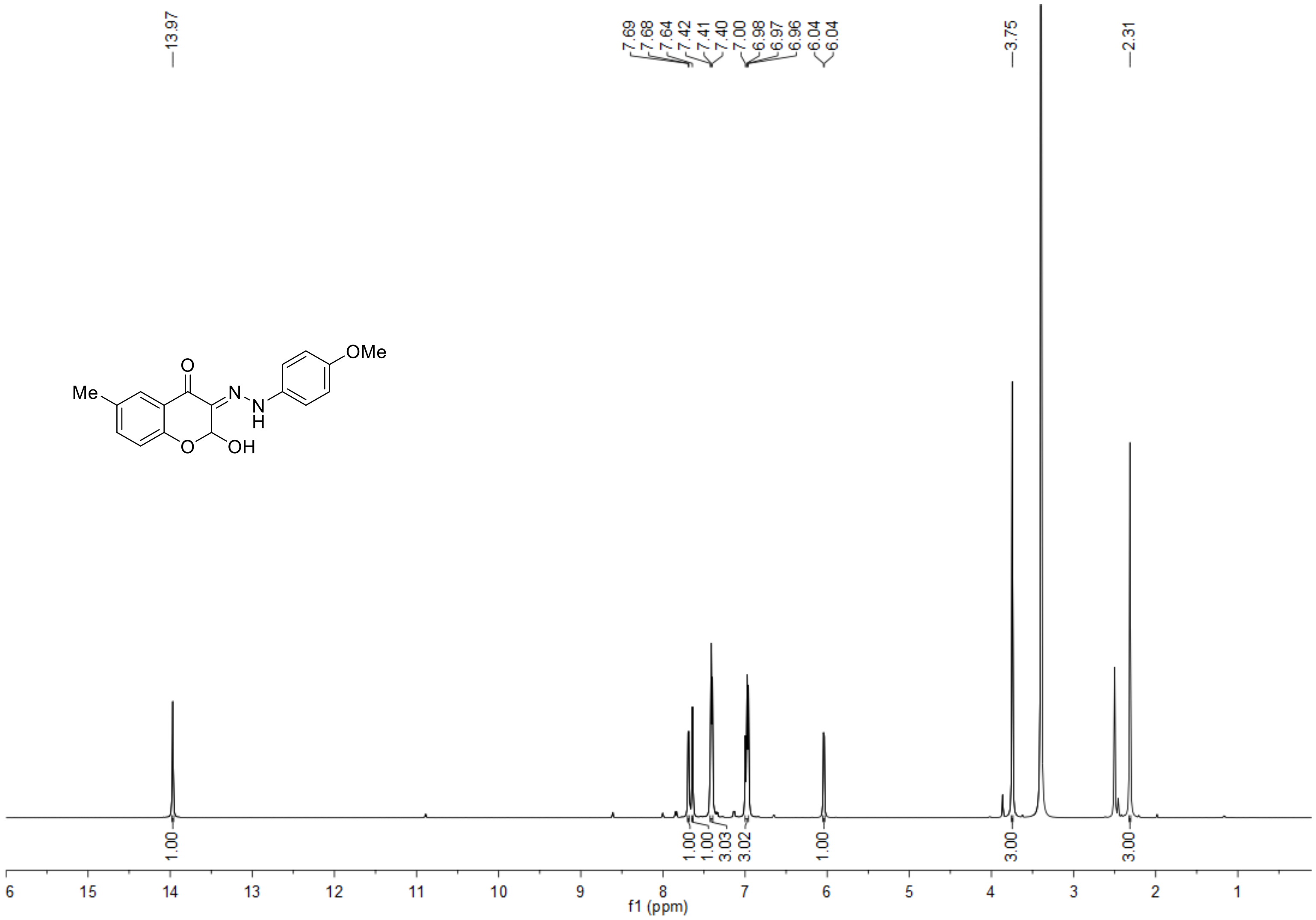

Figure S6. ¹H NMR (600 MHz, DMSO-d_6) spectra of compound **3c**

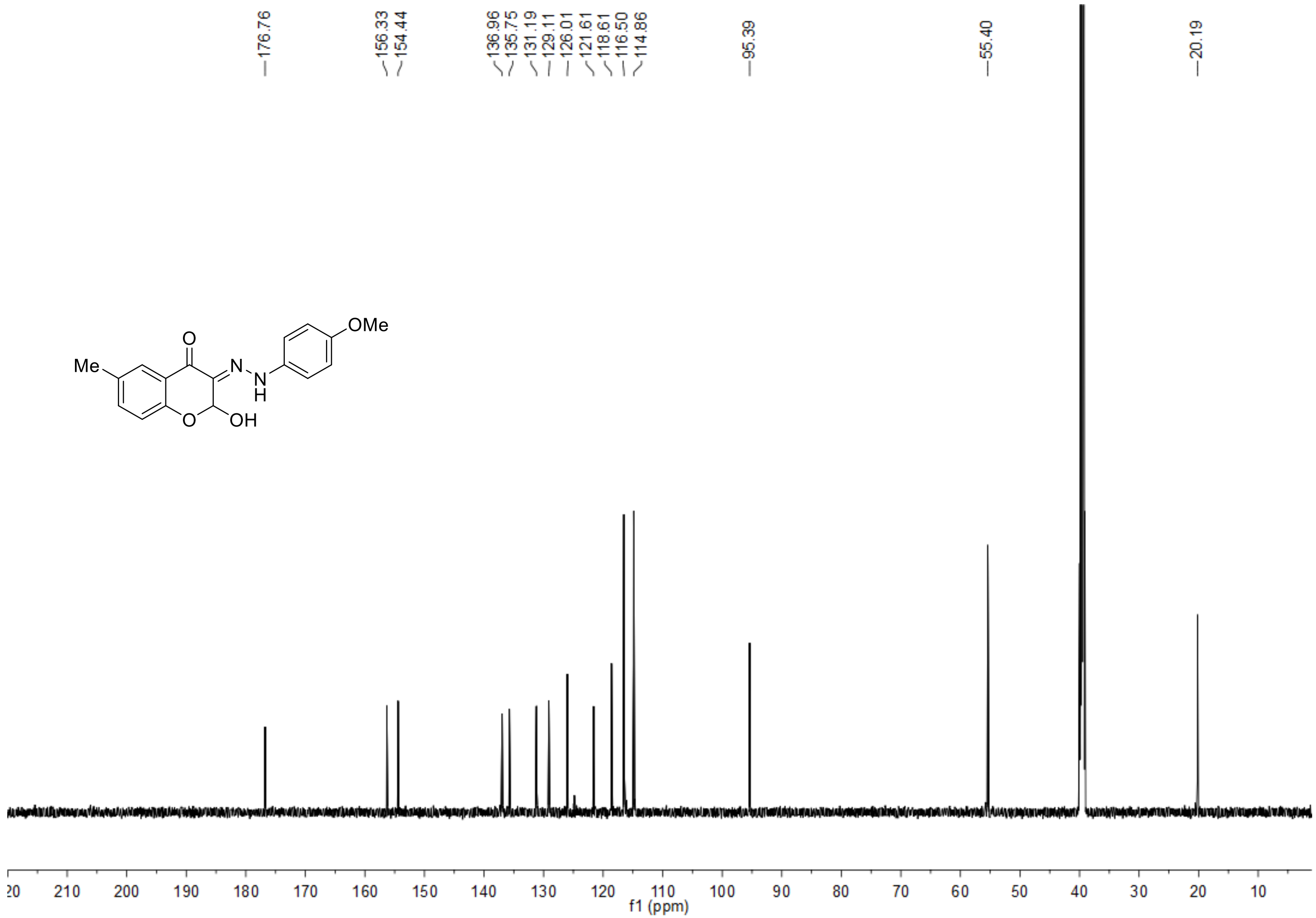

Figure S7. ^{13}C NMR (150 MHz, DMSO-d_6) spectra of compound **3c**

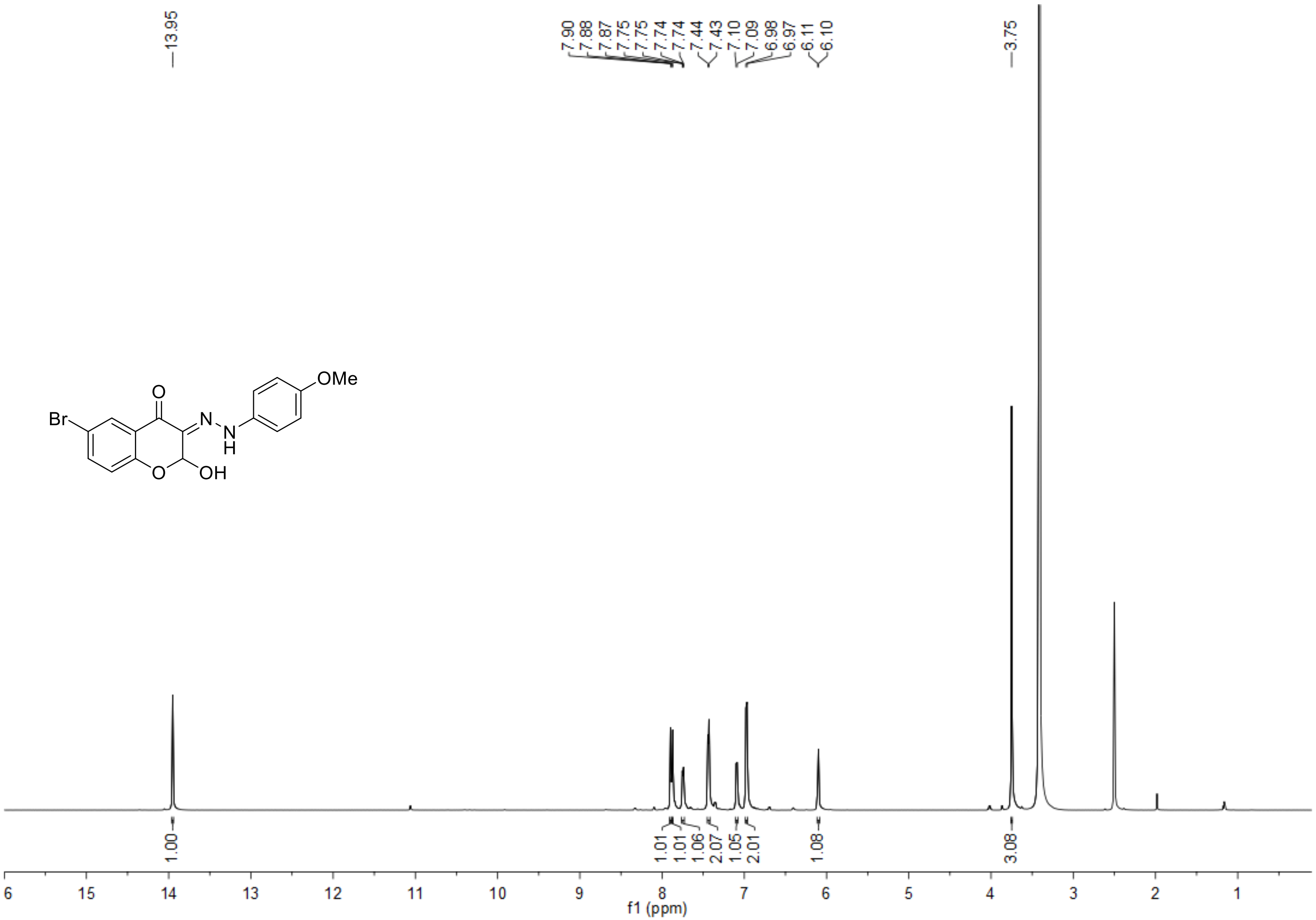

Figure S8. ^{1}H NMR (600 MHz, DMSO-d_6) spectra of compound **3d**

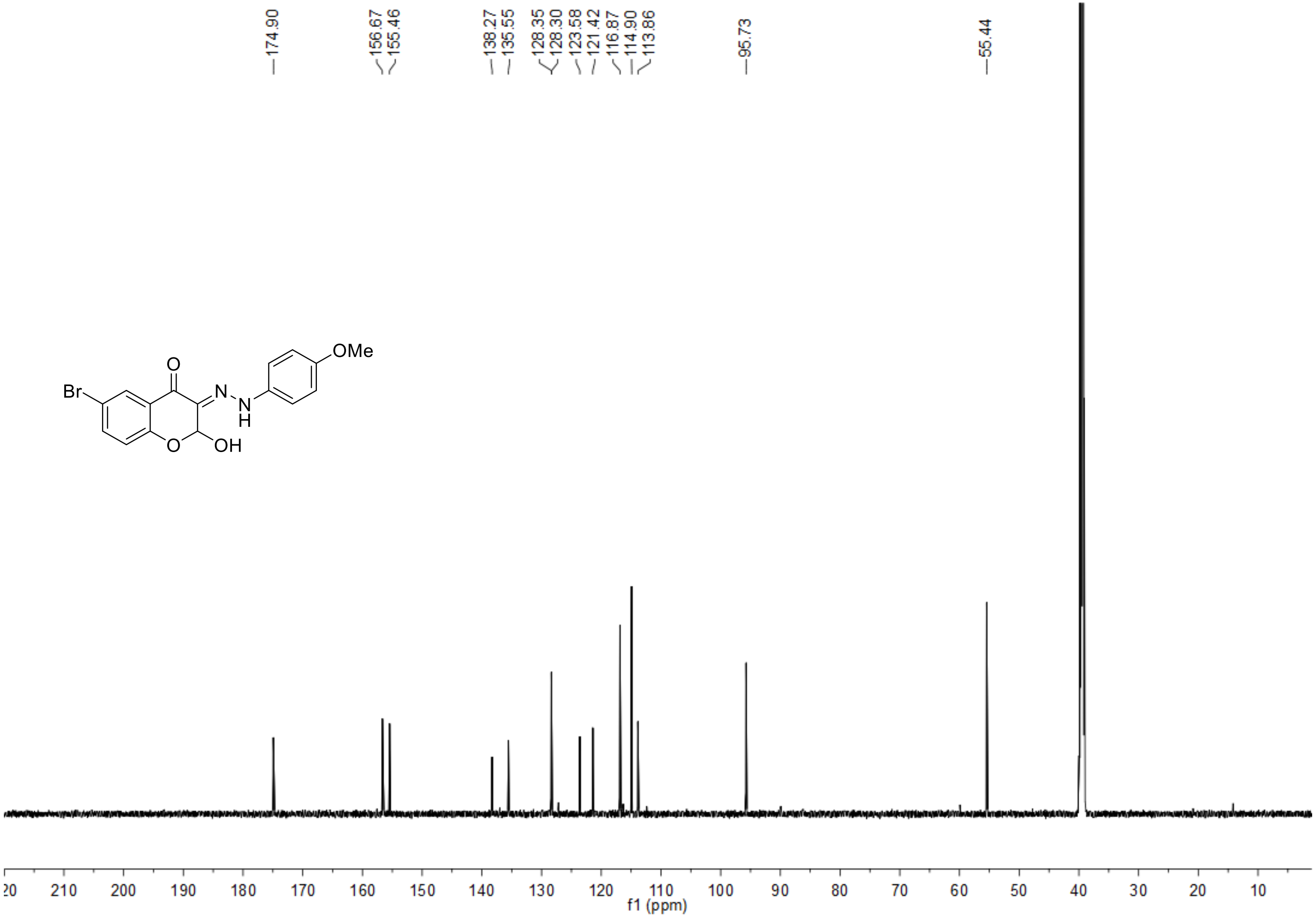

Figure S9. ^{13}C NMR (150 MHz, DMSO-d_6) spectra of compound **3d**

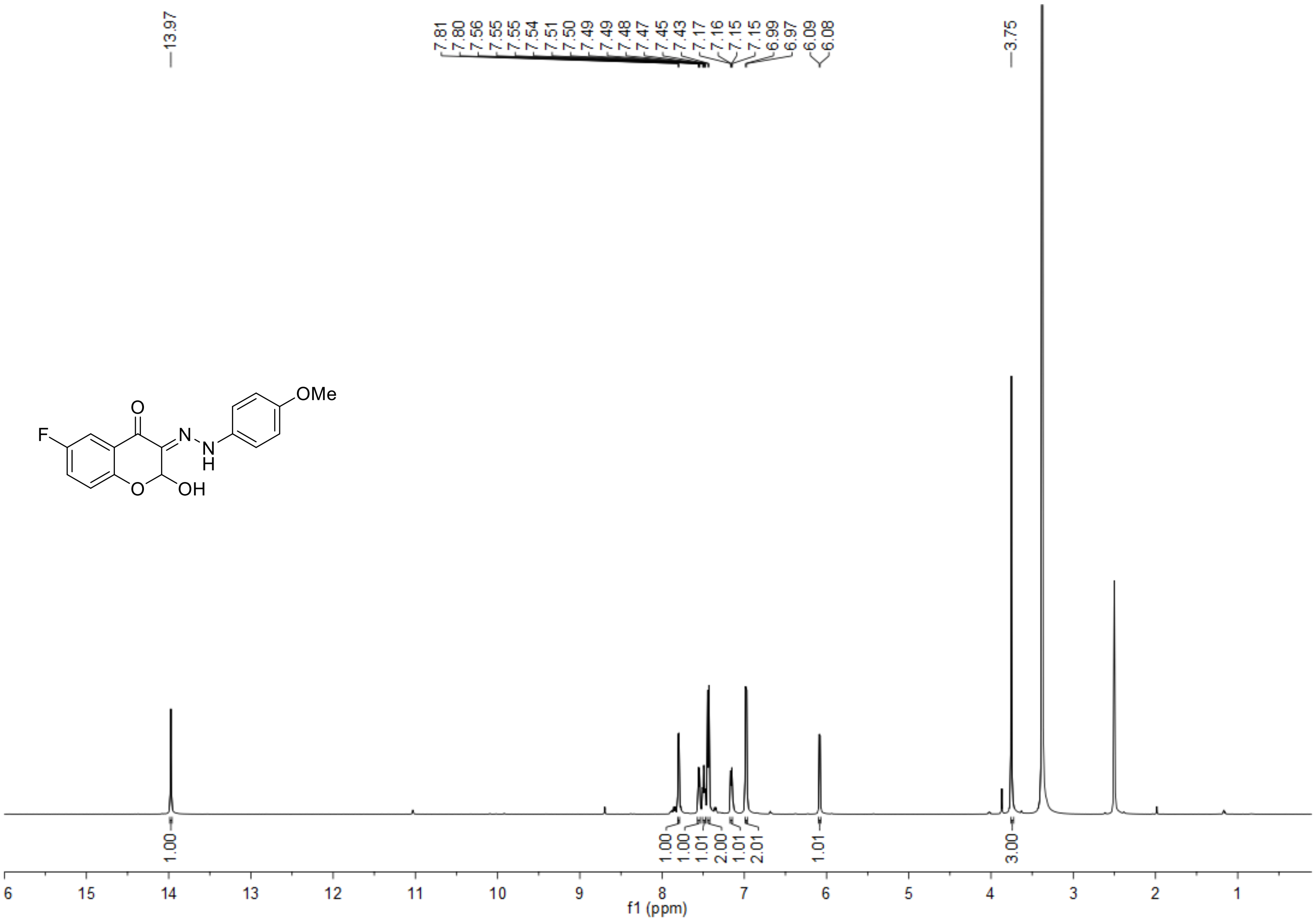

Figure S10. ^{1}H NMR (600 MHz, DMSO-d_6) spectra of compound **3e**

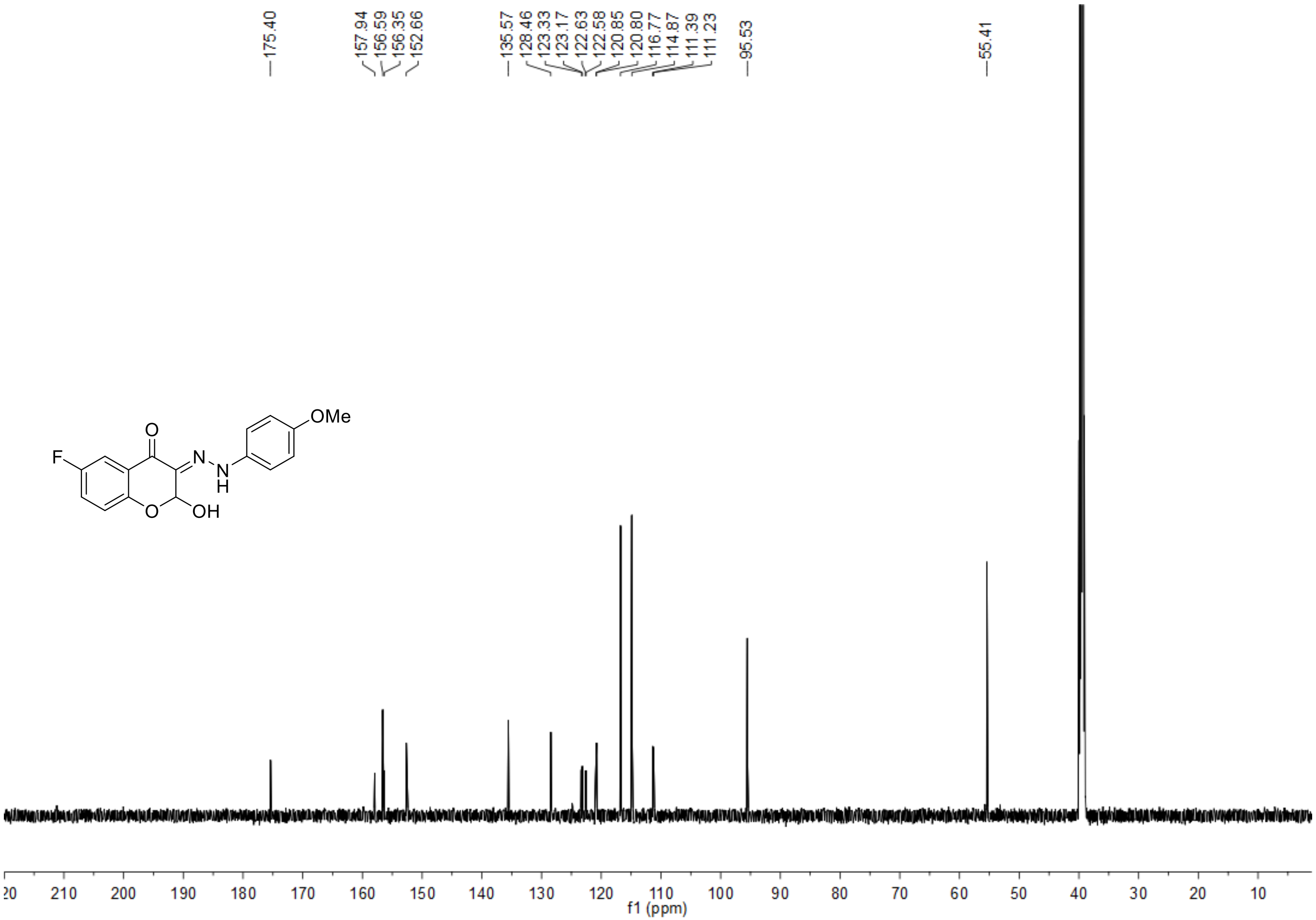

Figure S11. ^{13}C NMR (150 MHz, DMSO-d_6) spectra of compound **3e**

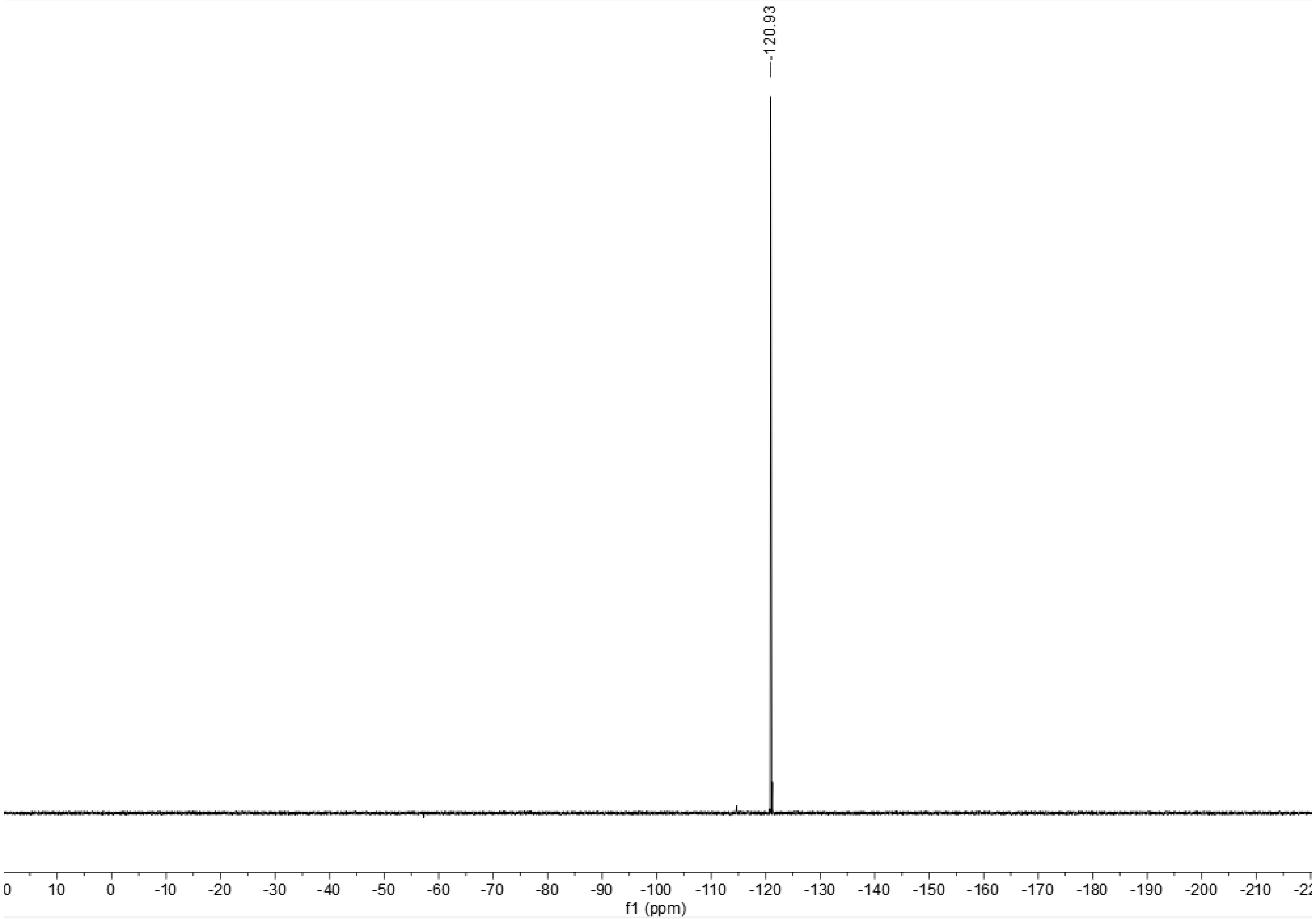

Figure S12. ^{19}F NMR (470 MHz, DMSO-d_6) spectra of compound **3e**

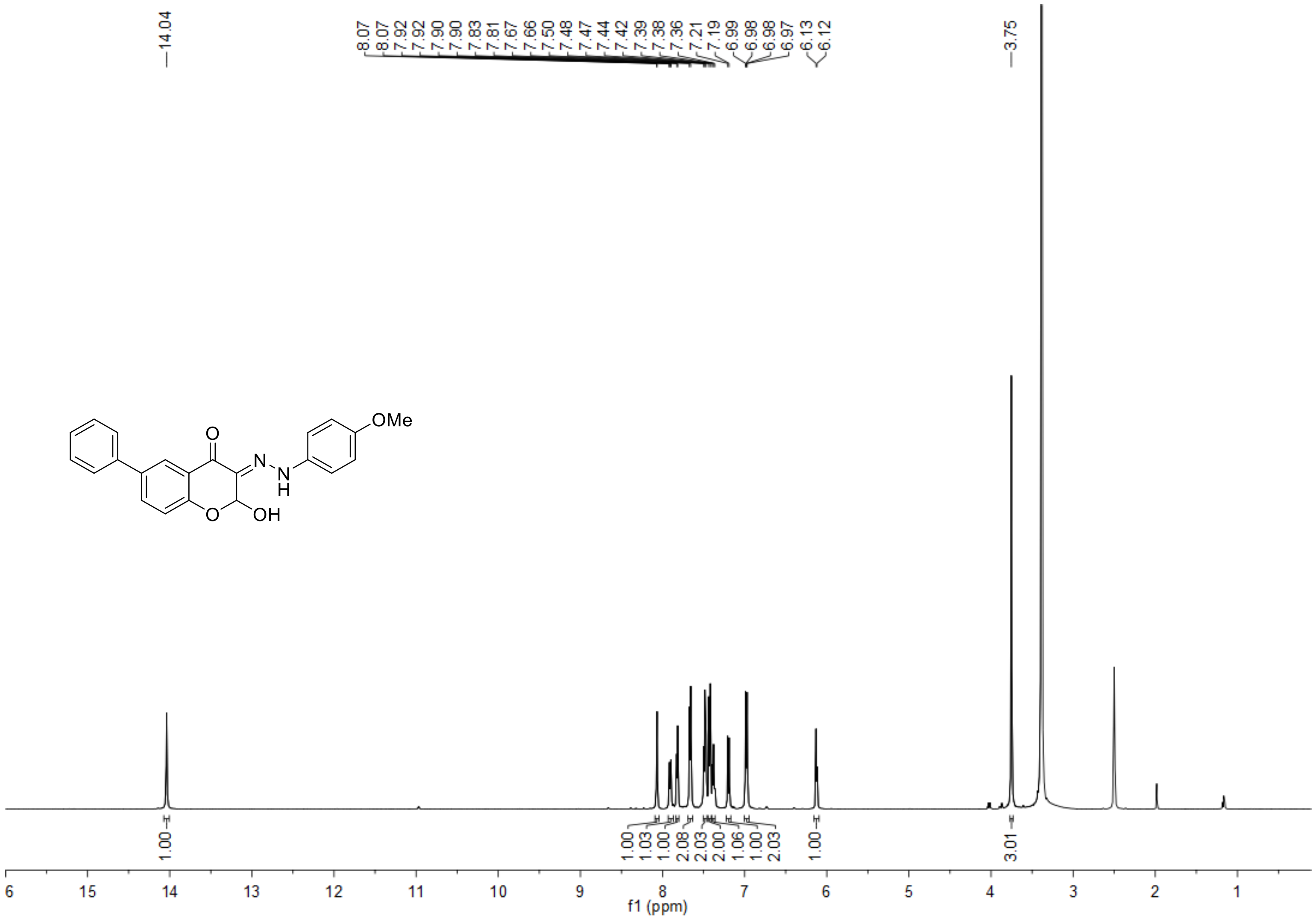

Figure S13. ^{1}H NMR (500 MHz, DMSO-d_6) spectra of compound **3f**

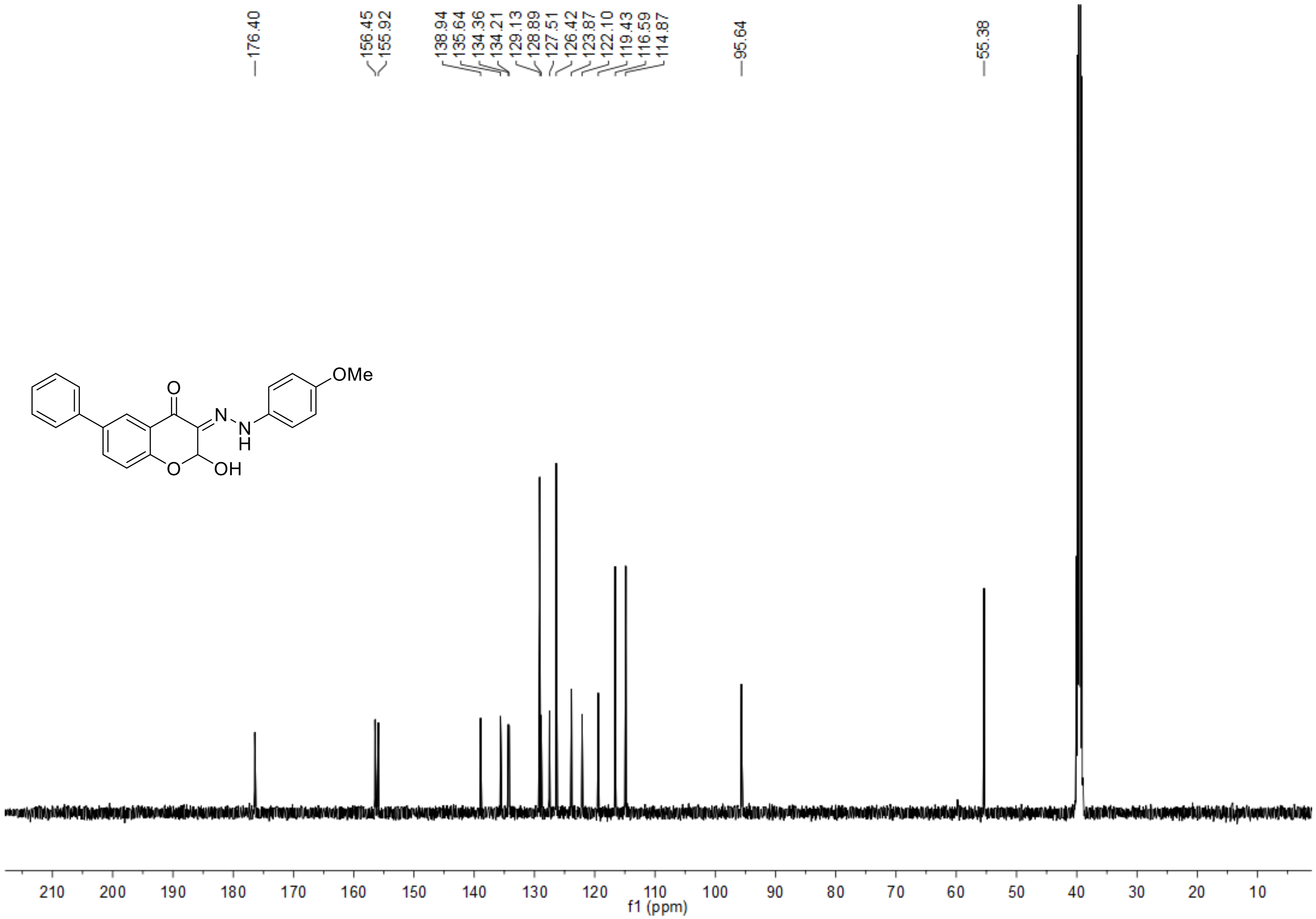

Figure S14. ^{13}C NMR (125 MHz, DMSO-d_6) spectra of compound **3f**

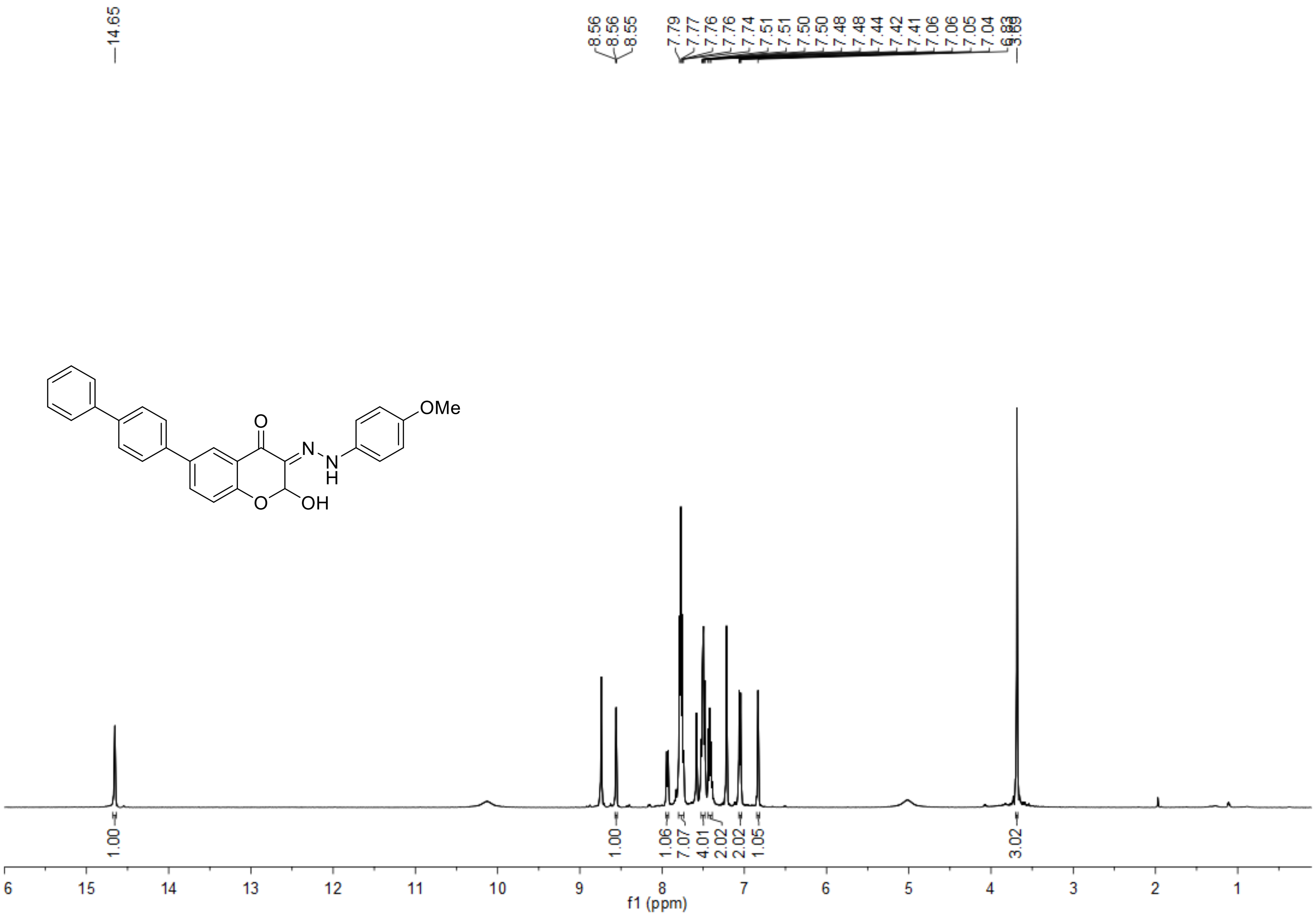

Figure S15. ^{1}H NMR (500 MHz, Pyridine-d_5) spectra of compound **3g**

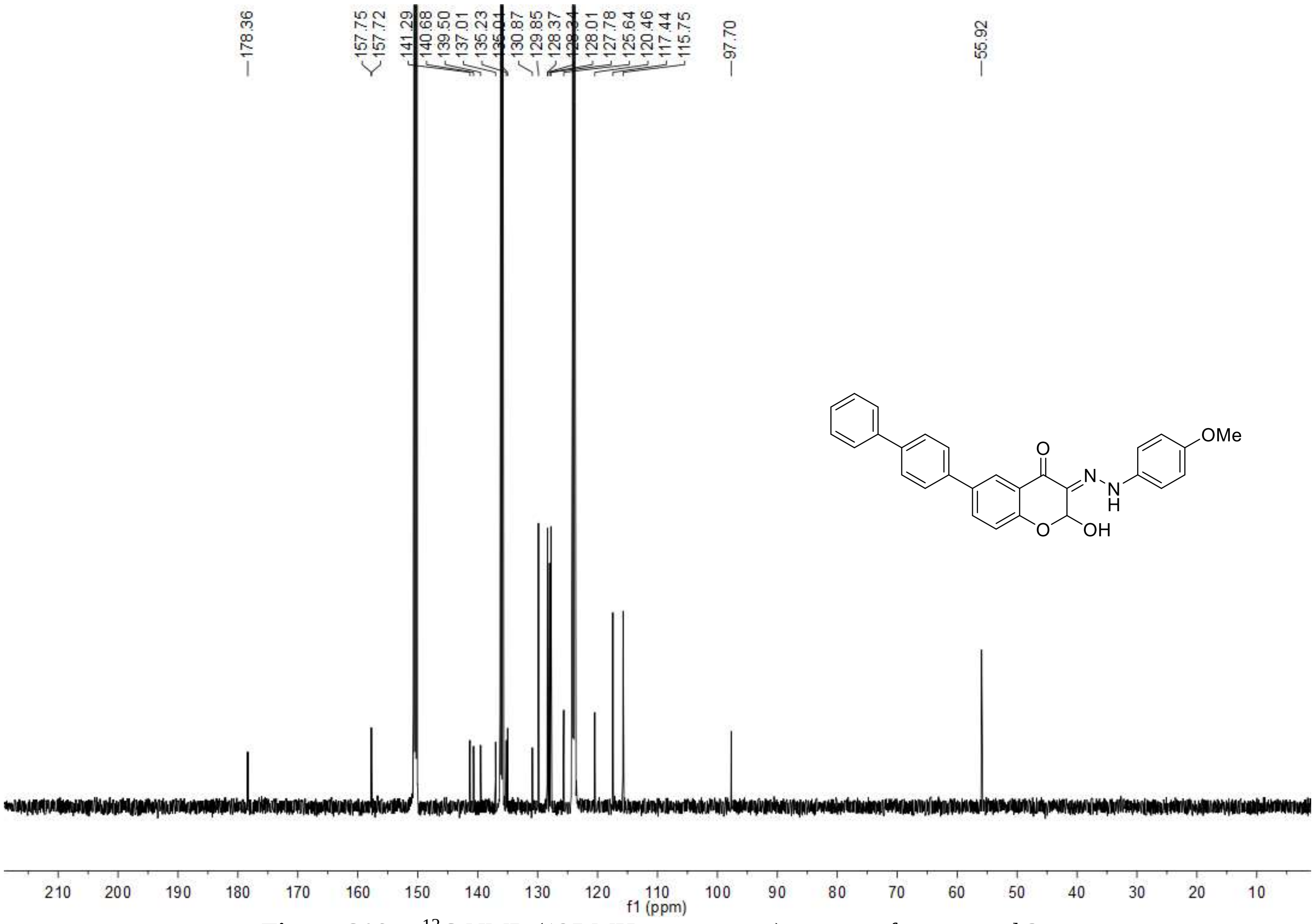

Figure S16. ^{13}C NMR (125 MHz, Pyridine-d_5) spectra of compound **3g**

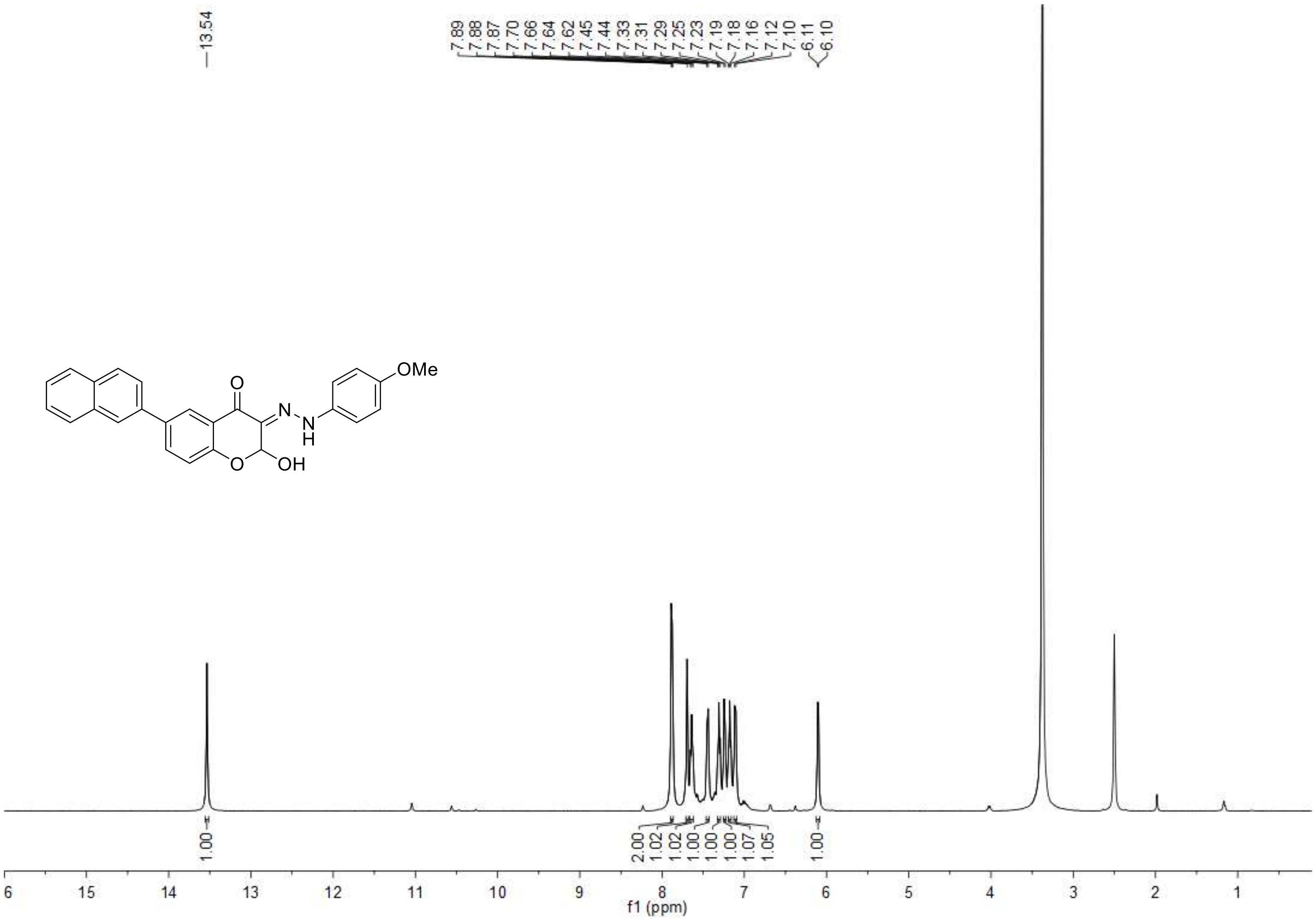

Figure S17. ^{1}H NMR (600 MHz, DMSO-d_6) spectra of compound **3h**

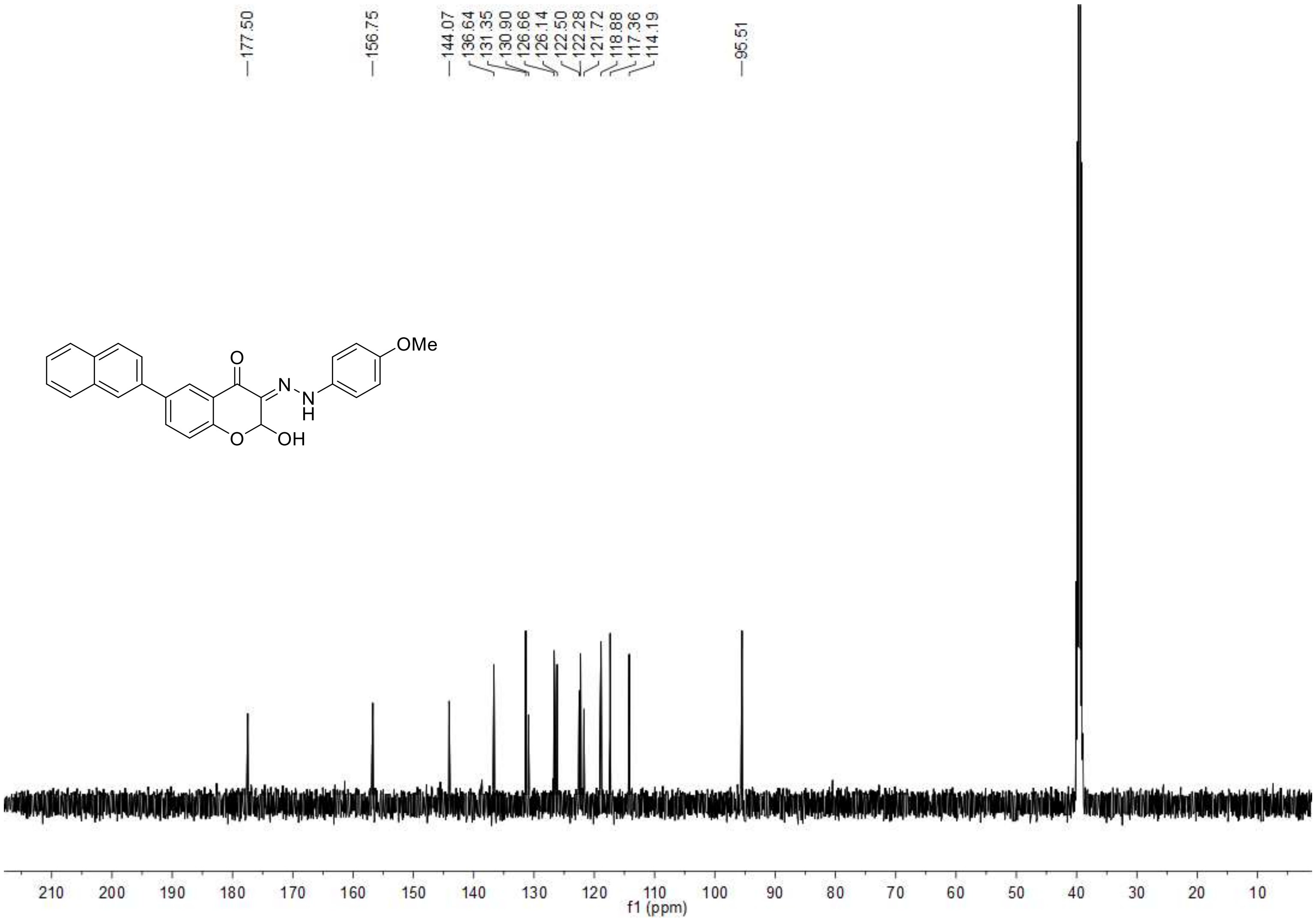

Figure S18. ^{13}C NMR (150 MHz, DMSO-d_6) spectra of compound **3h**

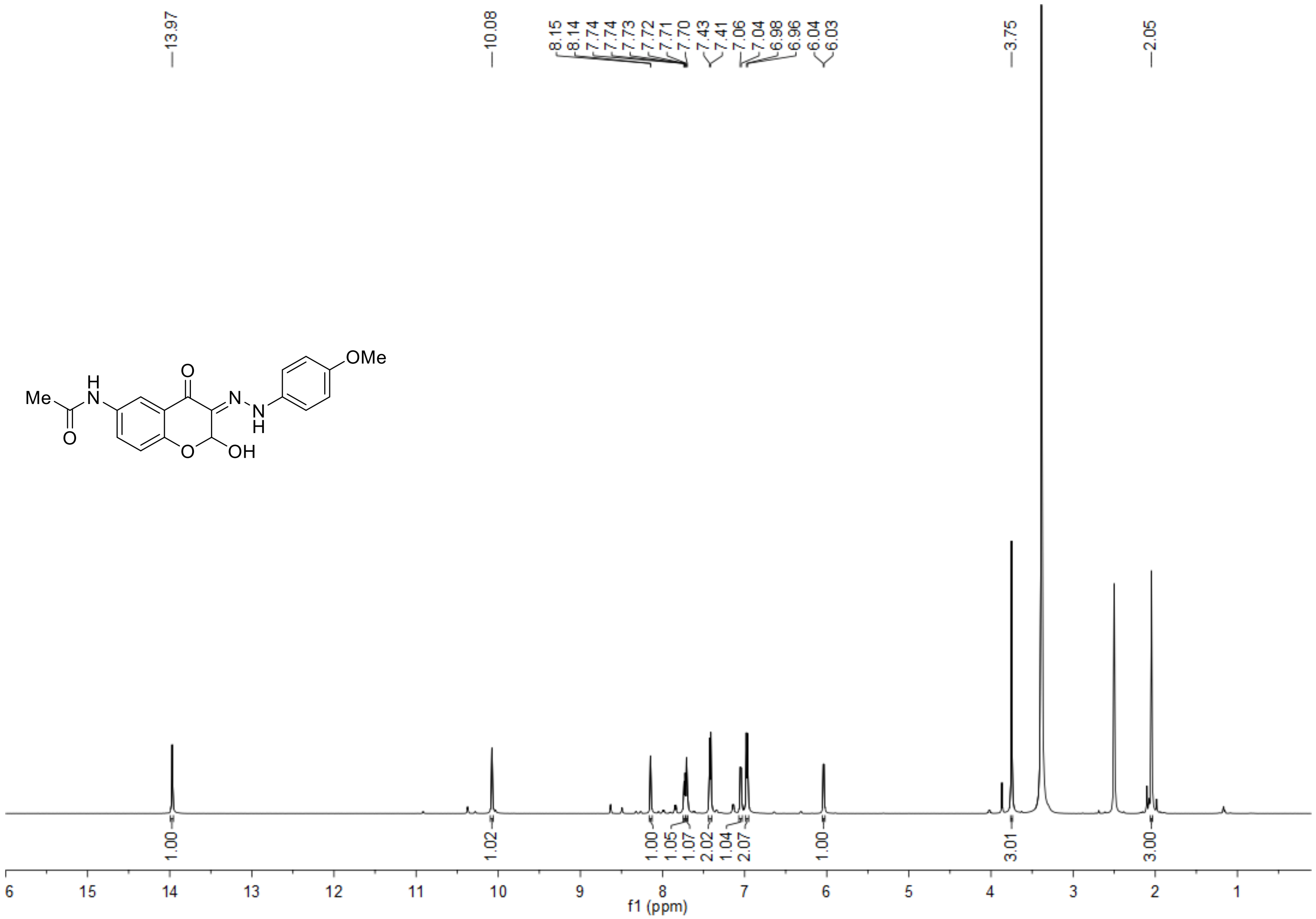

Figure S19. ^{1}H NMR (600 MHz, DMSO-d_6) spectra of compound **3i**

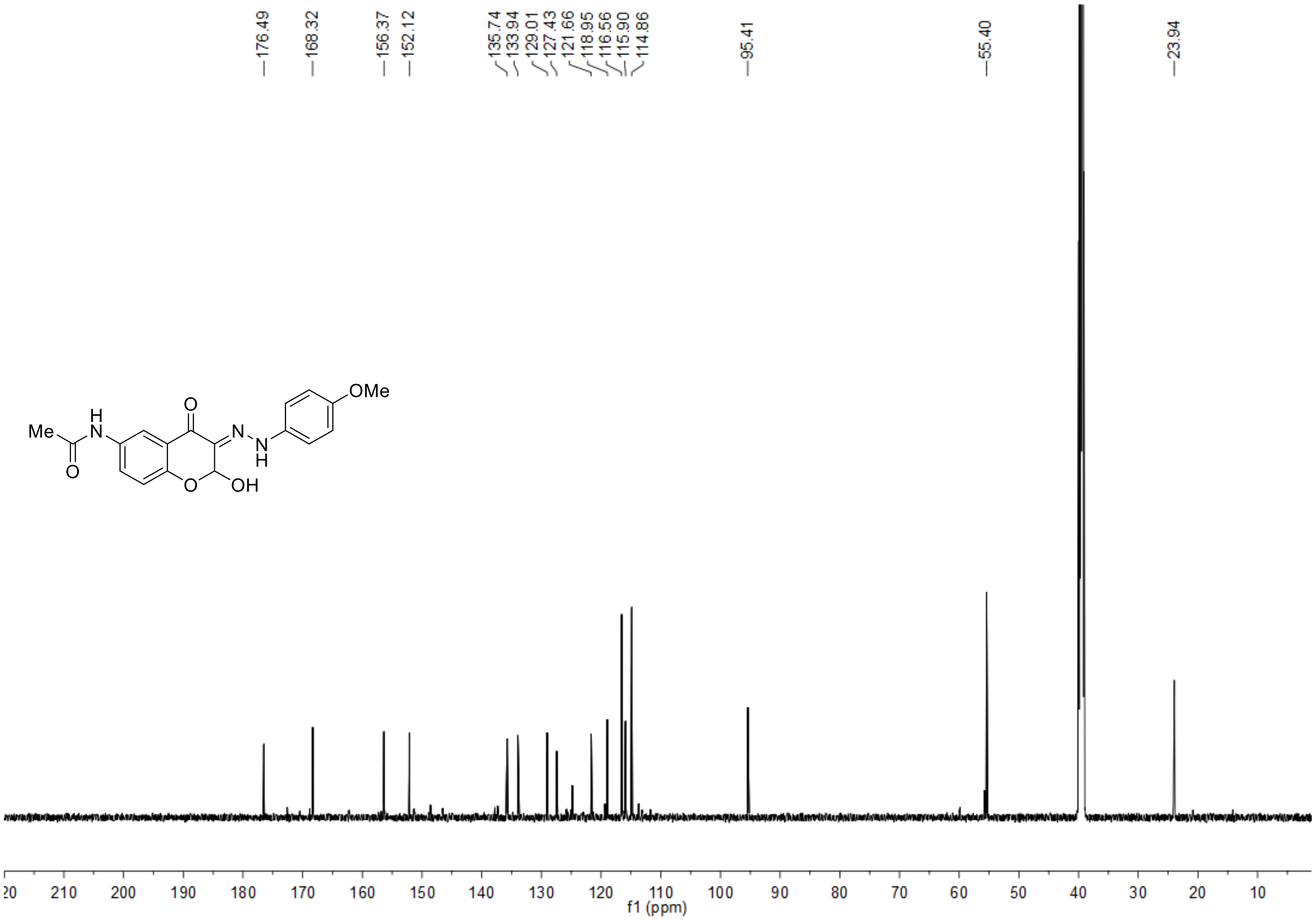

Figure S20. ^{13}C NMR (150 MHz, DMSO-d_6) spectra of compound **3i**

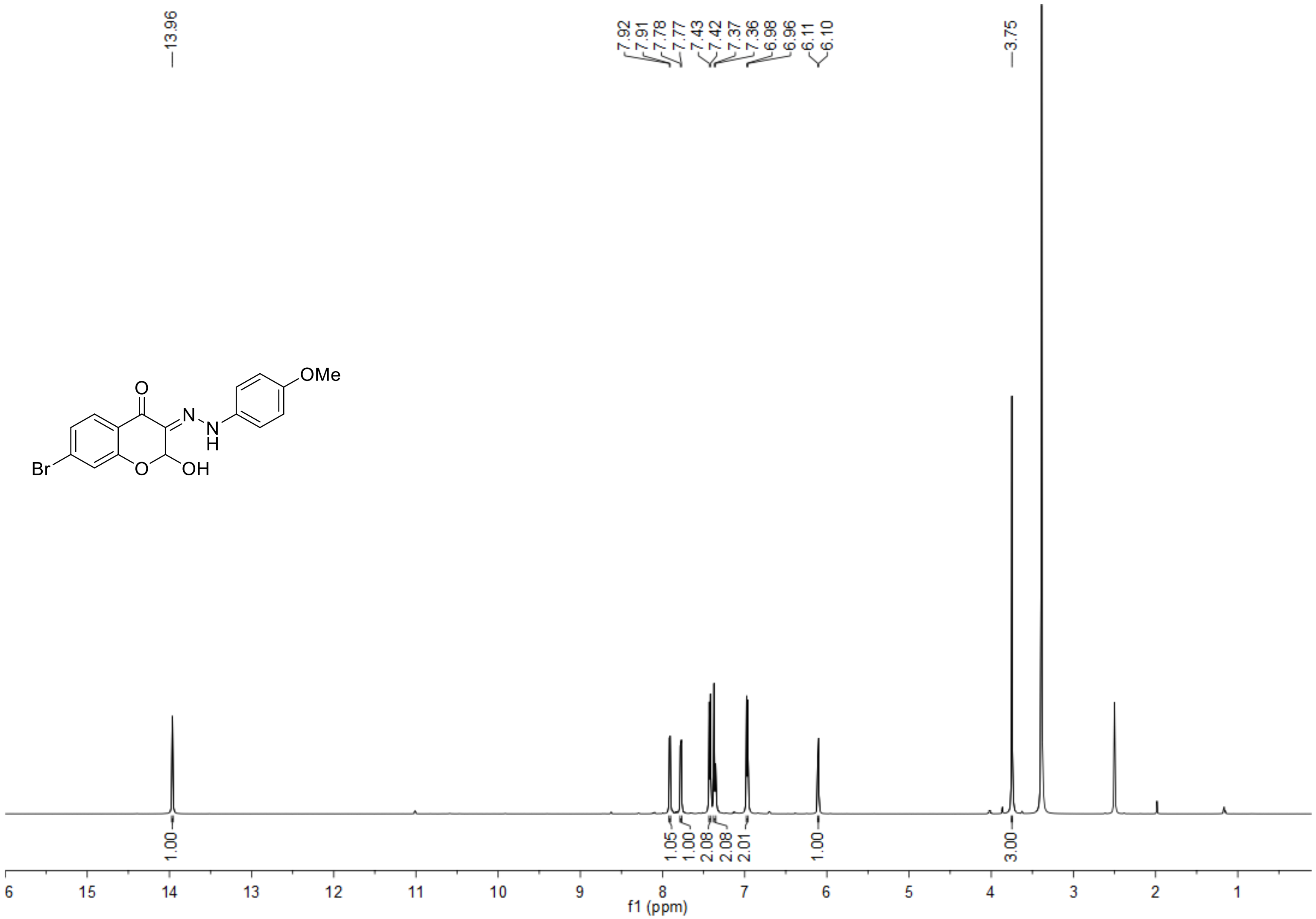

Figure S21. ^{1}H NMR (600 MHz, DMSO-d_6) spectra of compound **3j**

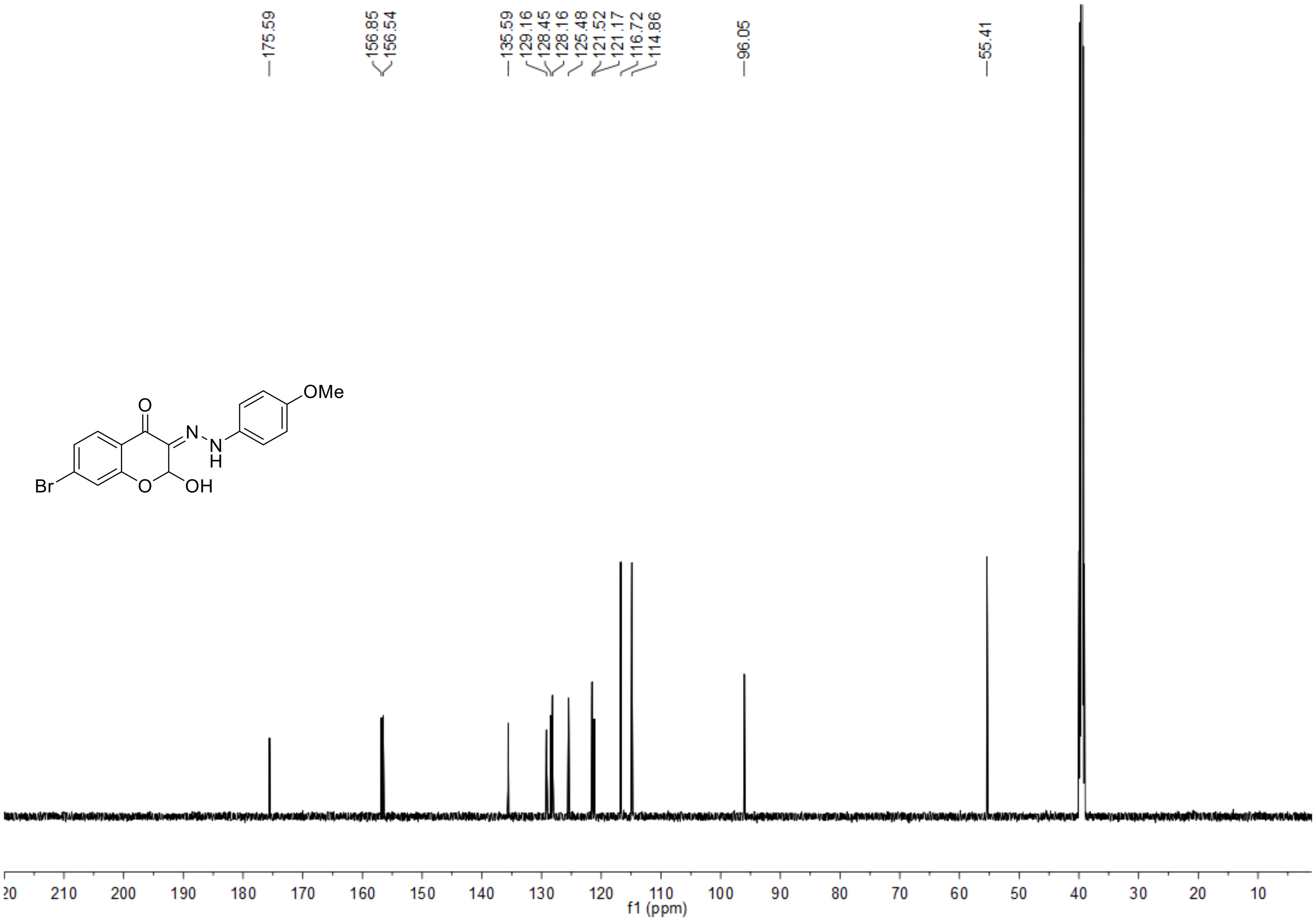

Figure S22. ^{13}C NMR (150 MHz, DMSO-d_6) spectra of compound **3j**

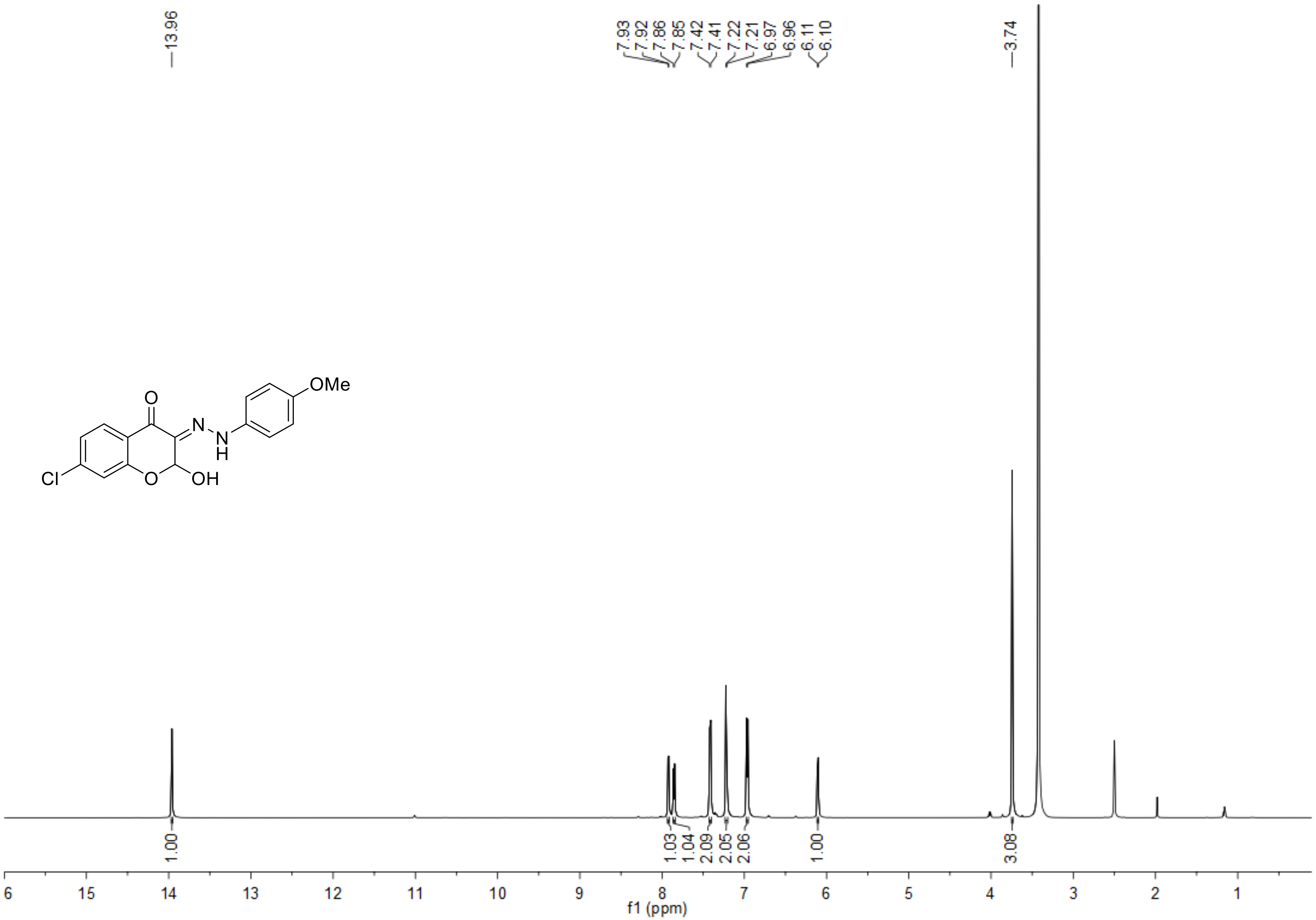

Figure S23. ^{1}H NMR (600 MHz, DMSO-d_6) spectra of compound **3k**

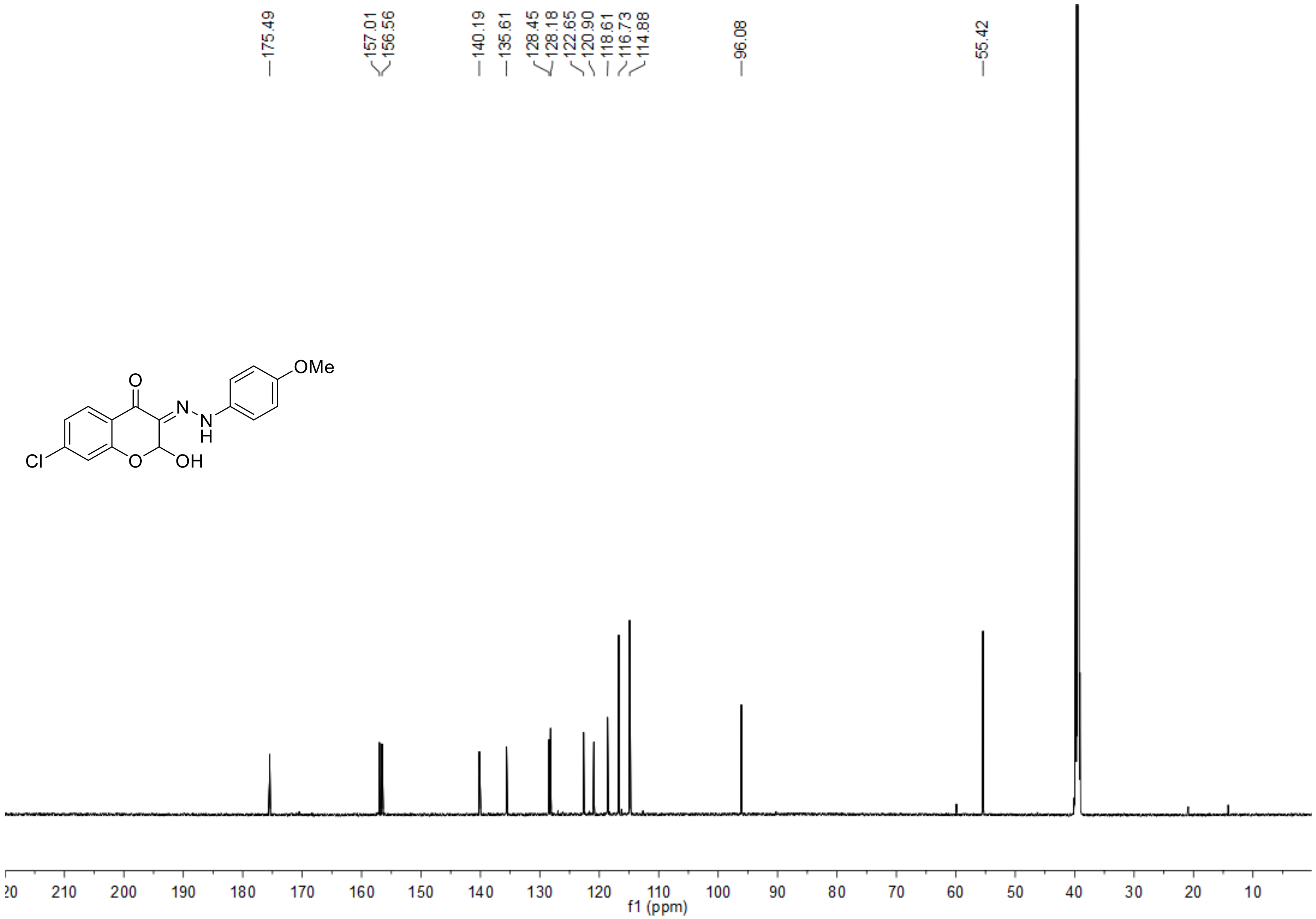

Figure S24. ^{13}C NMR (150 MHz, DMSO-d_6) spectra of compound **3k**

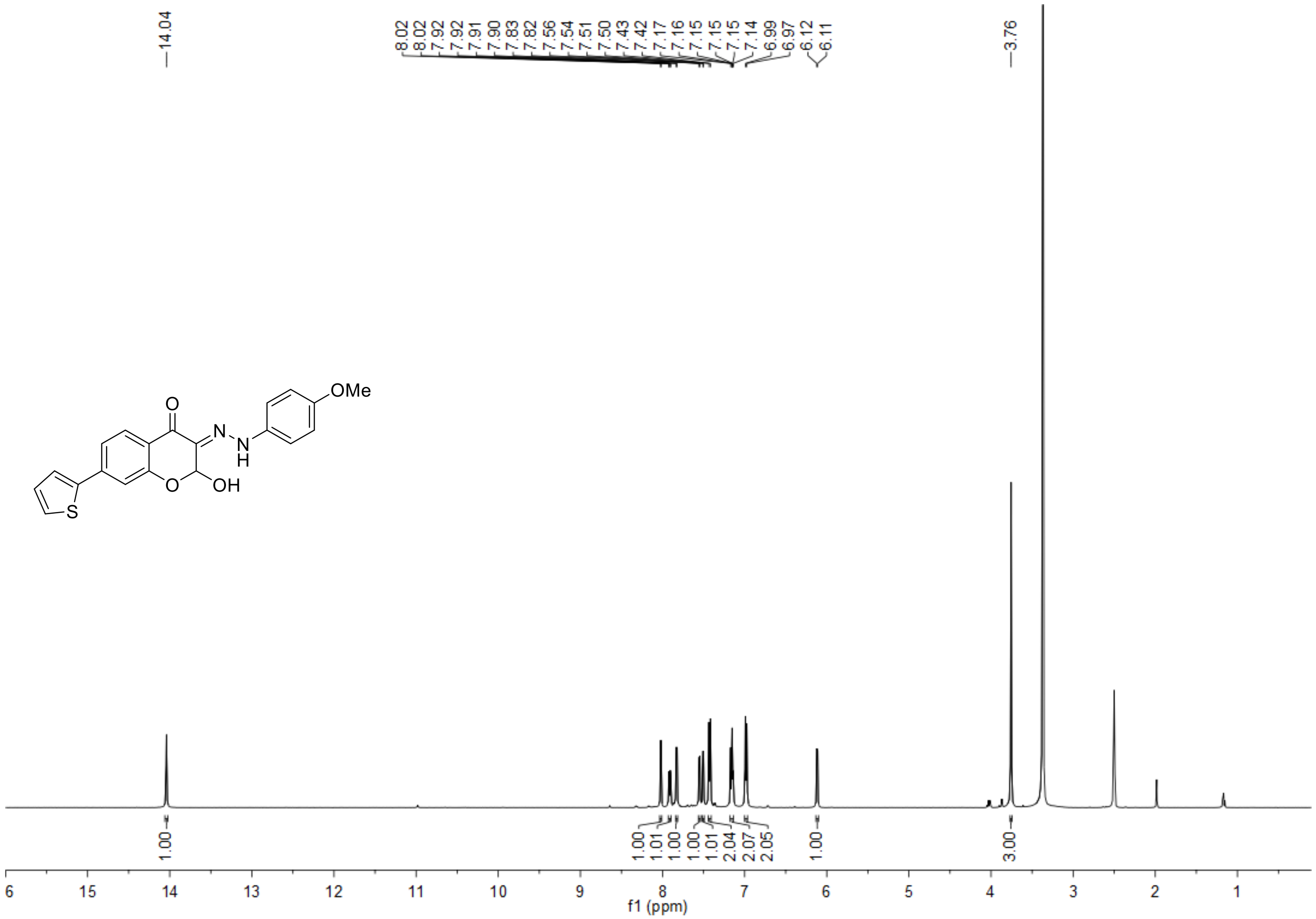

Figure S25. ^{1}H NMR (500 MHz, DMSO-d_6) spectra of compound **3l**

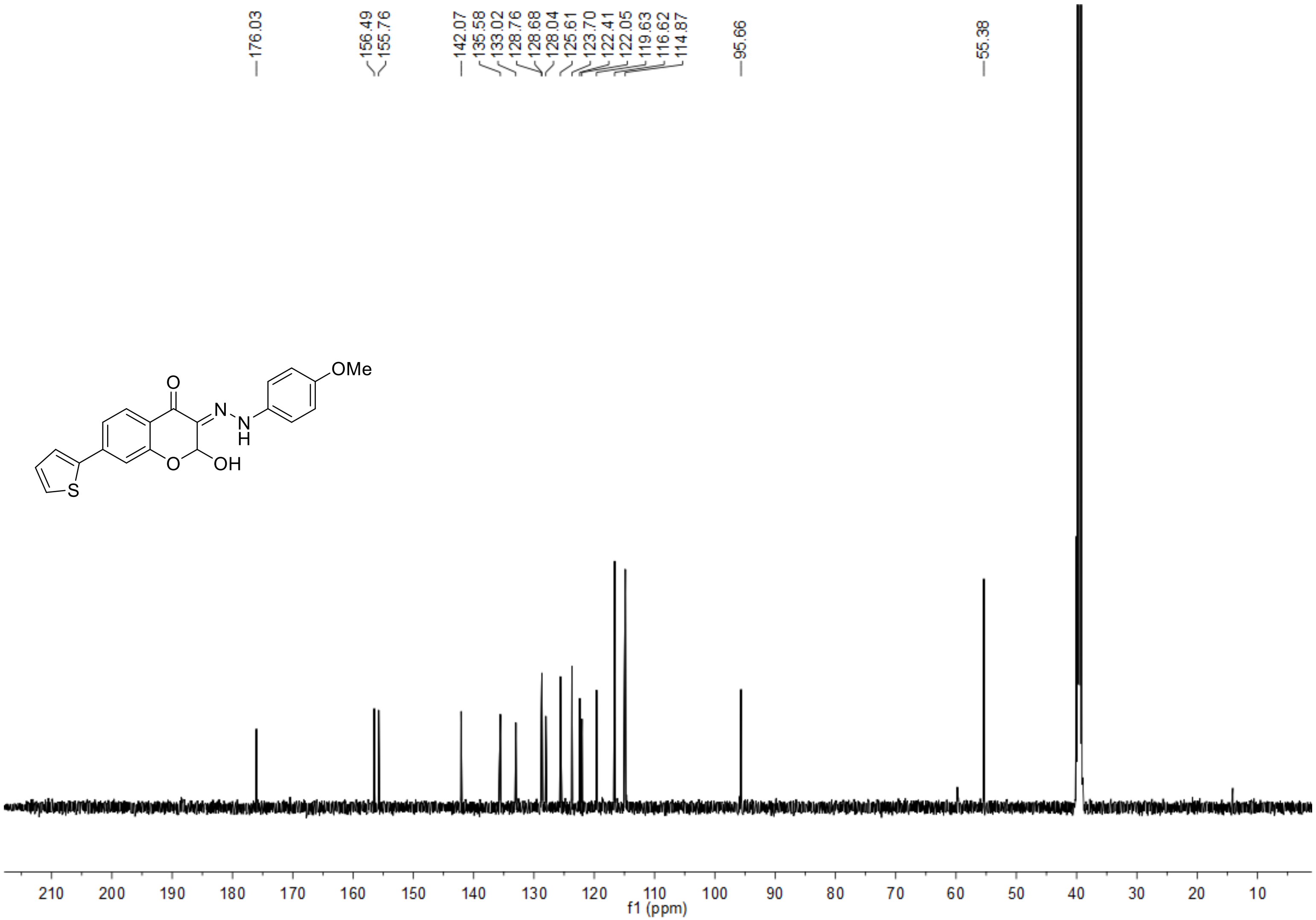

Figure S26. ^{13}C NMR (125 MHz, DMSO-d_6) spectra of compound **3l**

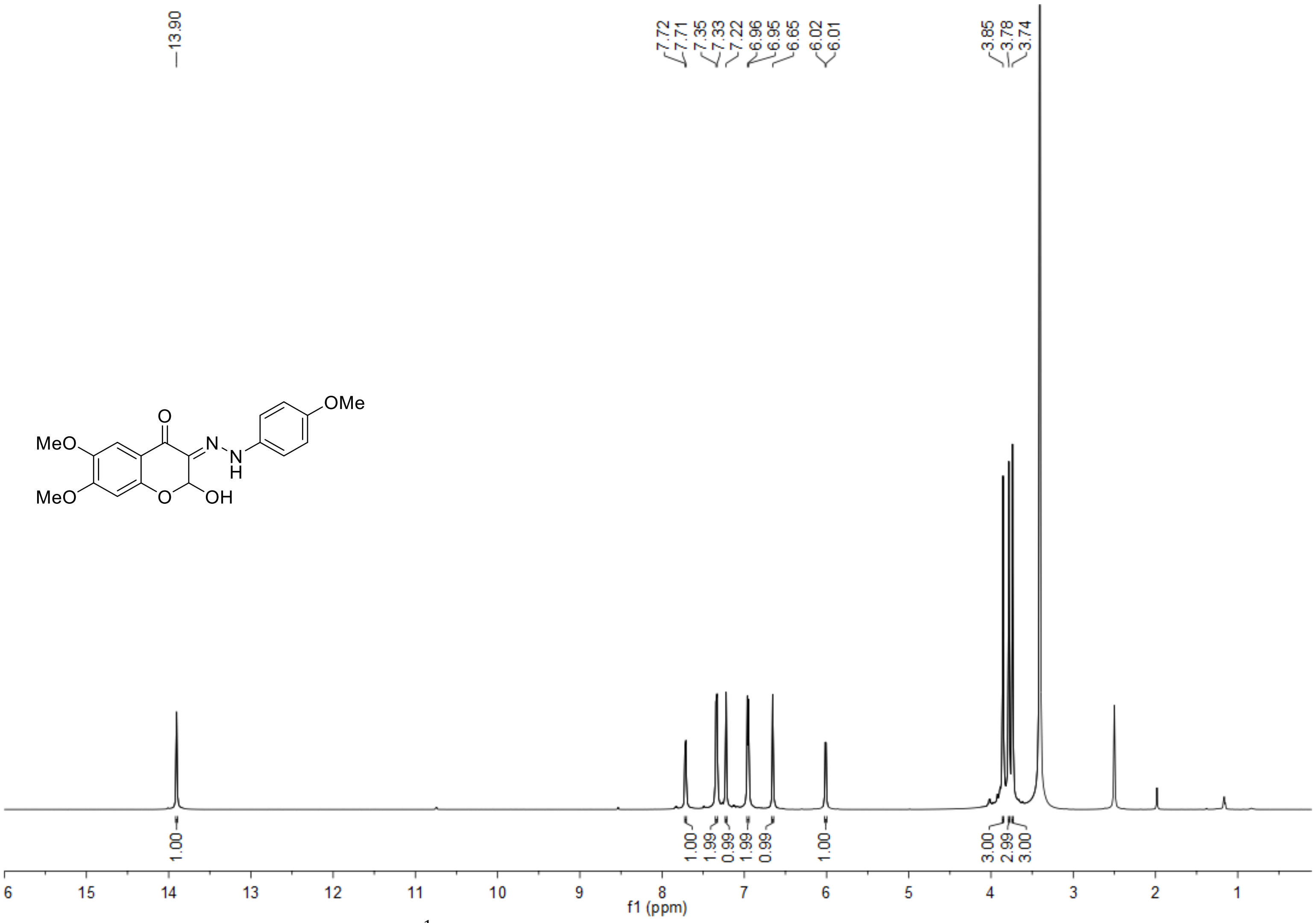

Figure S27. ^{1}H NMR (600 MHz, DMSO-d_6) spectra of compound **3m**

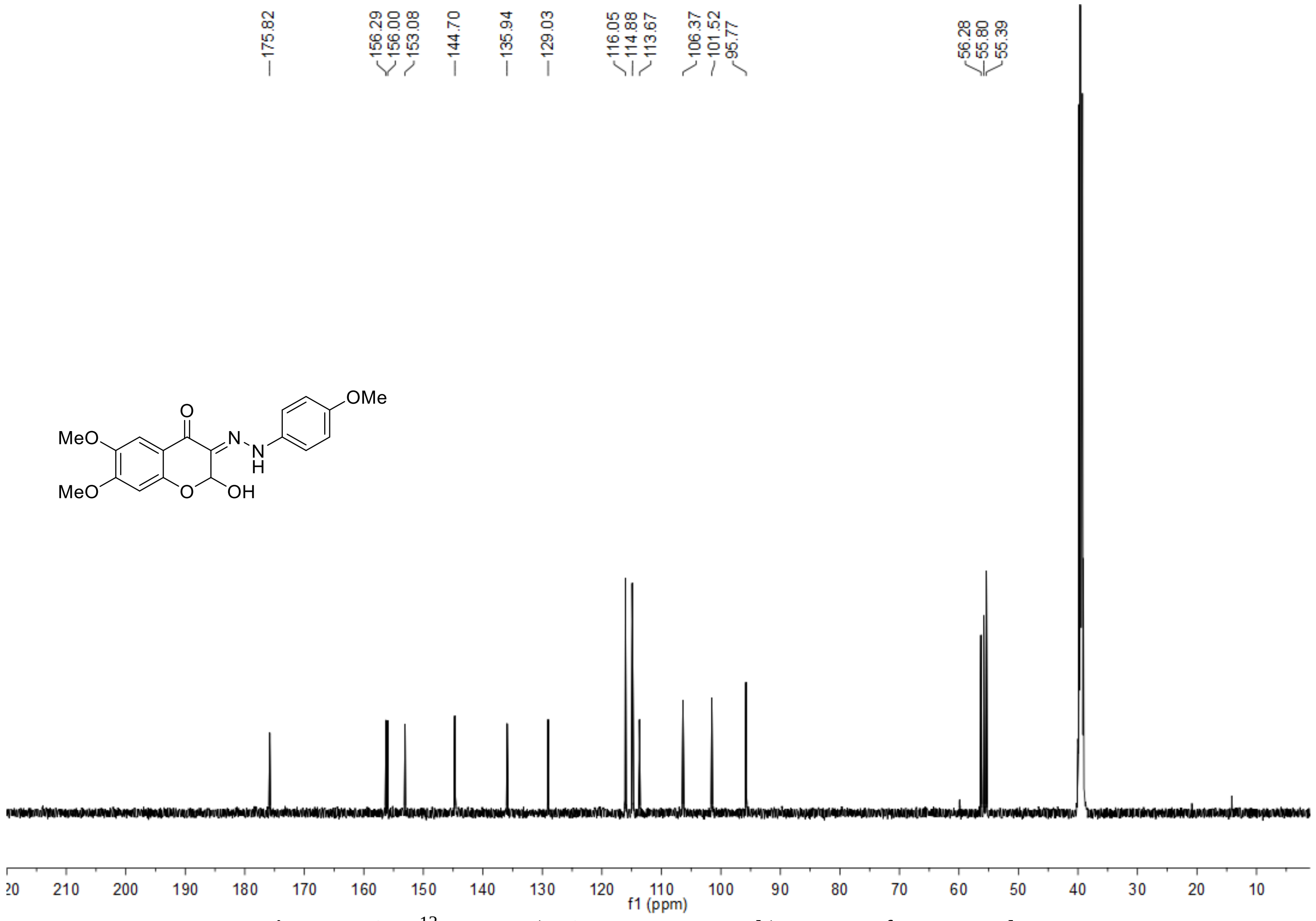

Figure S28. ^{13}C NMR (150 MHz, DMSO-d_6) spectra of compound **3m**

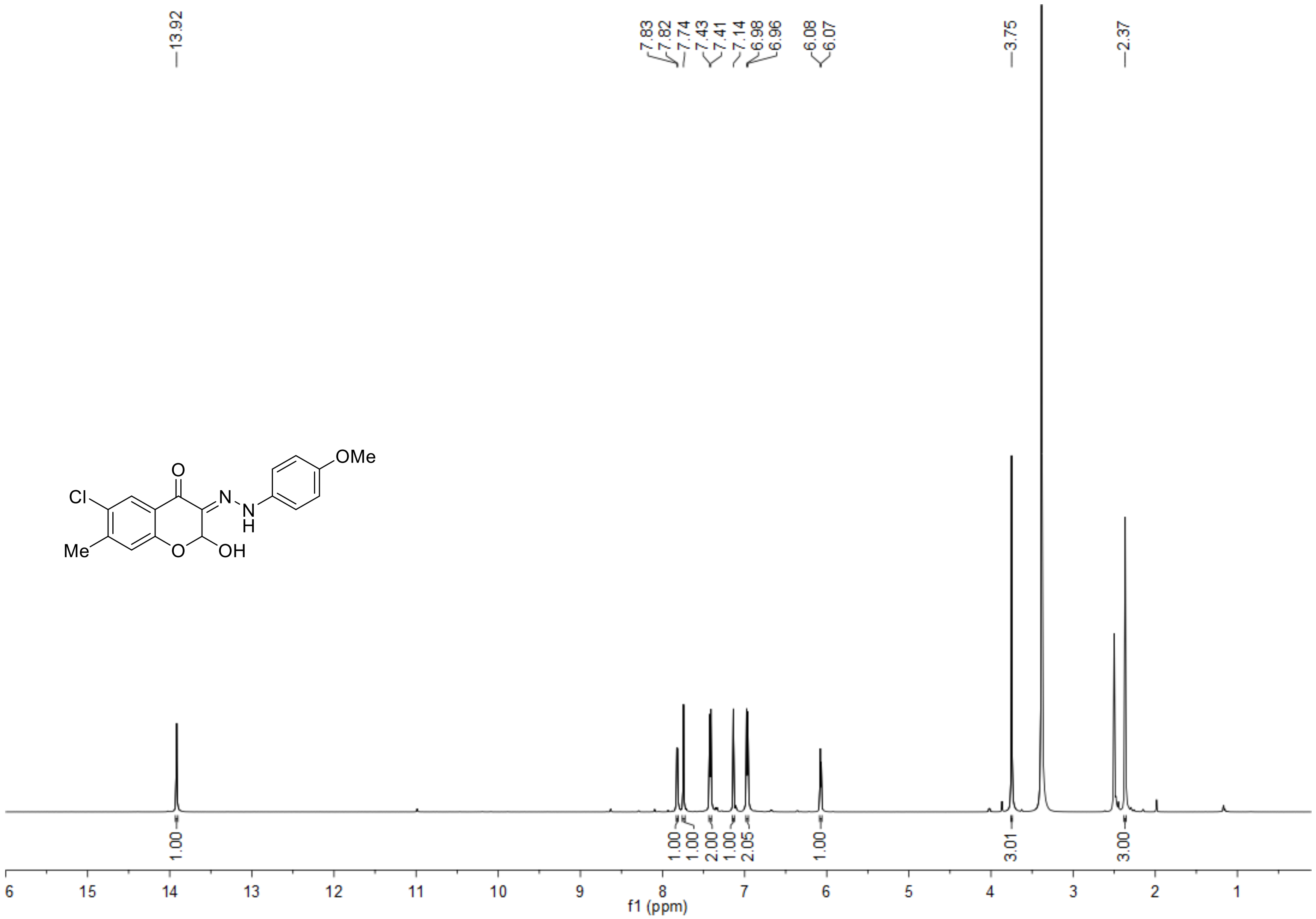

Figure S29. ^{1}H NMR (600 MHz, DMSO-d_6) spectra of compound **3n**

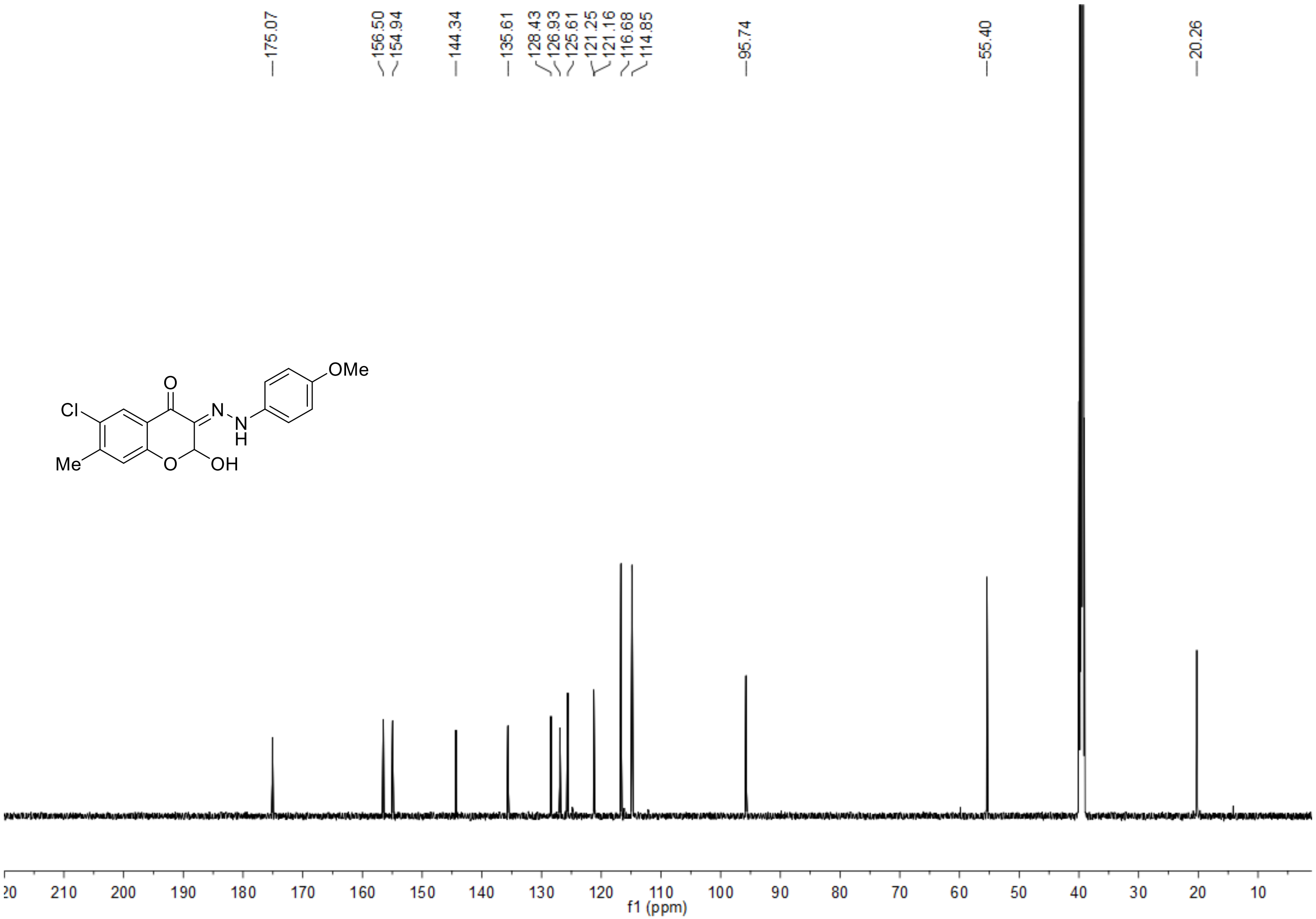

Figure S30. ^{13}C NMR (150 MHz, DMSO-d_6) spectra of compound **3n**

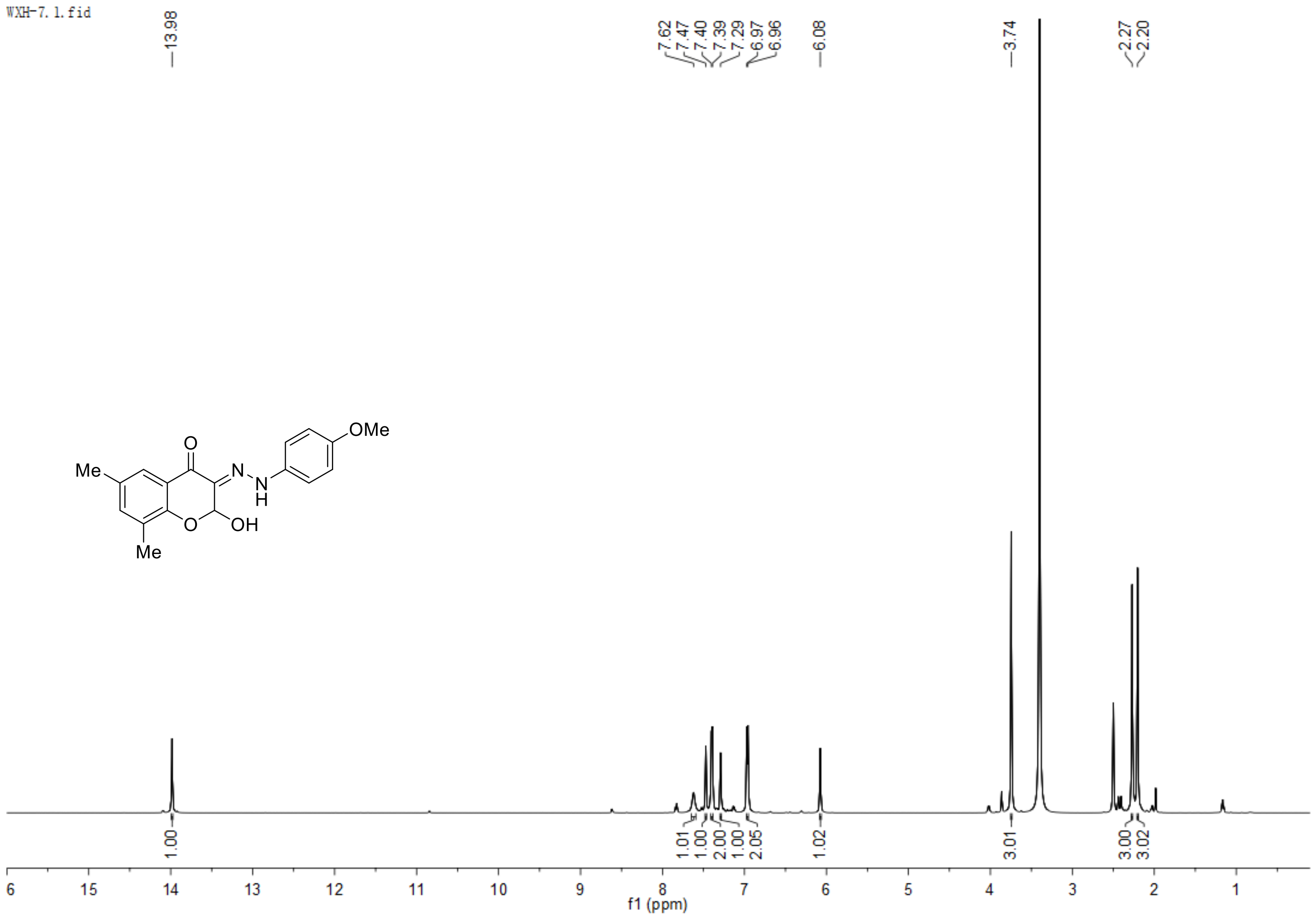

Figure S31. ^{1}H NMR (600 MHz, DMSO-d_6) spectra of compound **3o**

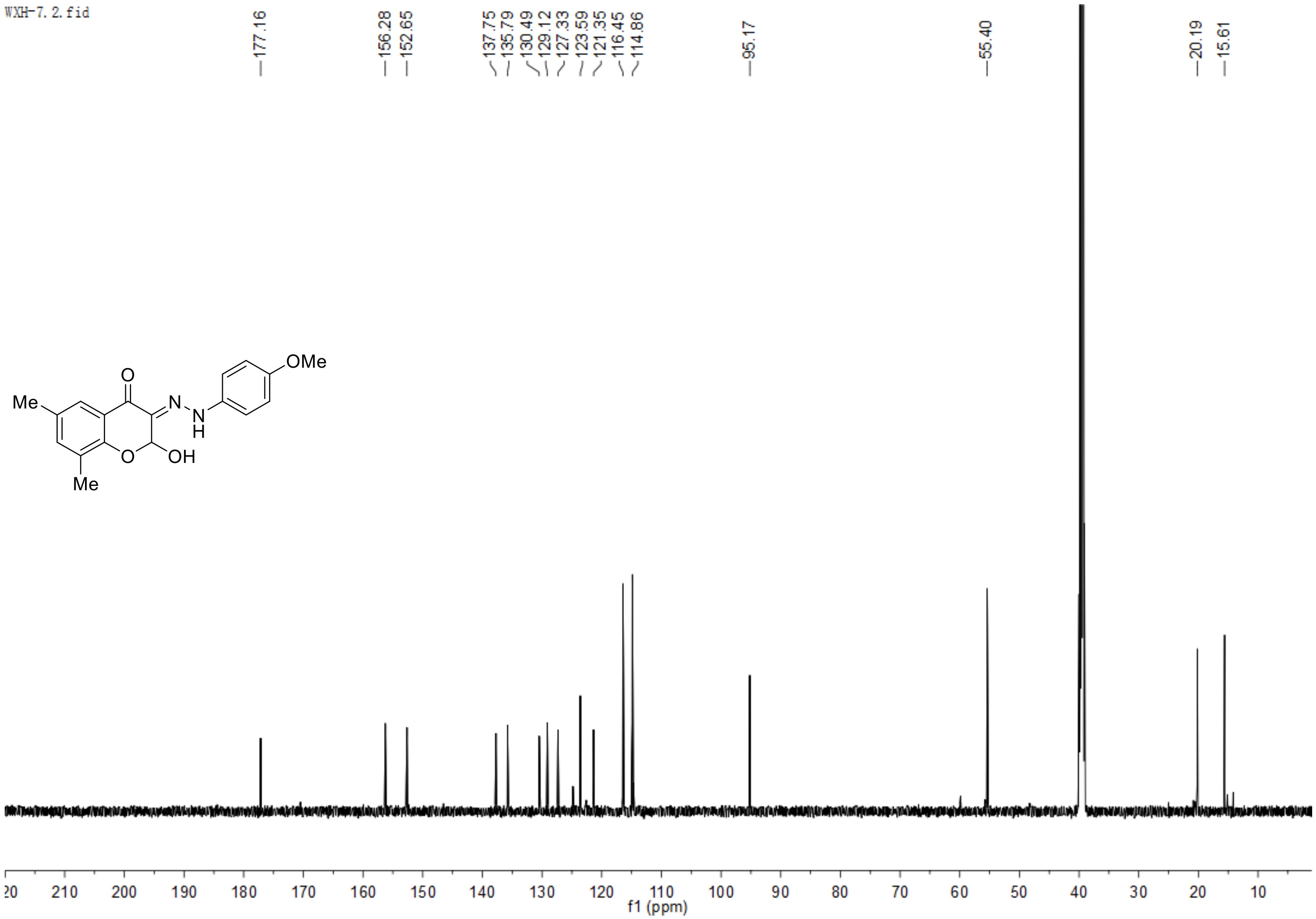

Figure S32. ^{13}C NMR (150 MHz, DMSO-d_6) spectra of compound **3o**

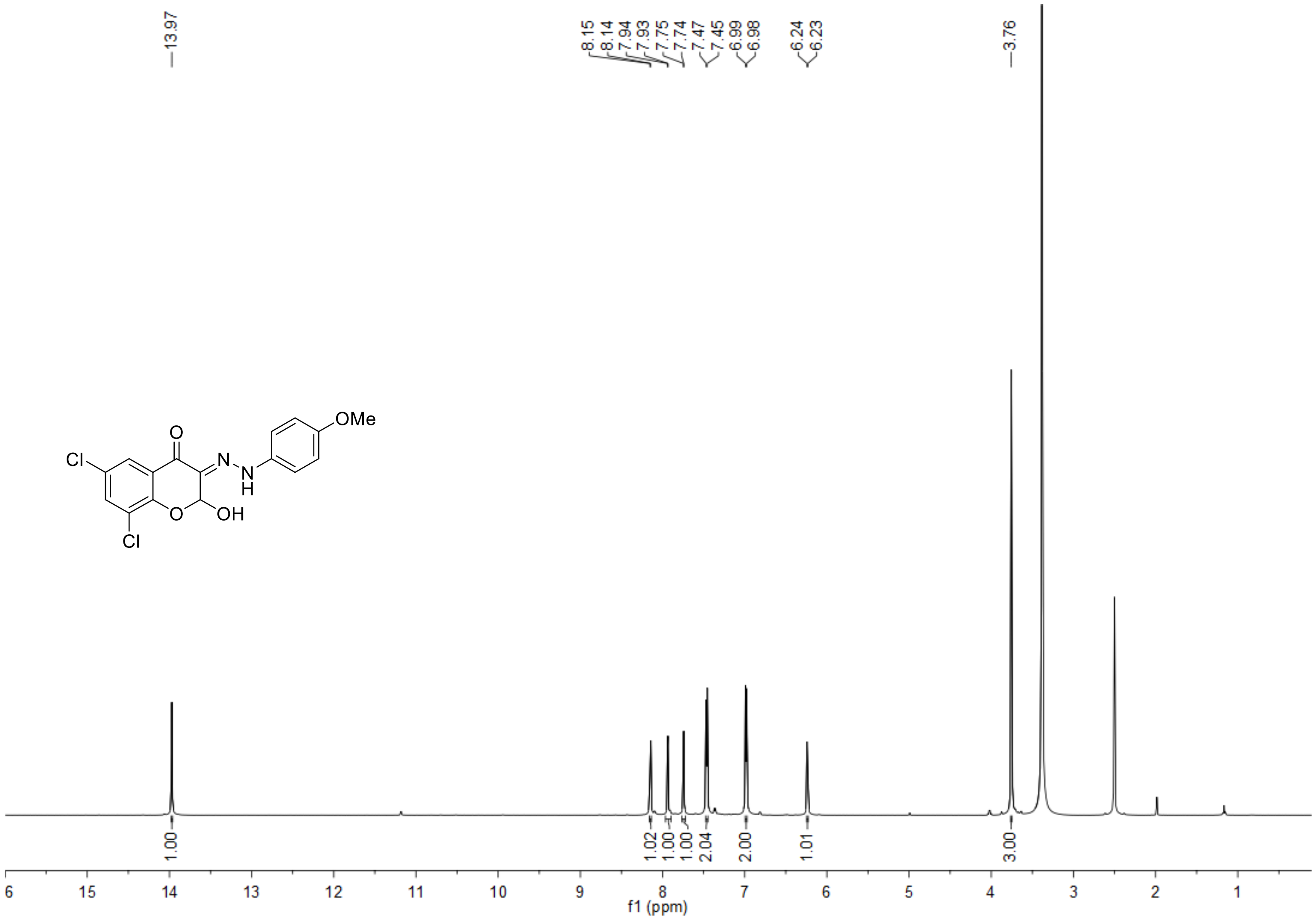

Figure S33. ^{1}H NMR (600 MHz, DMSO-d_6) spectra of compound **3p**

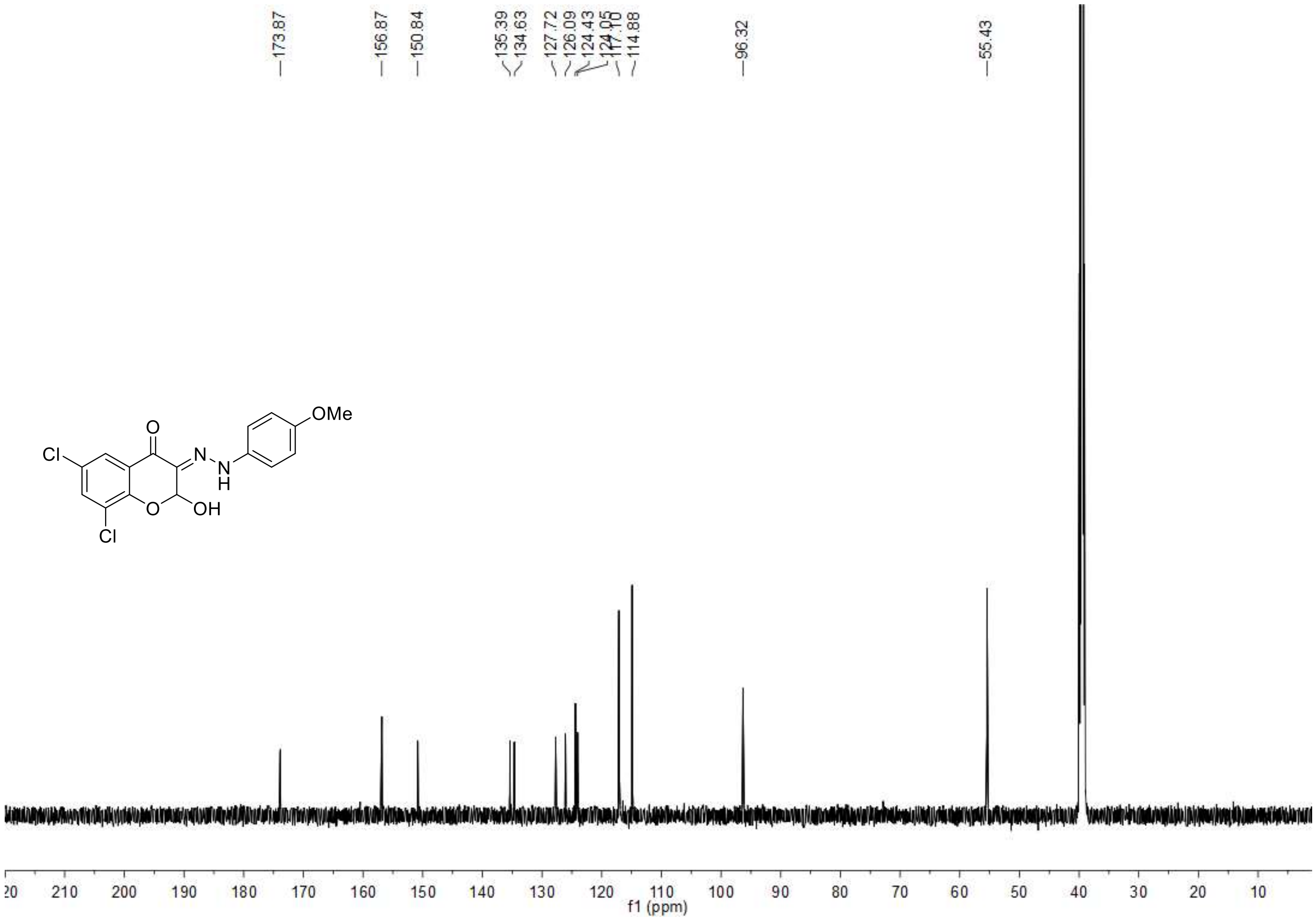

Figure S34. ^{13}C NMR (150 MHz, DMSO-d_6) spectra of compound **3p**

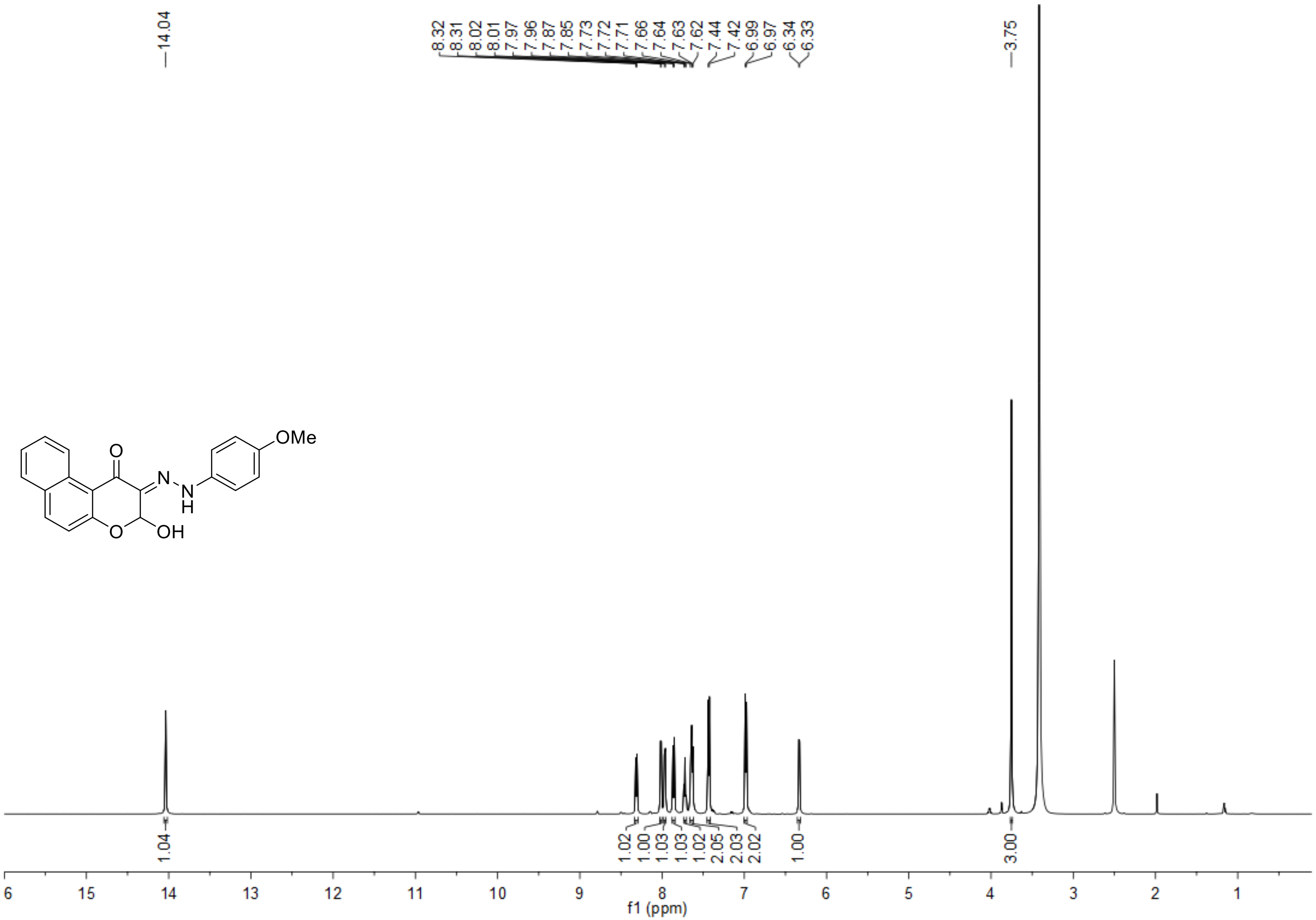

Figure S35. ^{1}H NMR (600 MHz, DMSO-d_6) spectra of compound **3q**

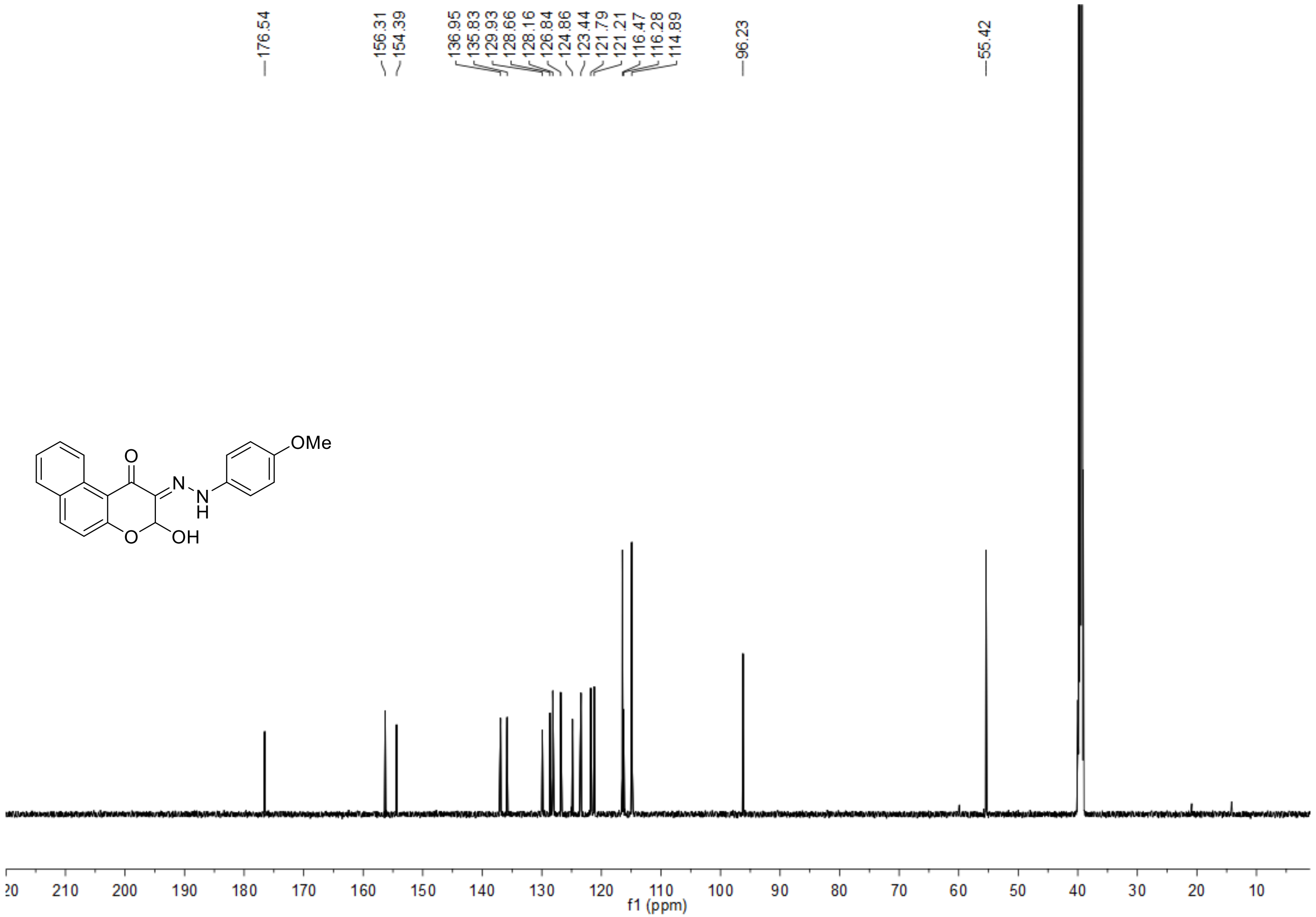

Figure S36. ^{13}C NMR (150 MHz, DMSO-d_6) spectra of compound **3q**

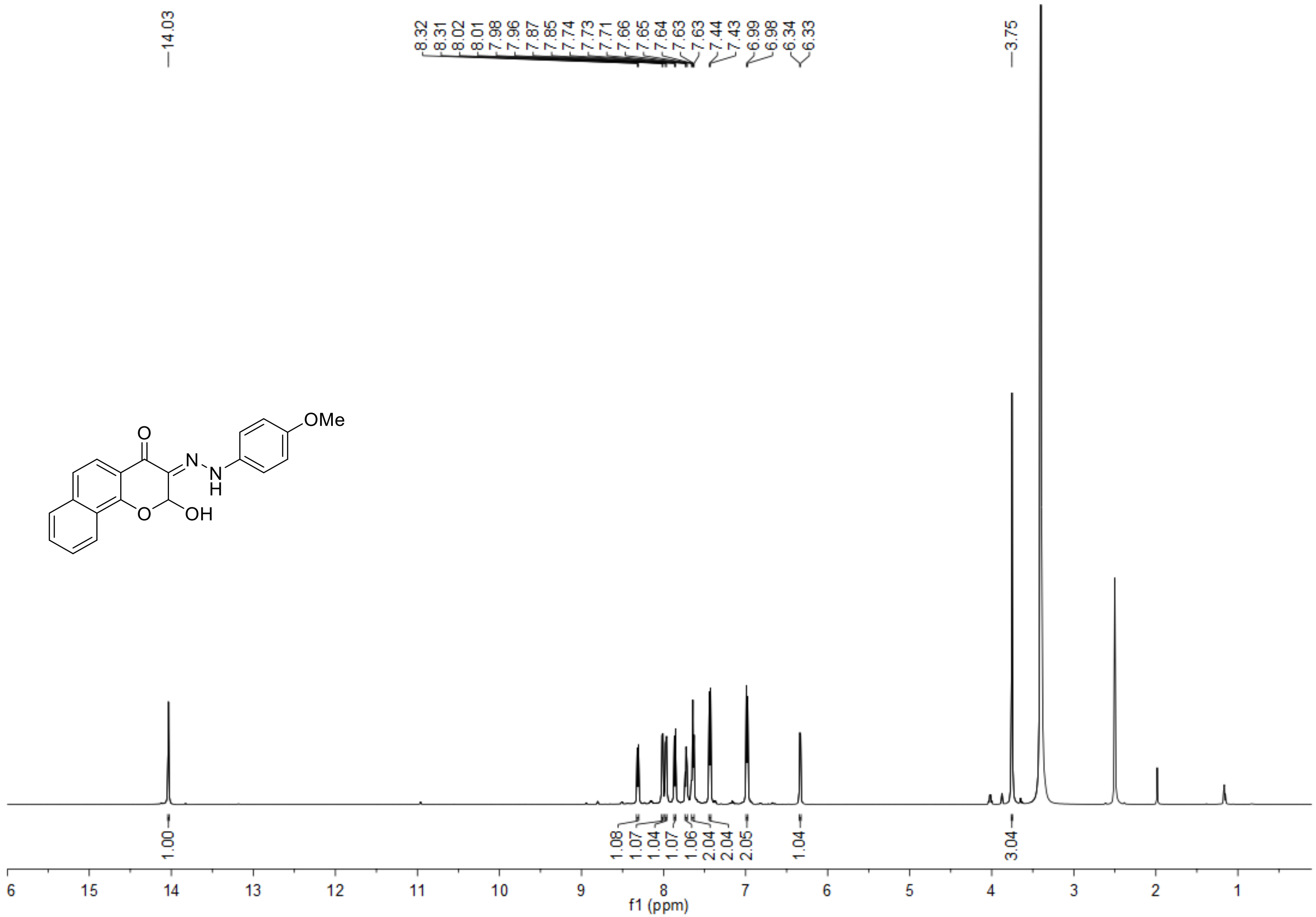

Figure S37. ^{1}H NMR (600 MHz, DMSO-d_6) spectra of compound **3r**

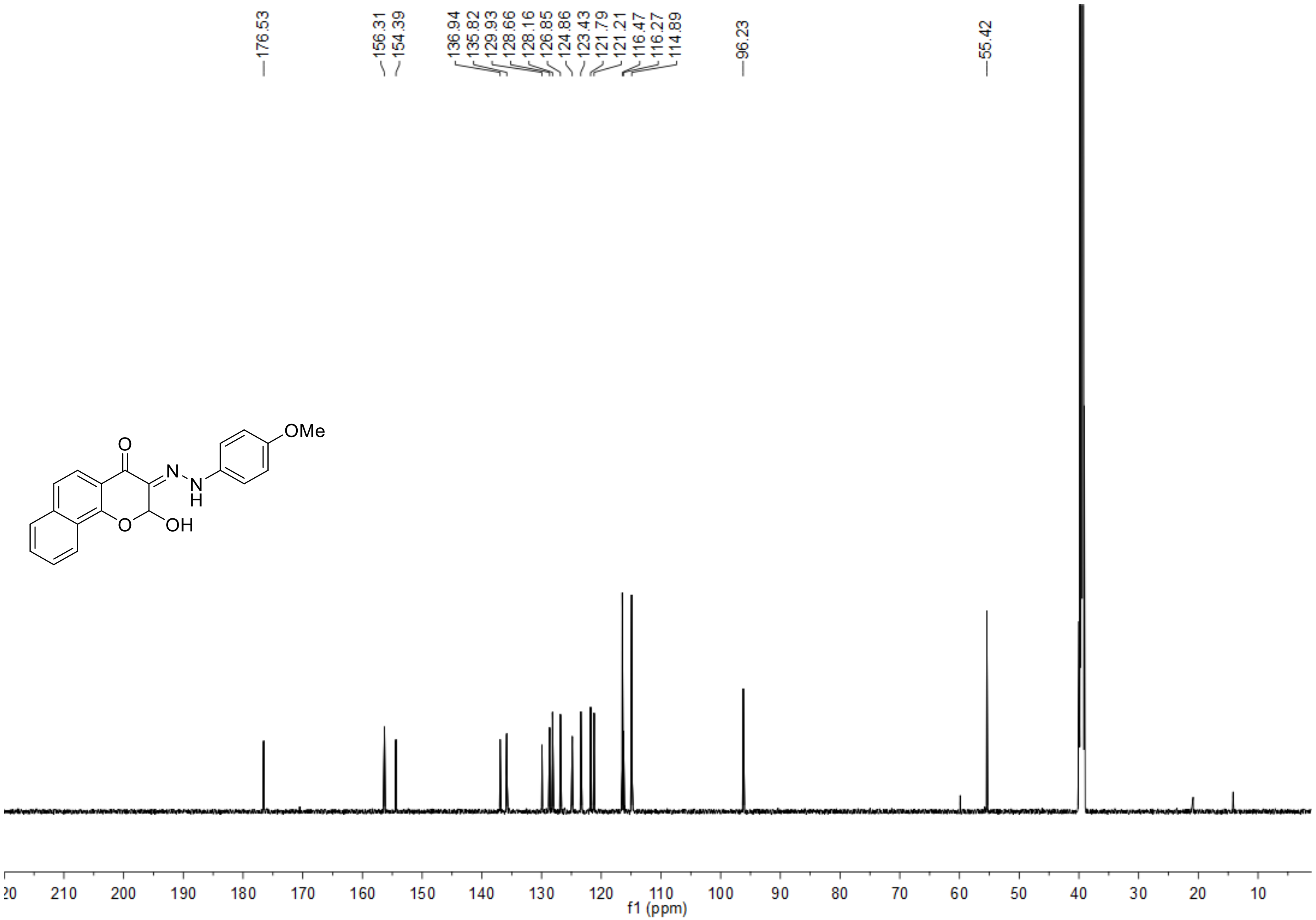

Figure S38. ^{13}C NMR (150 MHz, DMSO-d_6) spectra of compound **3r**

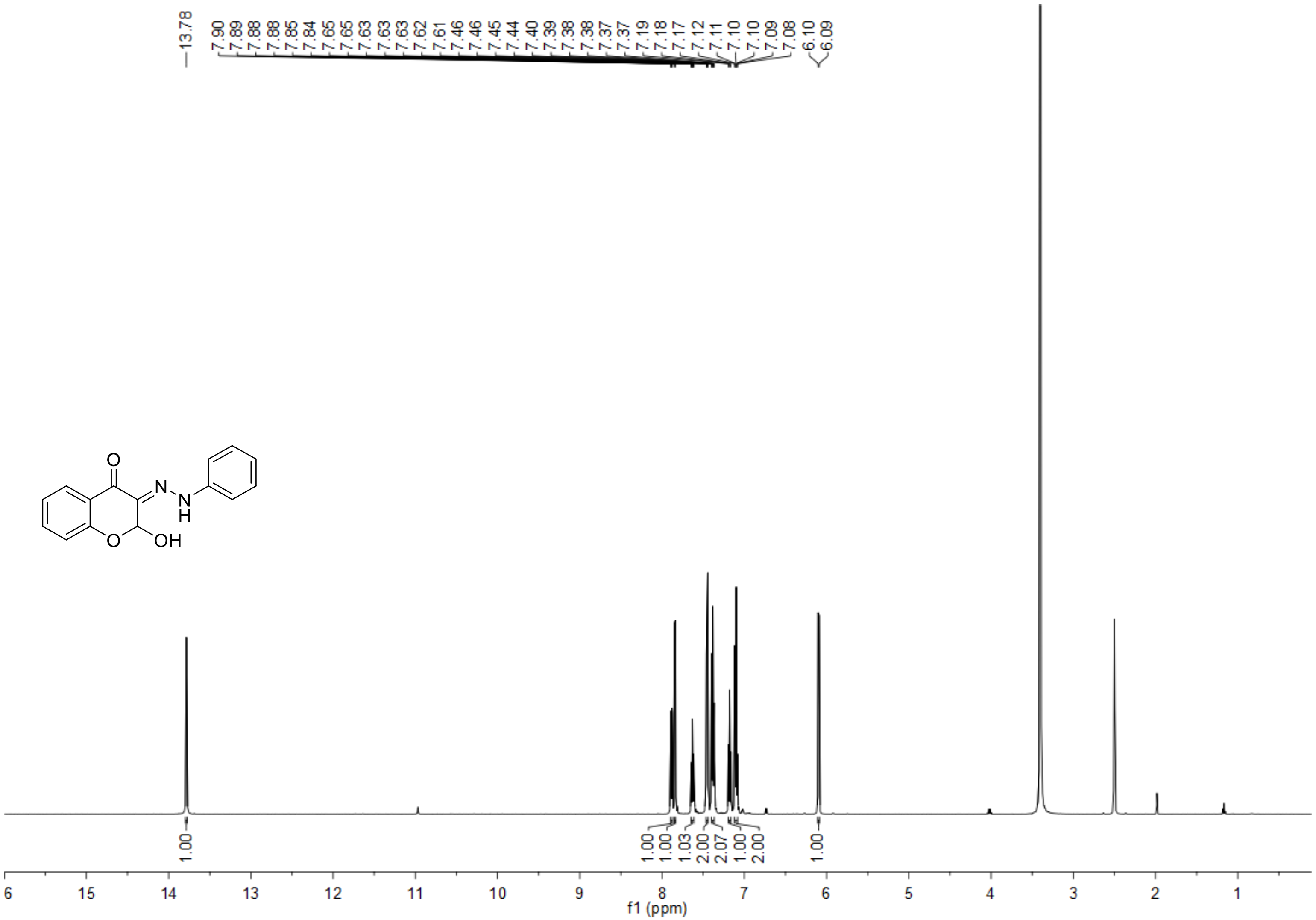

Figure S39. ^{1}H NMR (500 MHz, DMSO-d_6) spectra of compound **3s**

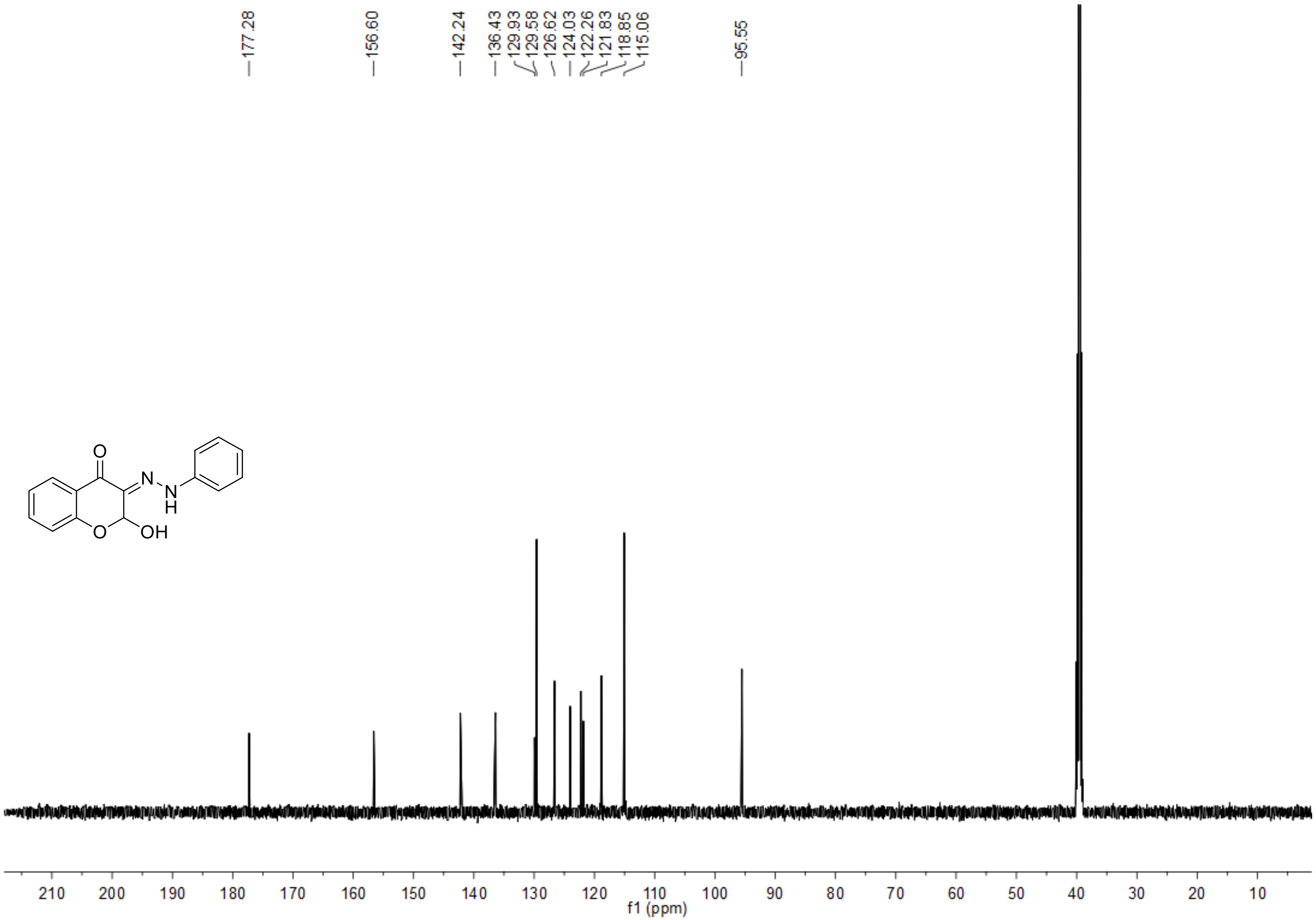

Figure S40. ^{13}C NMR (125 MHz, DMSO-d_6) spectra of compound **3s**

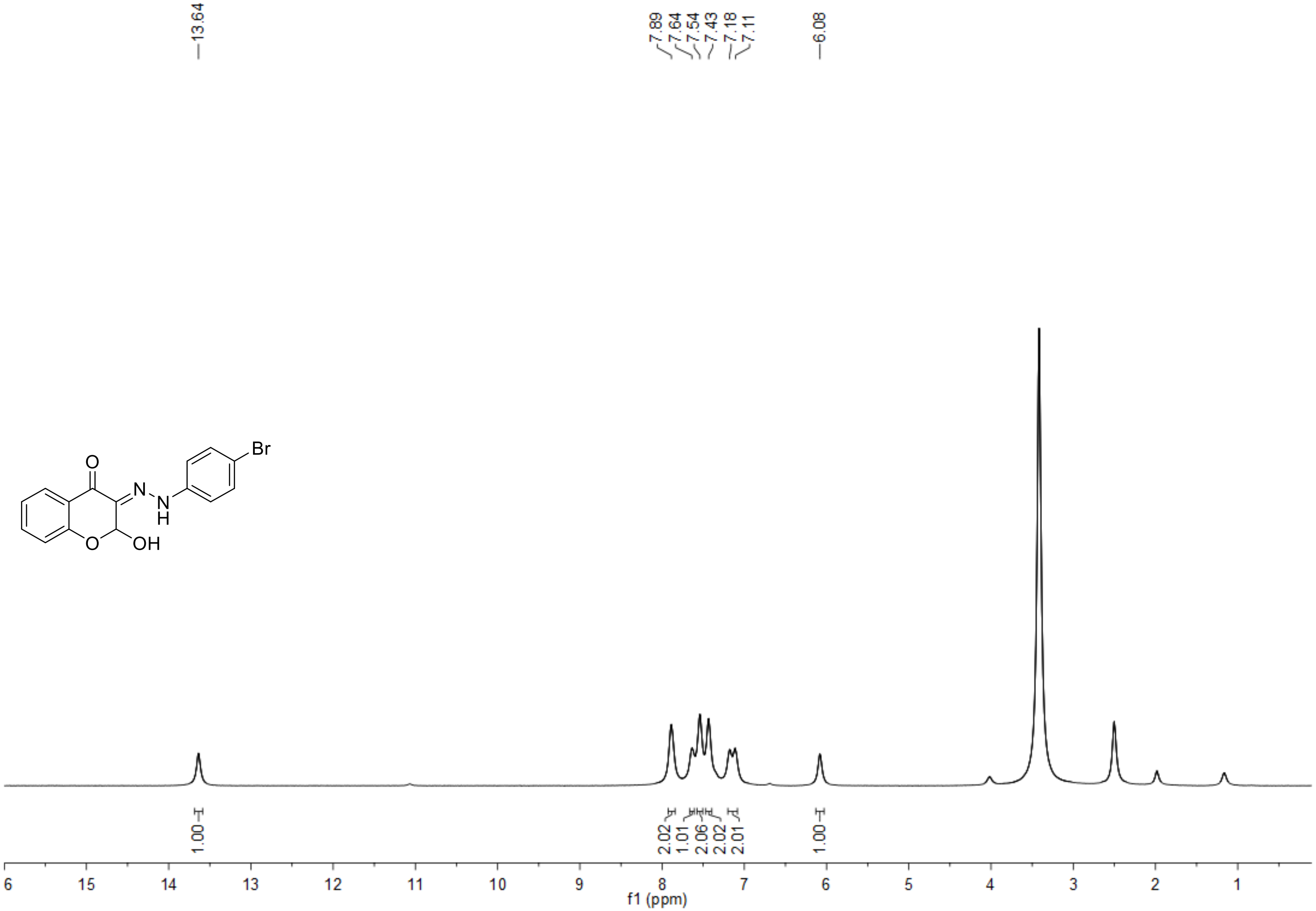

Figure S41. ^{1}H NMR (500 MHz, DMSO-d_6) spectra of compound **3t**

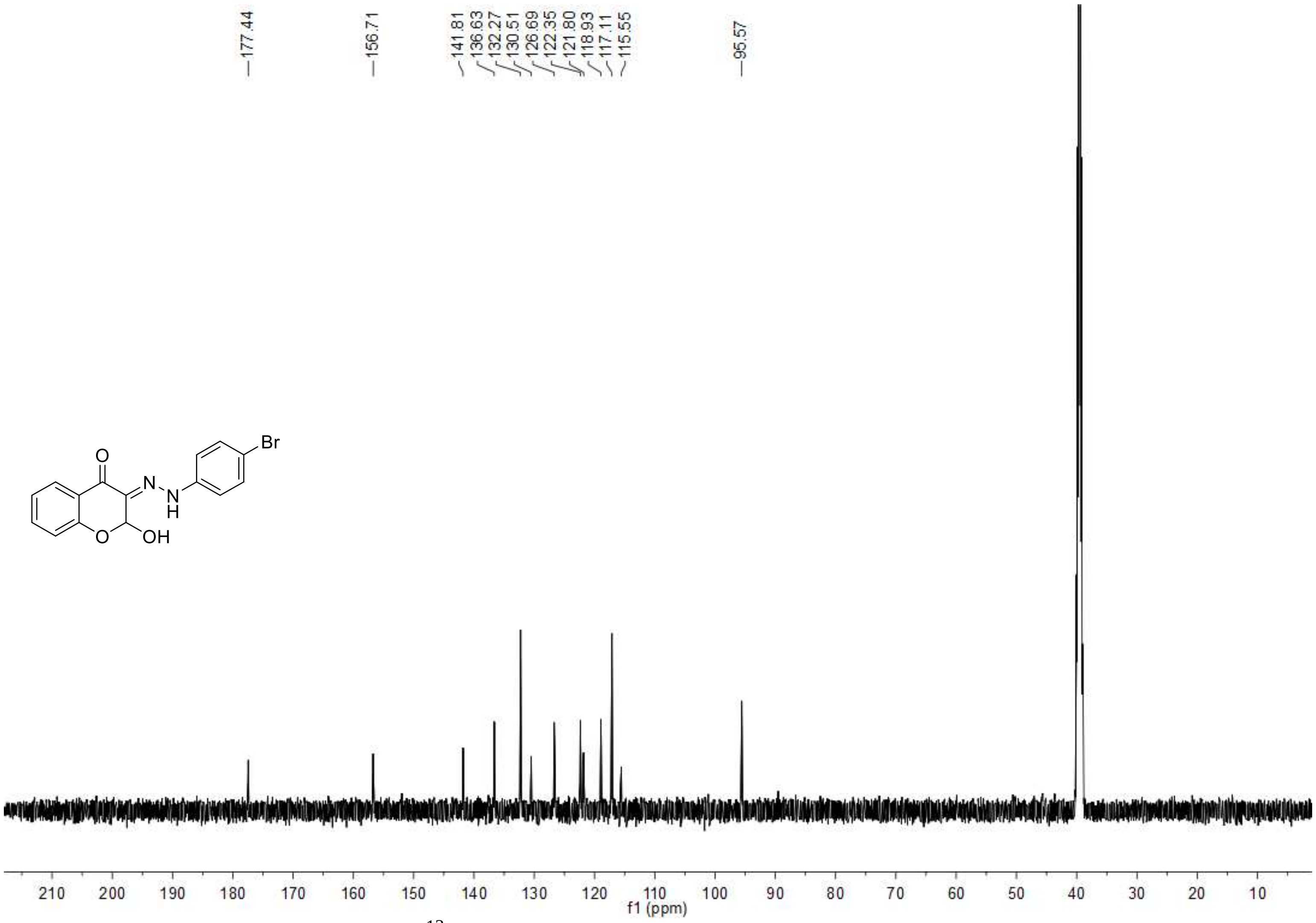

Figure S42. ^{13}C NMR (125 MHz, DMSO-d_6) spectra of compound **3t**

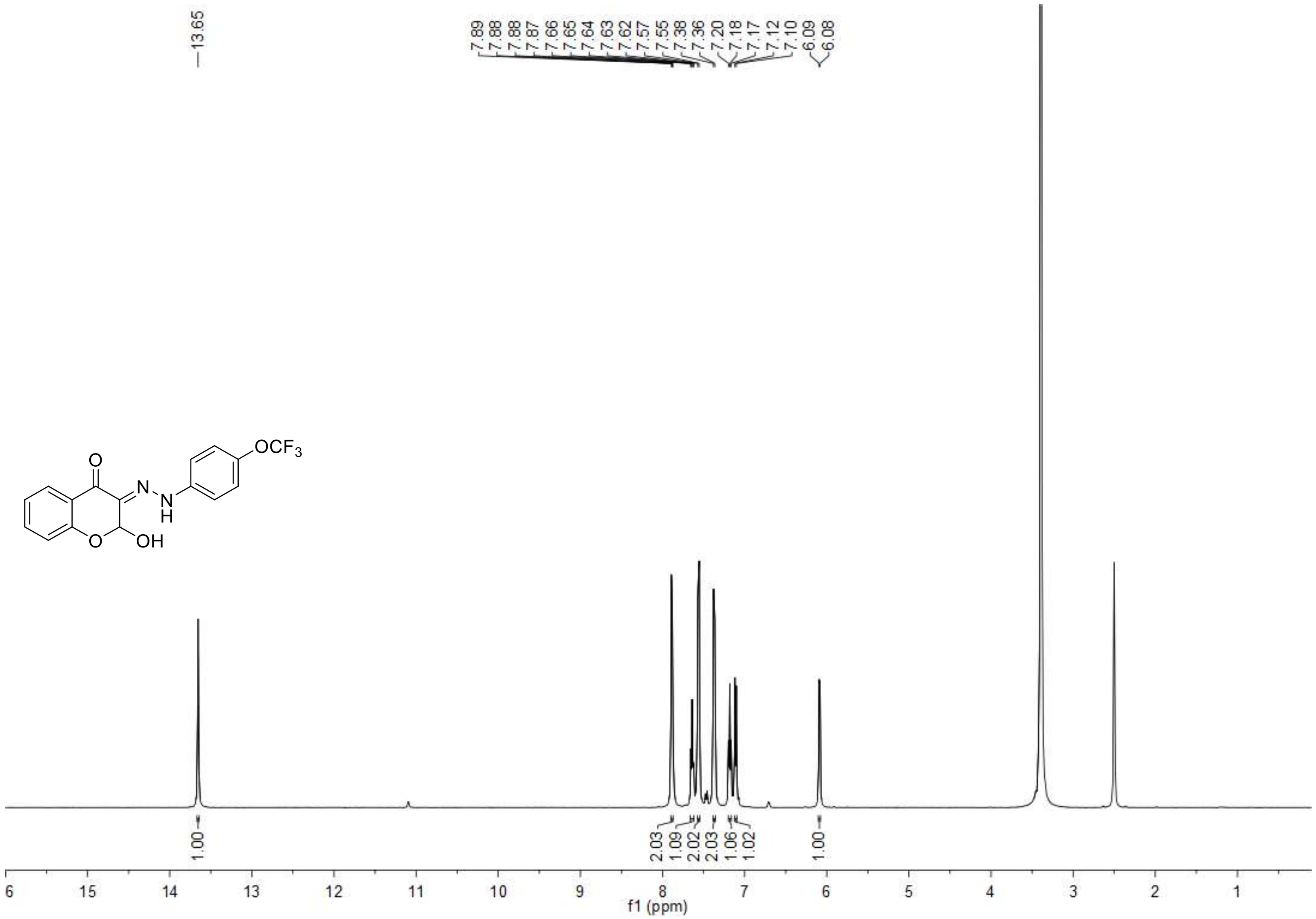

Figure S43. ^{1}H NMR (500 MHz, DMSO-d_6) spectra of compound **3u**

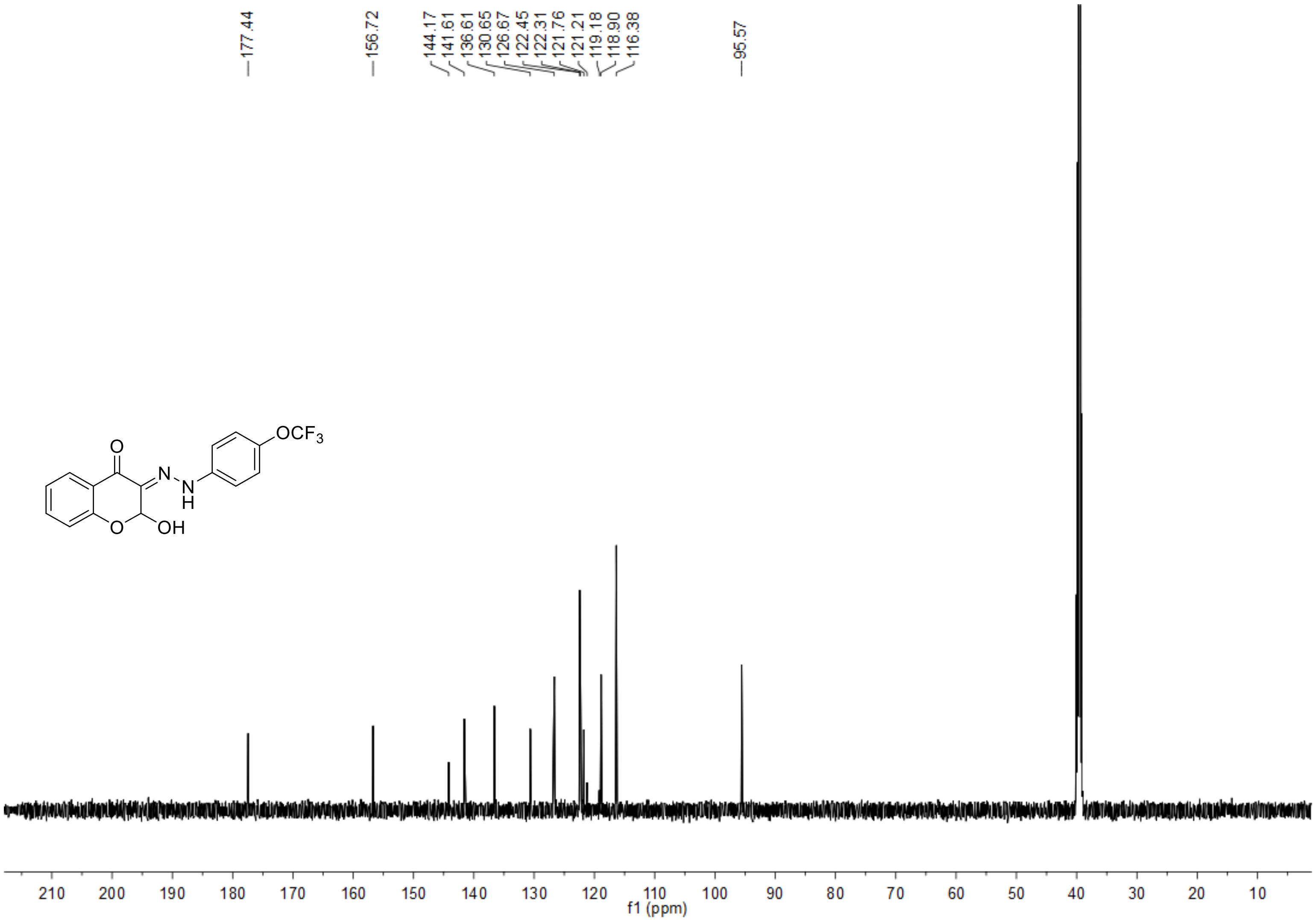

Figure S44. ^{13}C NMR (125 MHz, DMSO-d_6) spectra of compound **3u**

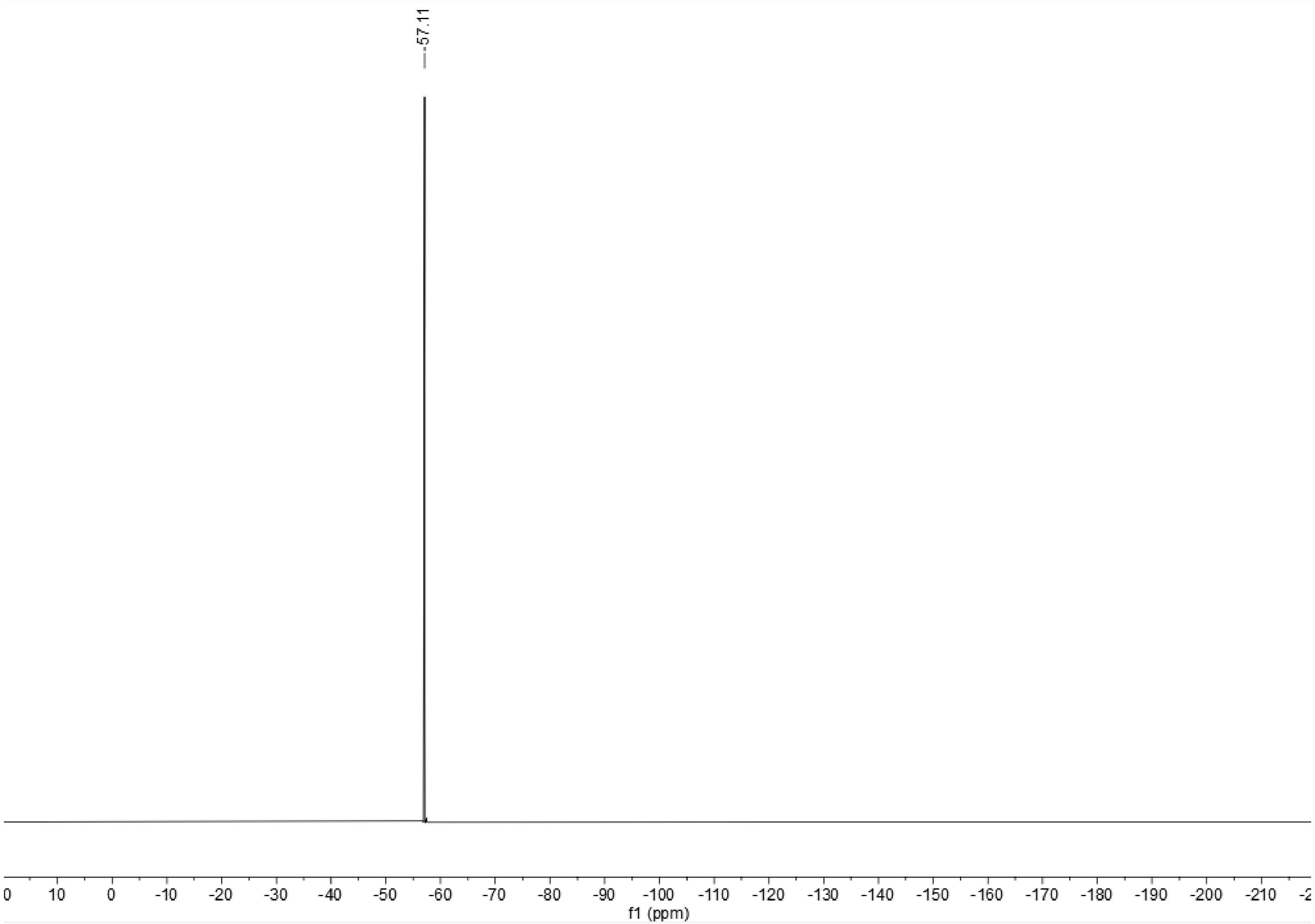

Figure S45. ^{19}F NMR (470 MHz, DMSO-d_6) spectra of compound **3u**

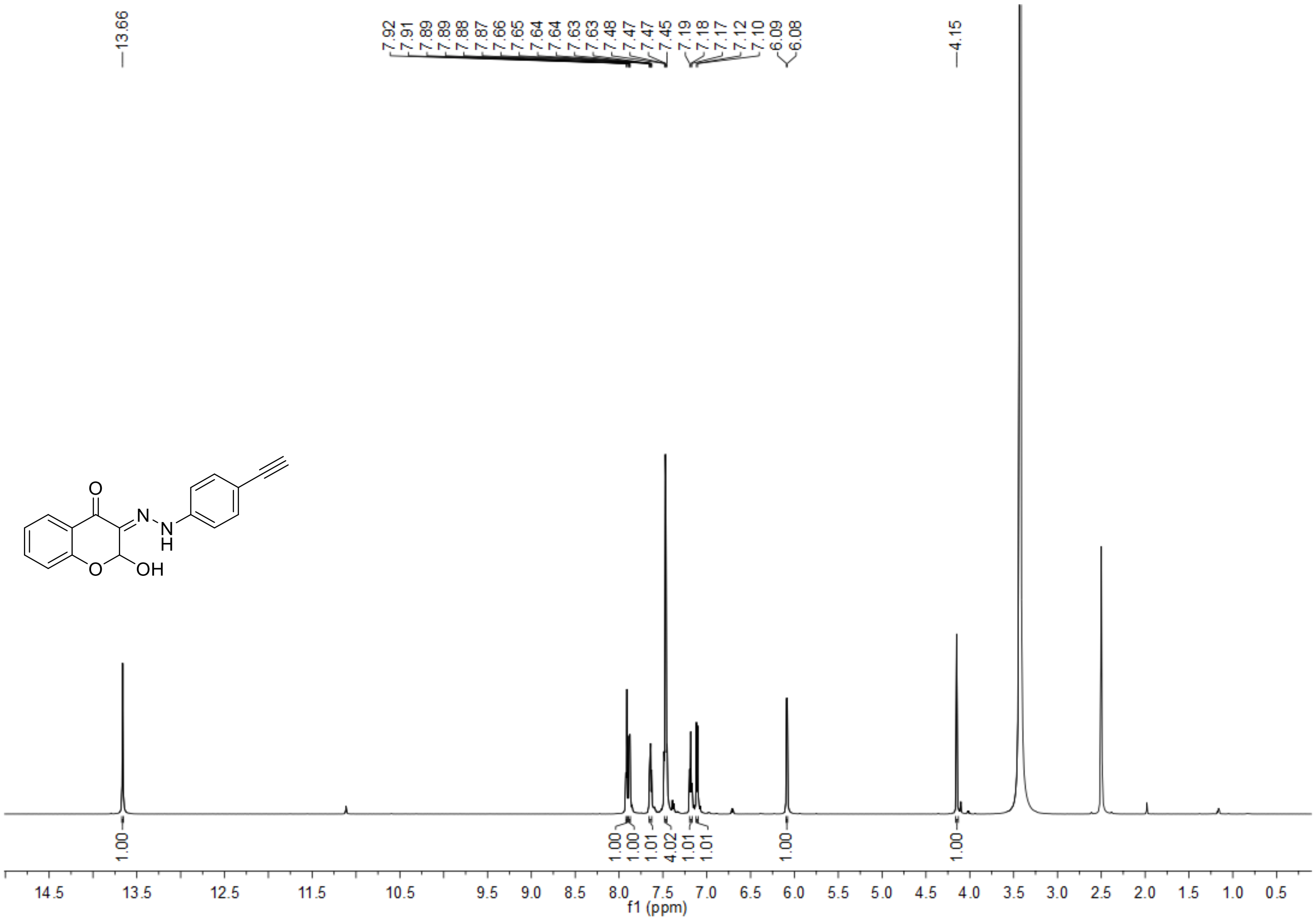

Figure S46. ^{1}H NMR (600 MHz, DMSO-d_6) spectra of compound **3v**

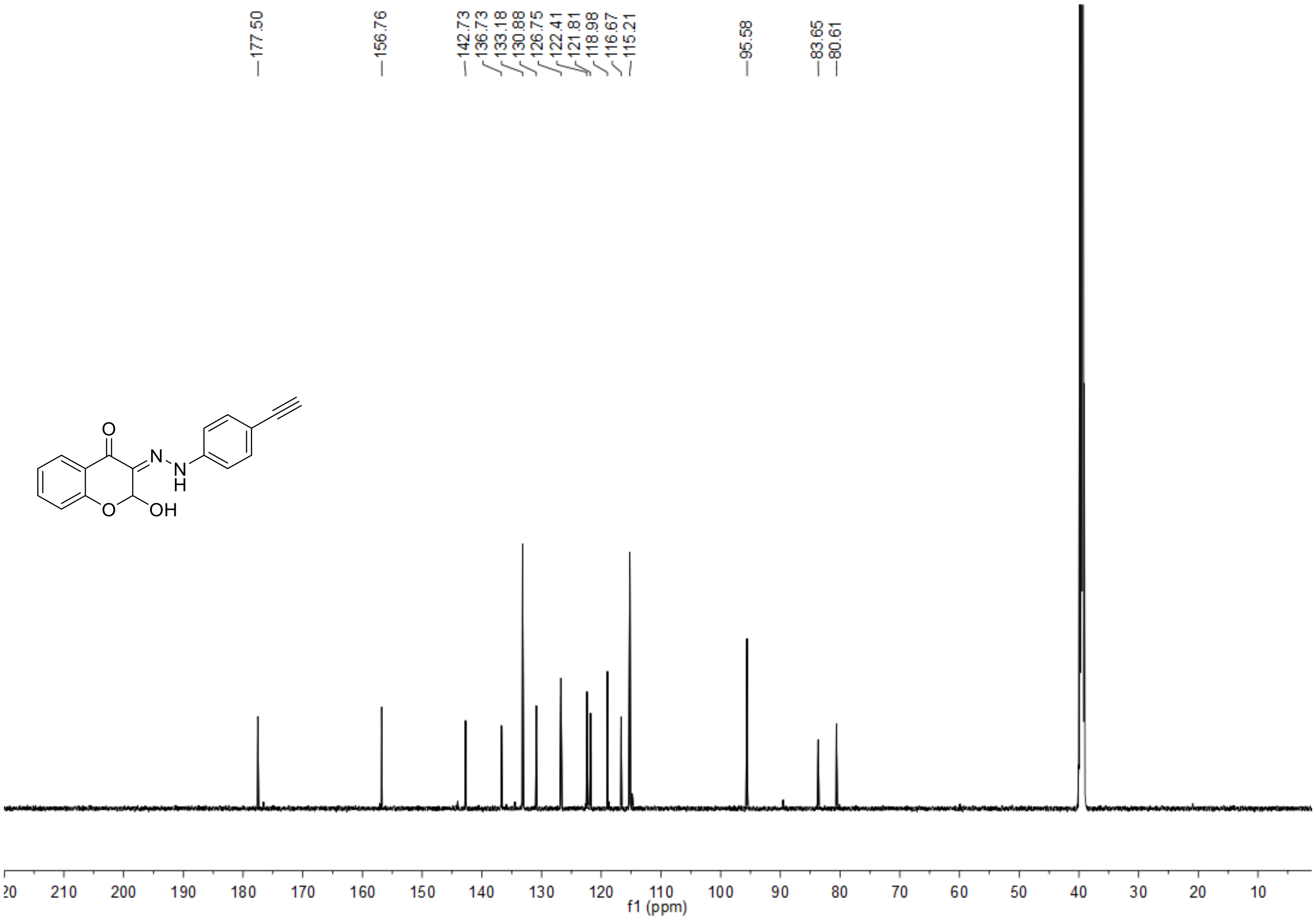

Figure S47. ^{13}C NMR (150 MHz, DMSO-d_6) spectra of compound **3v**

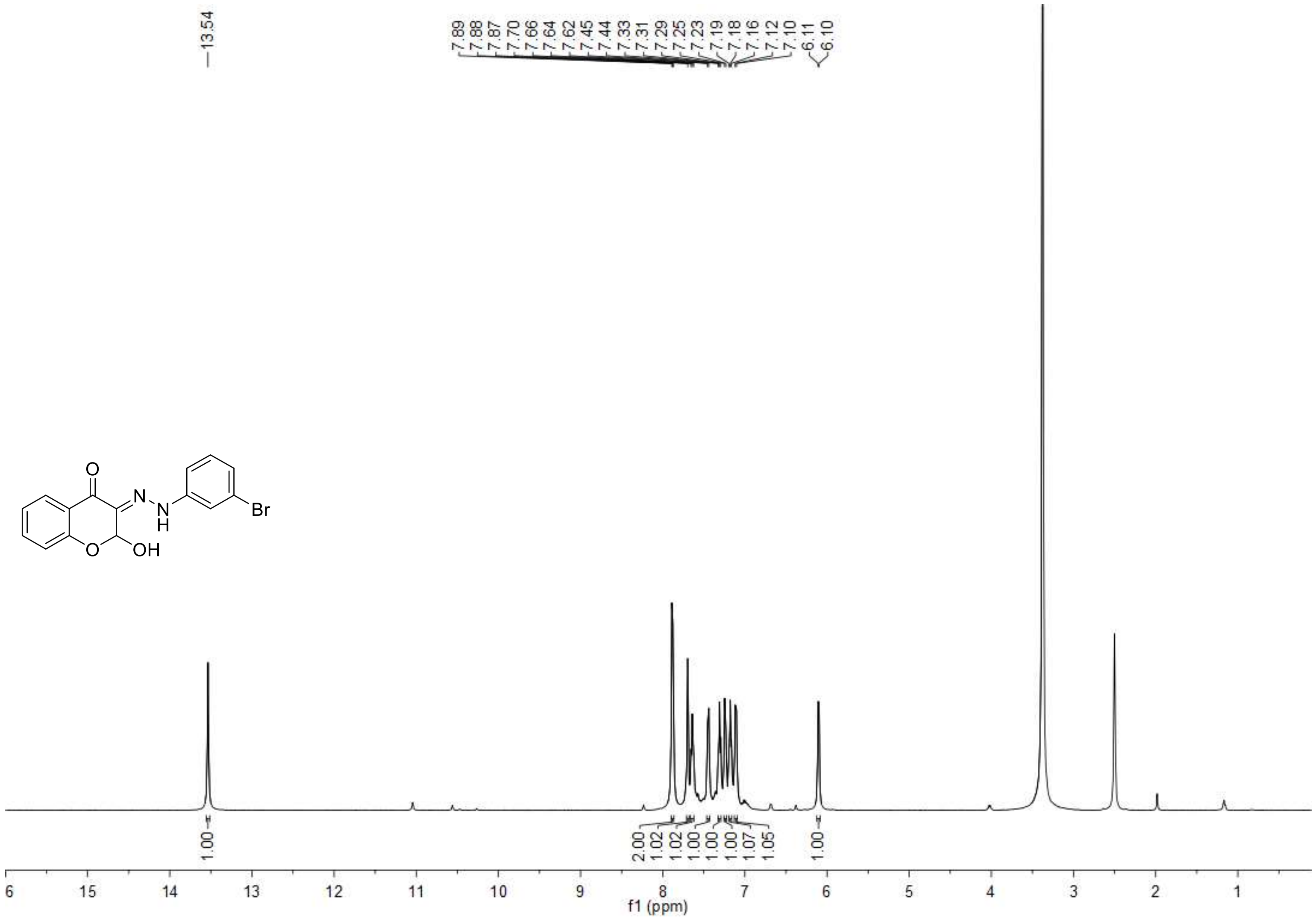

Figure S48. ^{1}H NMR (500 MHz, DMSO-d_6) spectra of compound **3w**

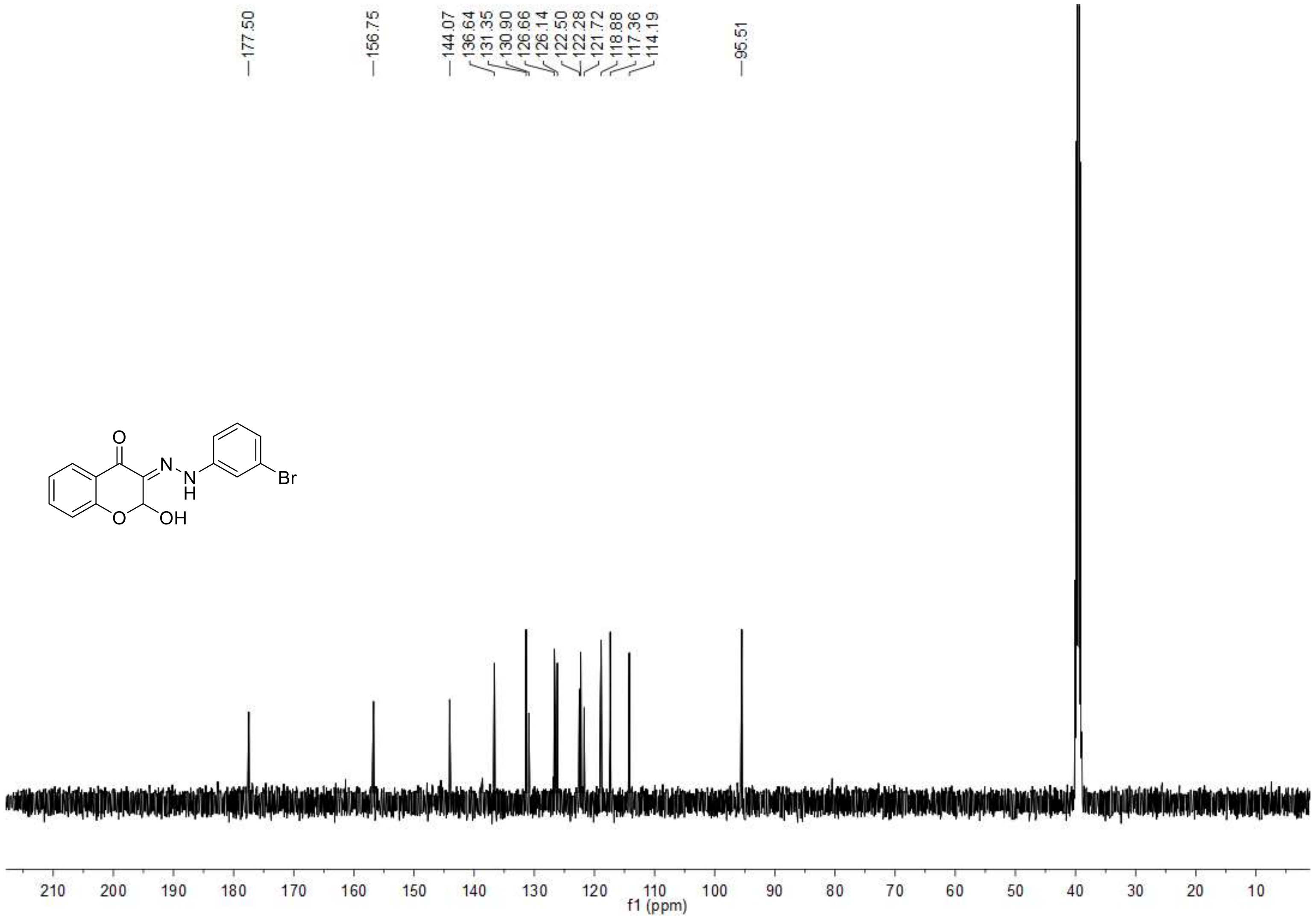

Figure S49. ^{13}C NMR (125 MHz, DMSO-d_6) spectra of compound **3w**

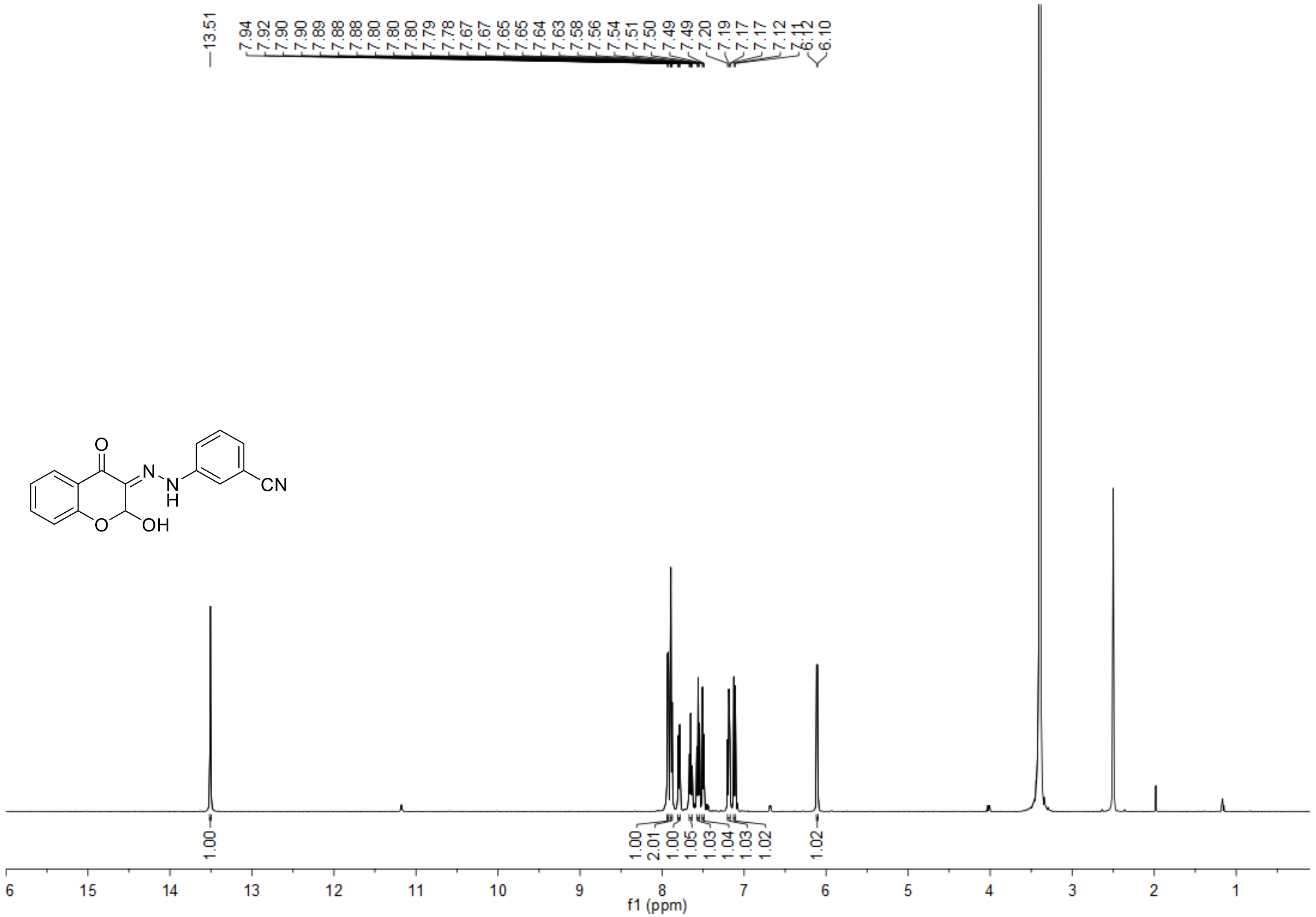

Figure S50. ^{1}H NMR (500 MHz, DMSO-d_6) spectra of compound **3x**

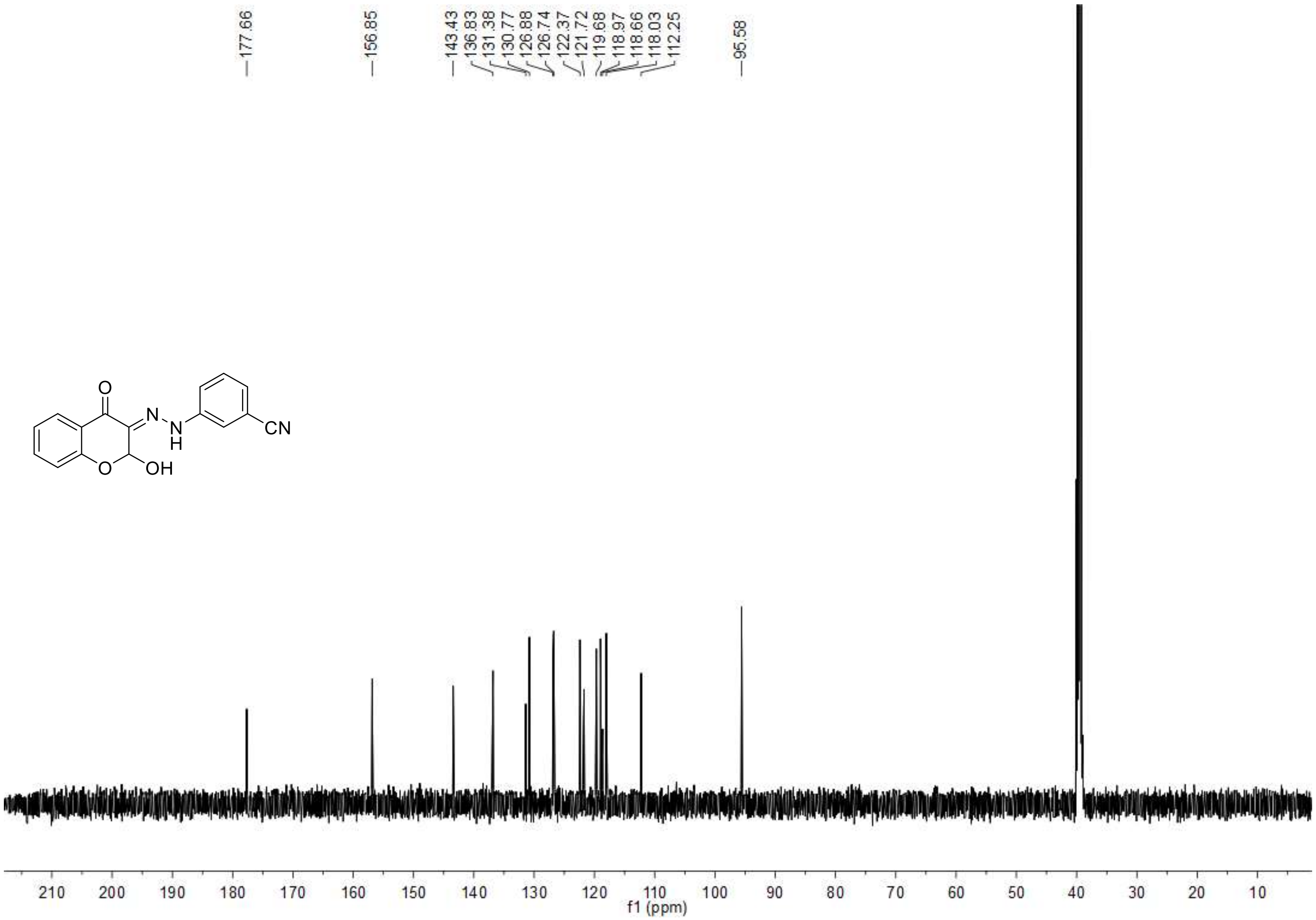

Figure S51. ^{13}C NMR (125 MHz, DMSO-d_6) spectra of compound **3x**

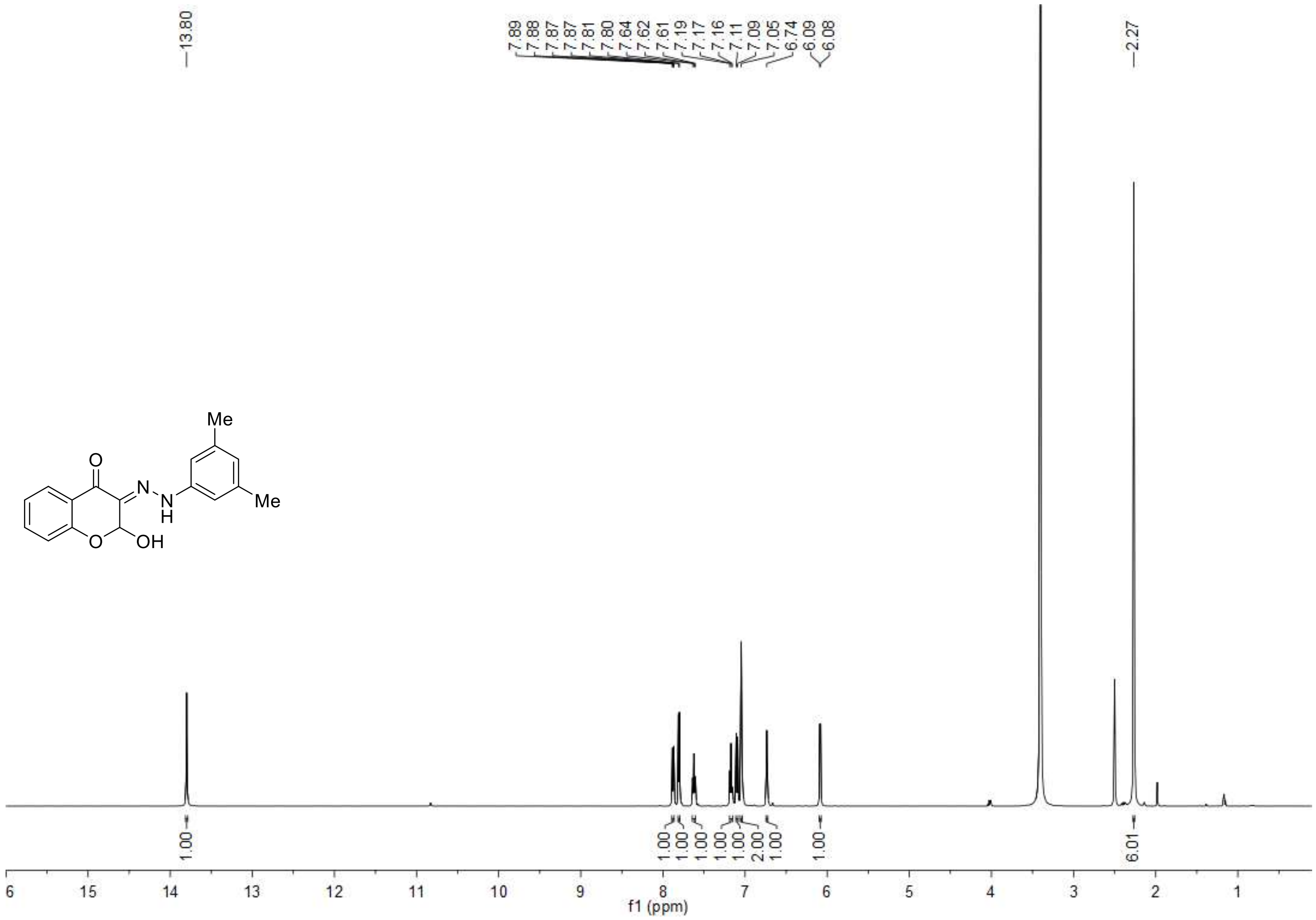

Figure S52. ¹H NMR (500 MHz, DMSO-d_6) spectra of compound **3y**

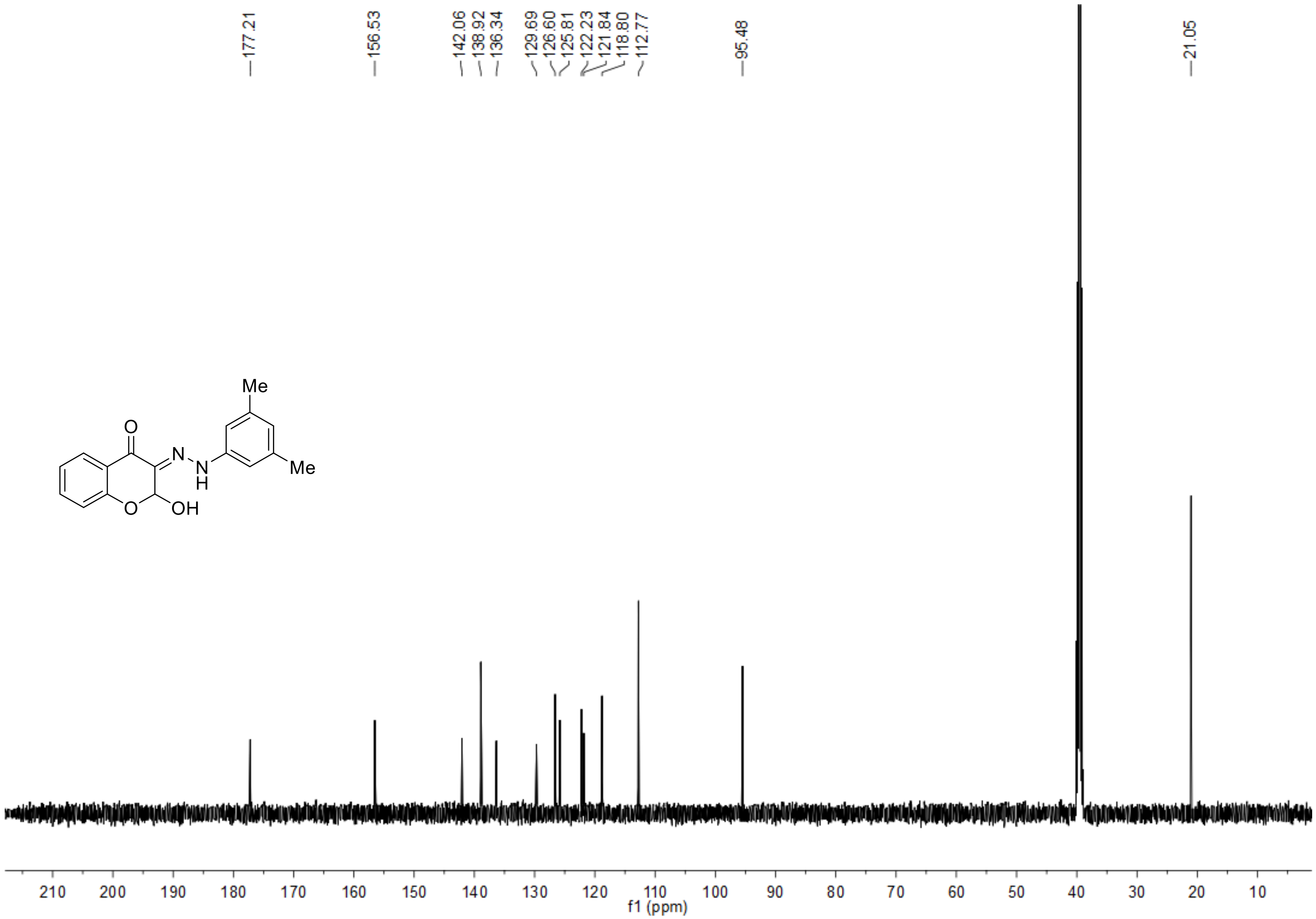

Figure S53. ^{13}C NMR (125 MHz, DMSO-d_6) spectra of compound **3y**

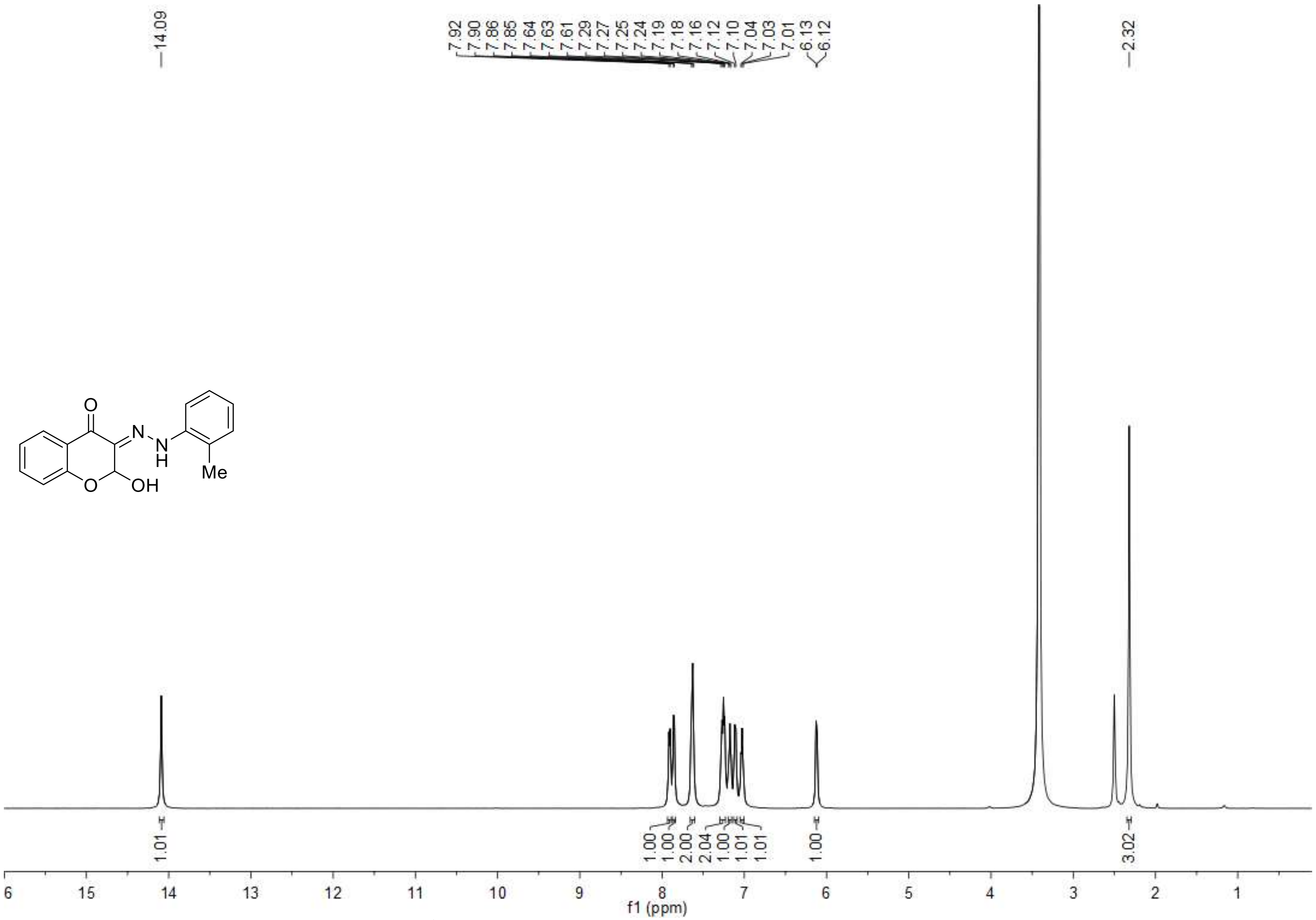

Figure S54. ^{1}H NMR (500 MHz, DMSO-d_6) spectra of compound **3z**

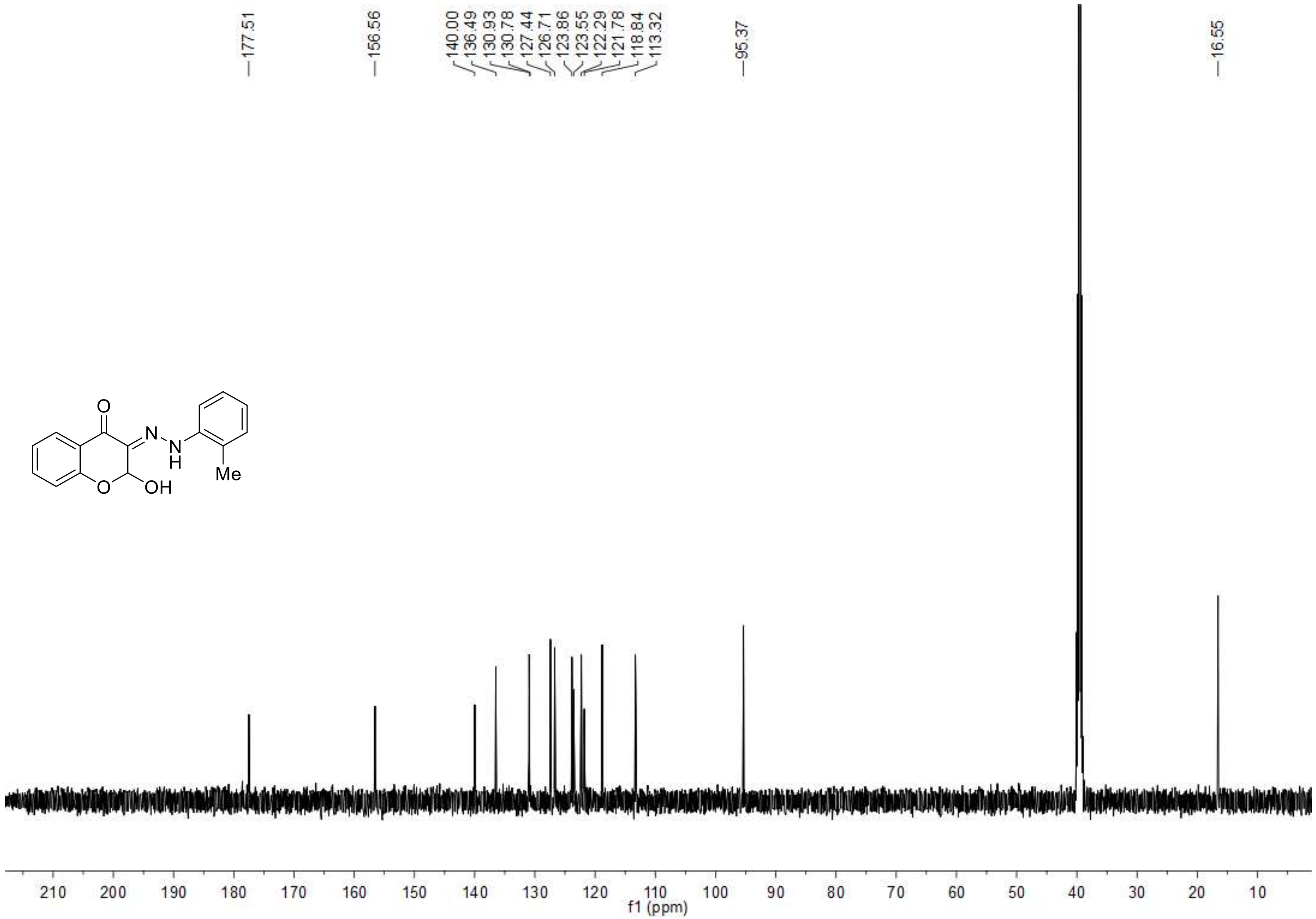

Figure S55. ^{13}C NMR (125 MHz, DMSO-d_6) spectra of compound **3z**

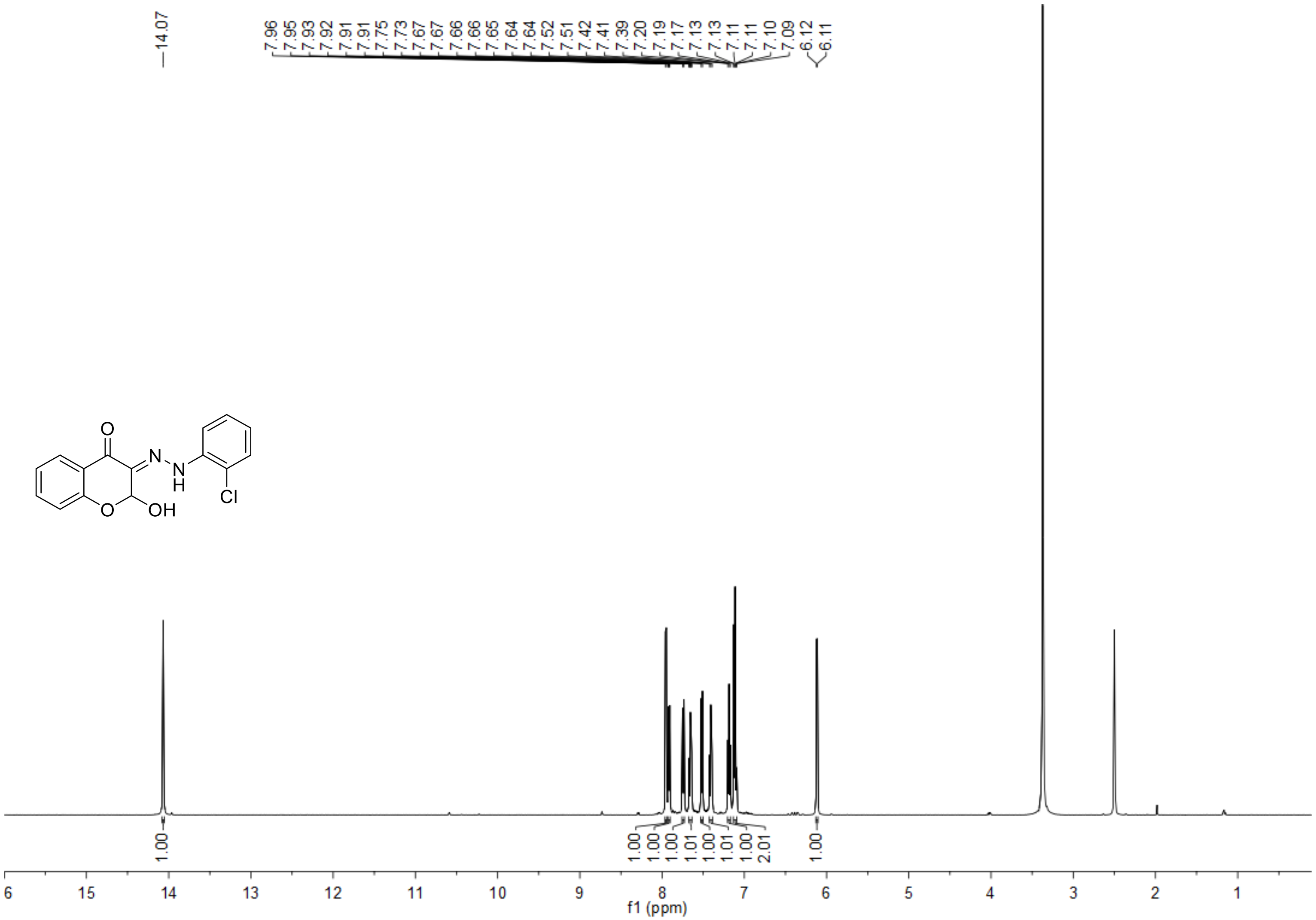

Figure S56. ^{1}H NMR (500 MHz, DMSO-d_6) spectra of compound **3a'**

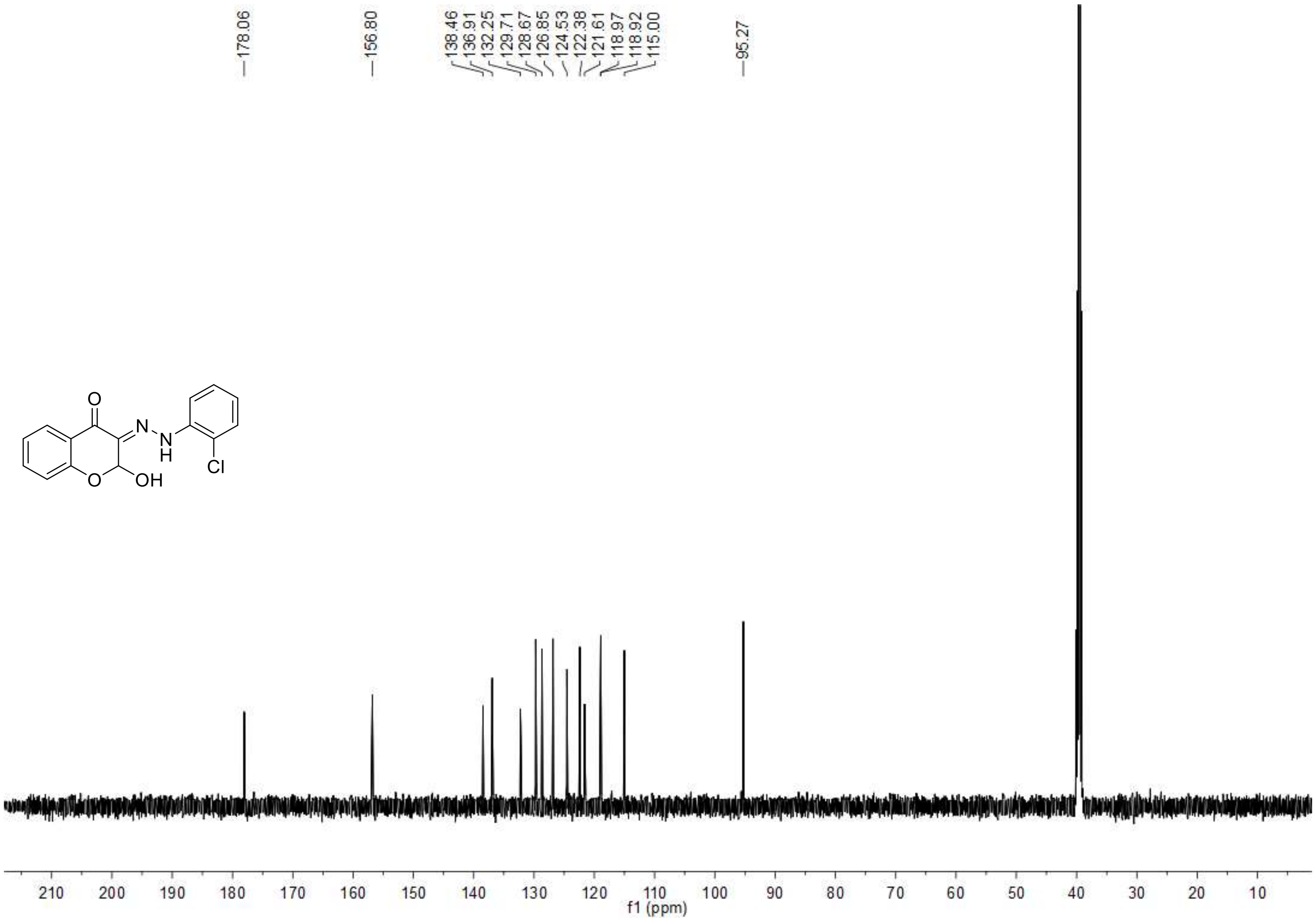

Figure S57. ^{13}C NMR (125 MHz, DMSO-d_6) spectra of compound **3a'**

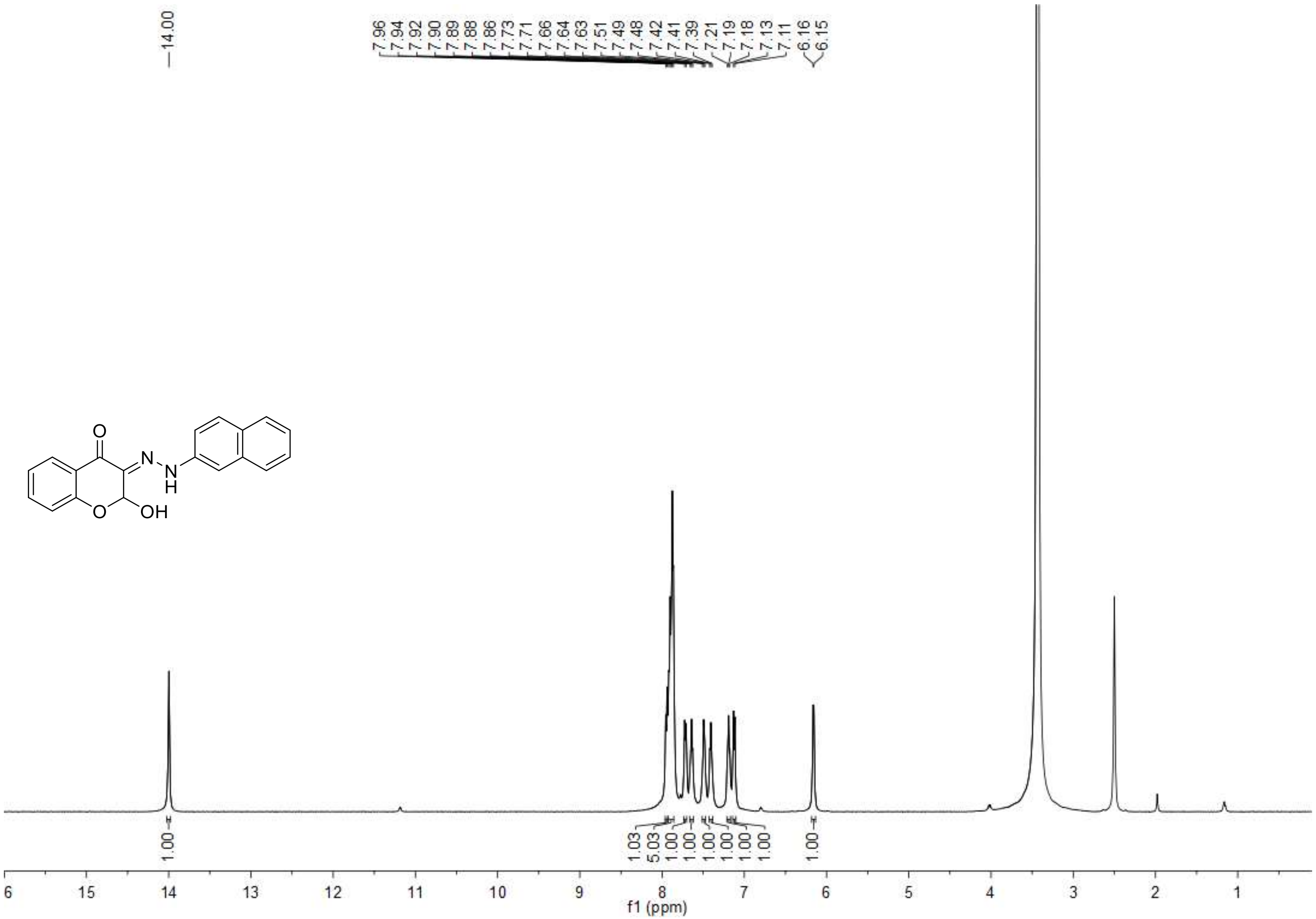

Figure S58. ^{1}H NMR (500 MHz, DMSO-d_6) spectra of compound **3b’**

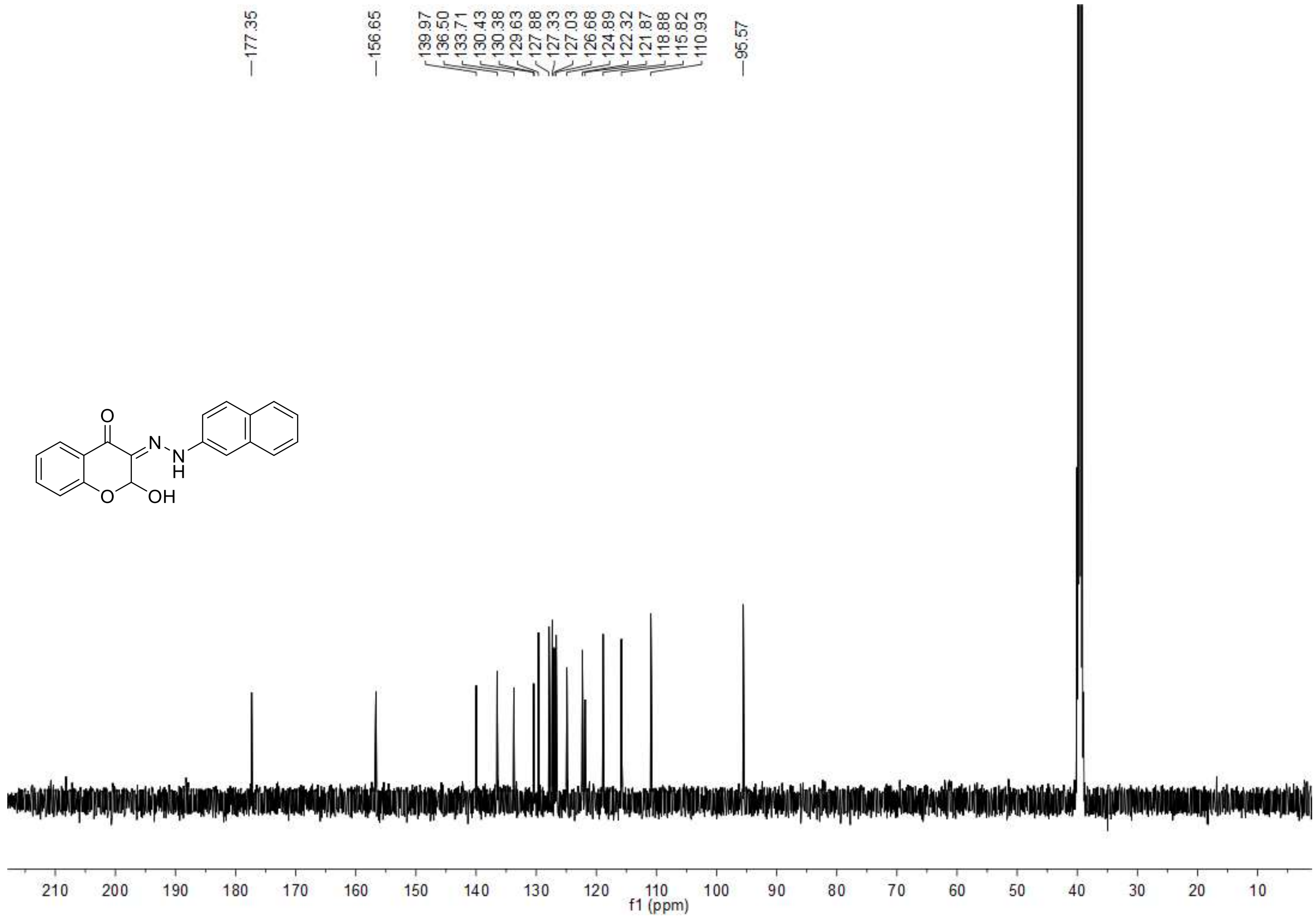

Figure S59. ^{13}C NMR (125 MHz, DMSO-d_6) spectra of compound **3b’**

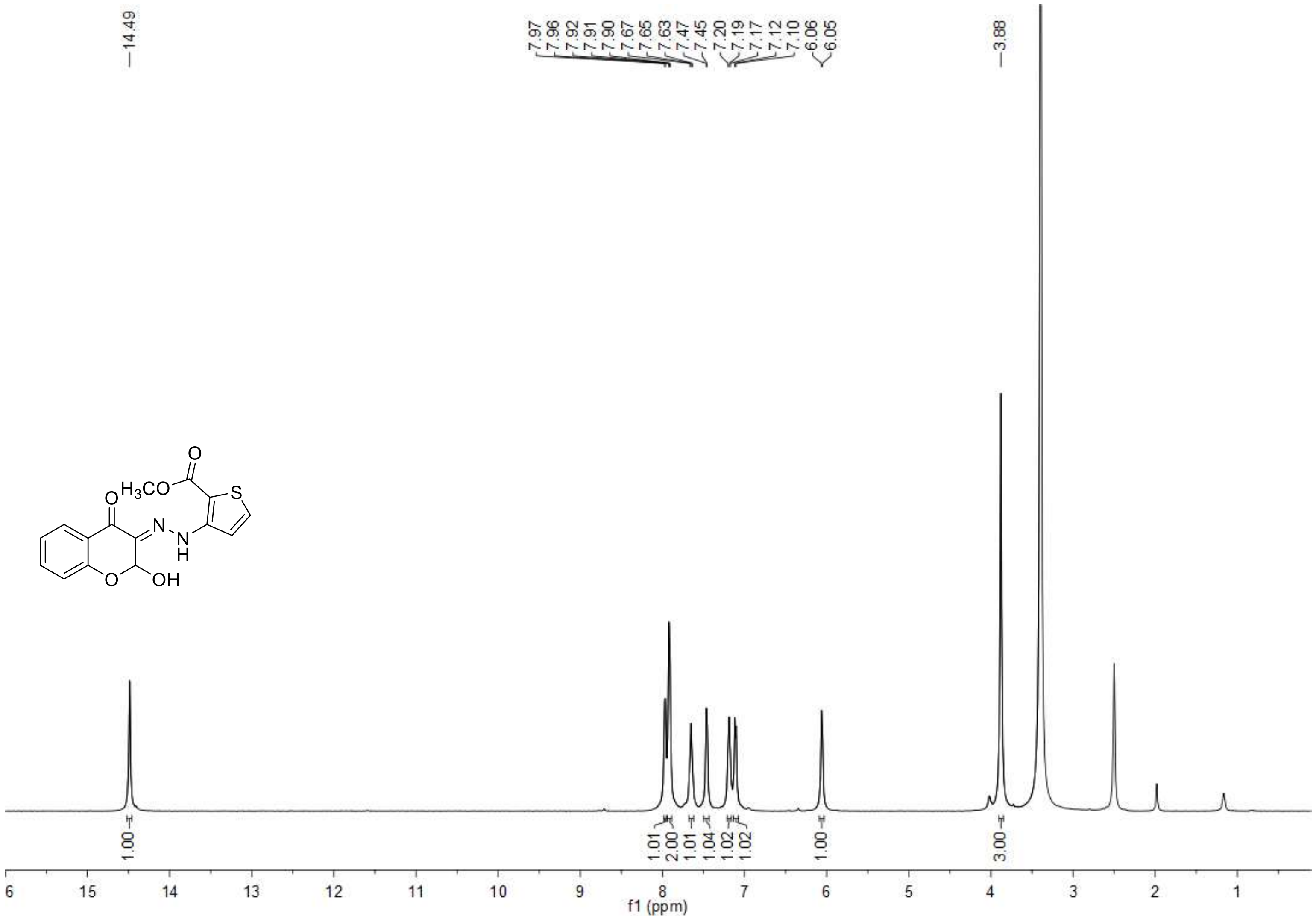

Figure S60. ^{1}H NMR (500 MHz, DMSO-d_6) spectra of compound **3c'**

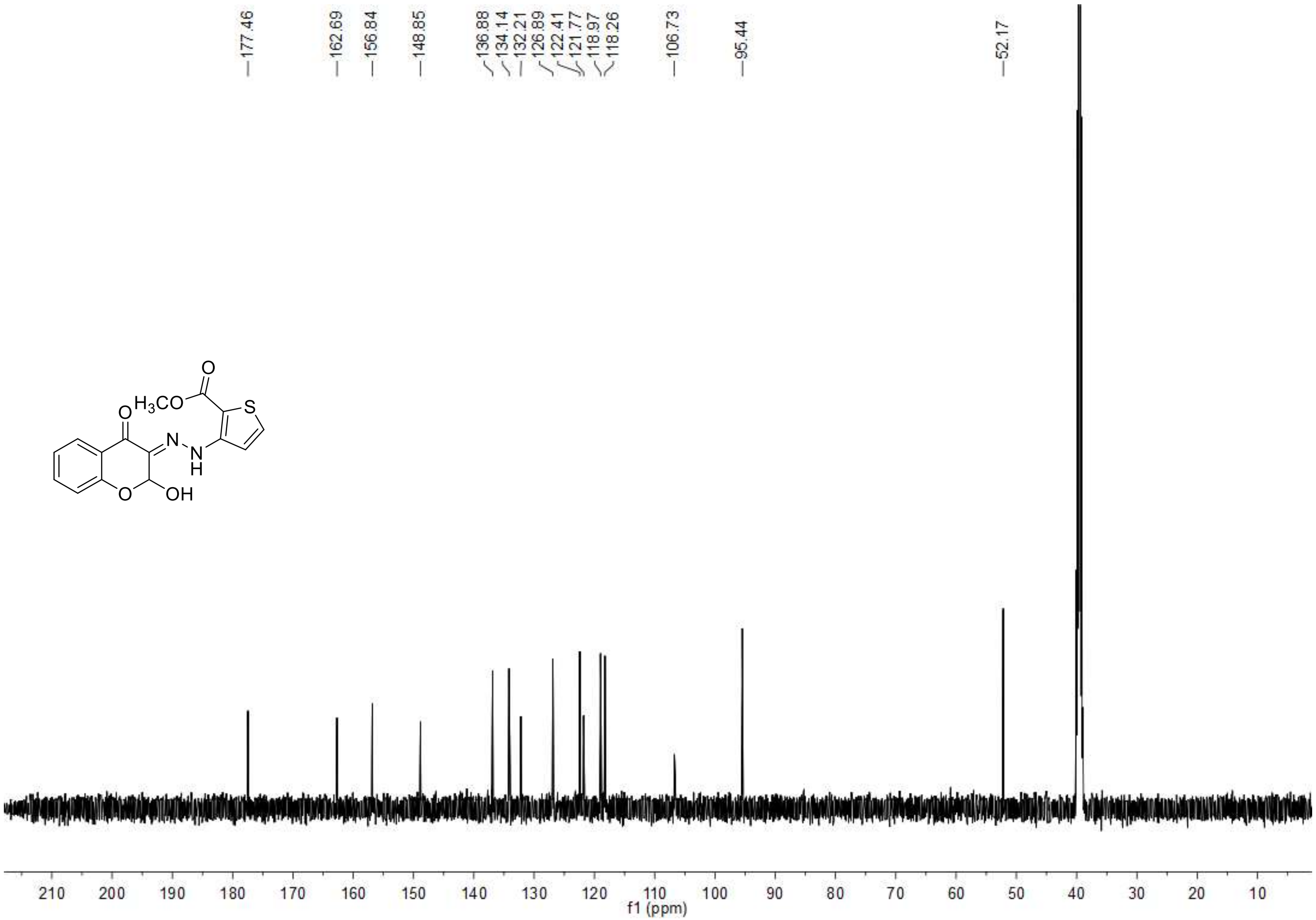

Figure S61. ^{13}C NMR (125 MHz, DMSO-d_6) spectra of compound **3c’**

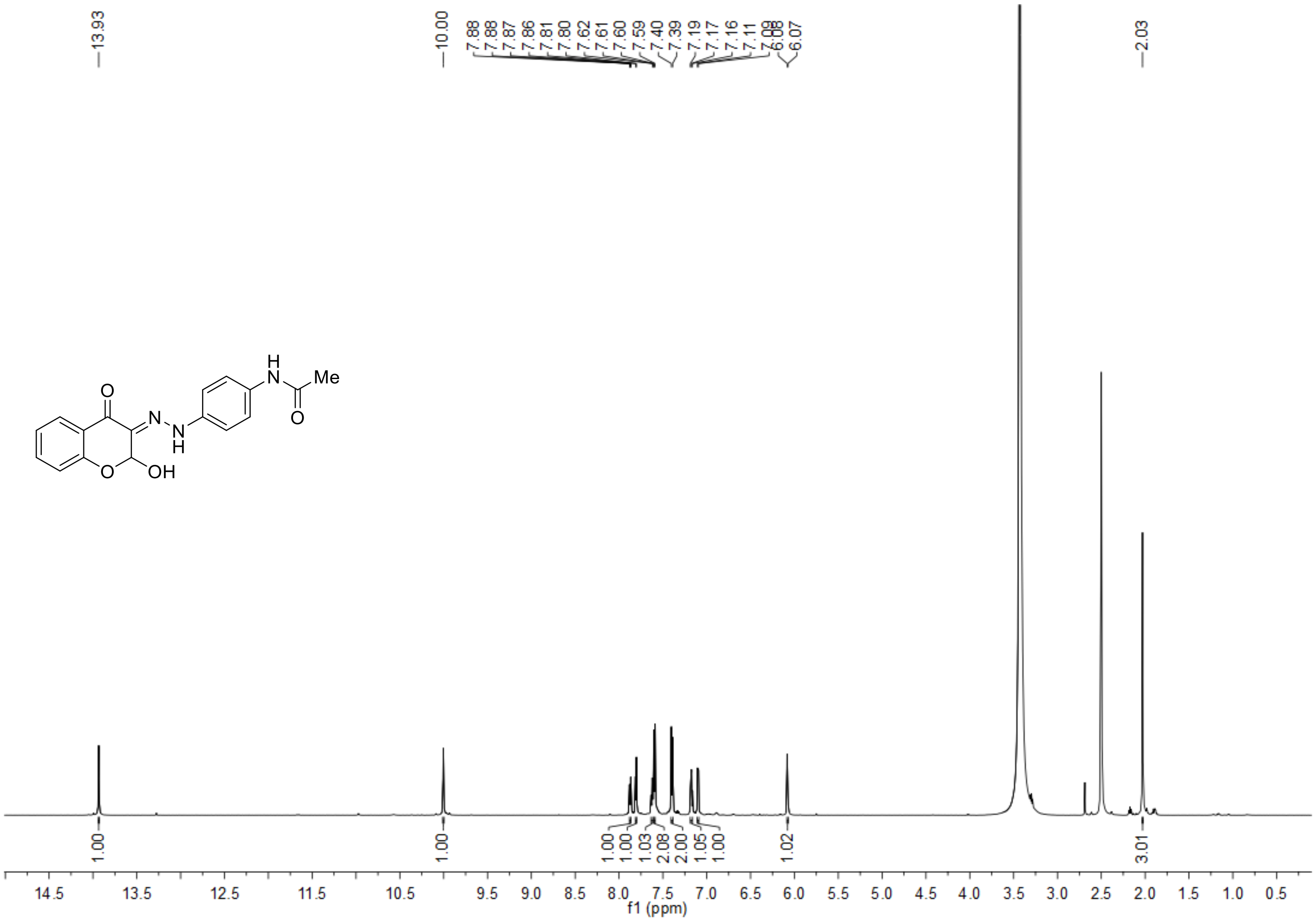

Figure S62. ^{1}H NMR (600 MHz, DMSO-d_6) spectra of compound **3d'**

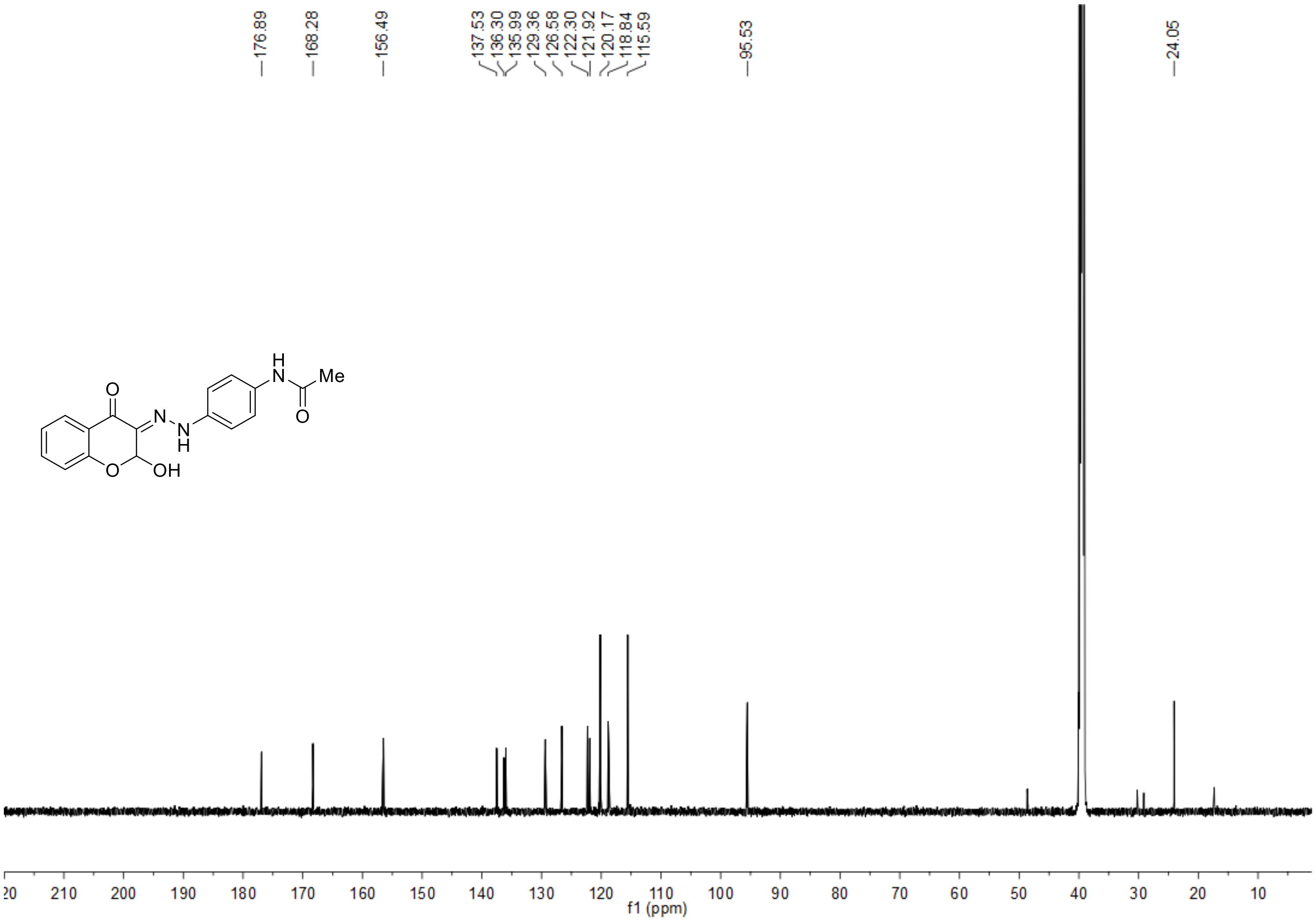

Figure S63. ^{13}C NMR (150 MHz, DMSO-d_6) spectra of compound **3d’**

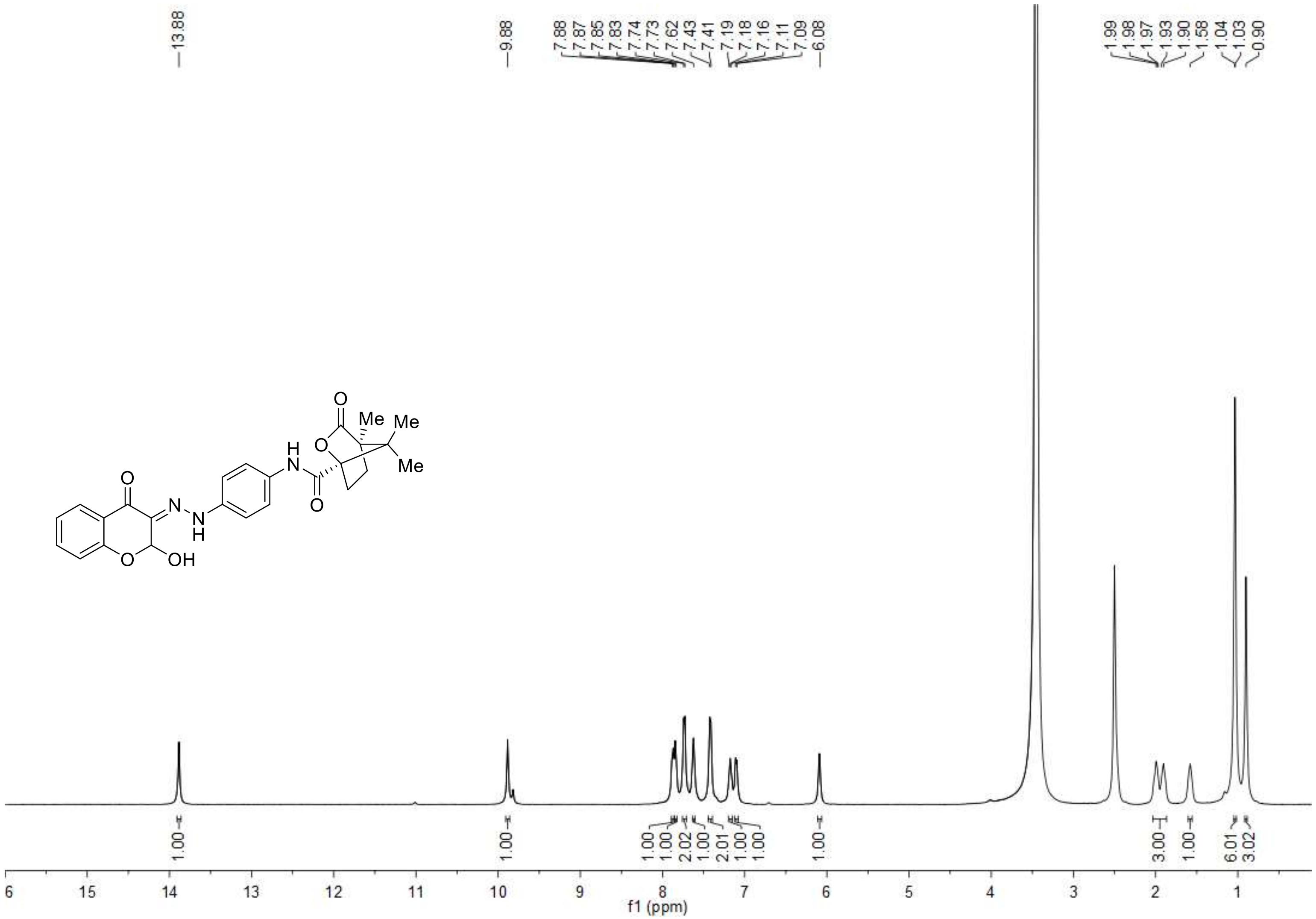

Figure S64. ^{1}H NMR (500 MHz, DMSO-d_6) spectra of compound **3e’**

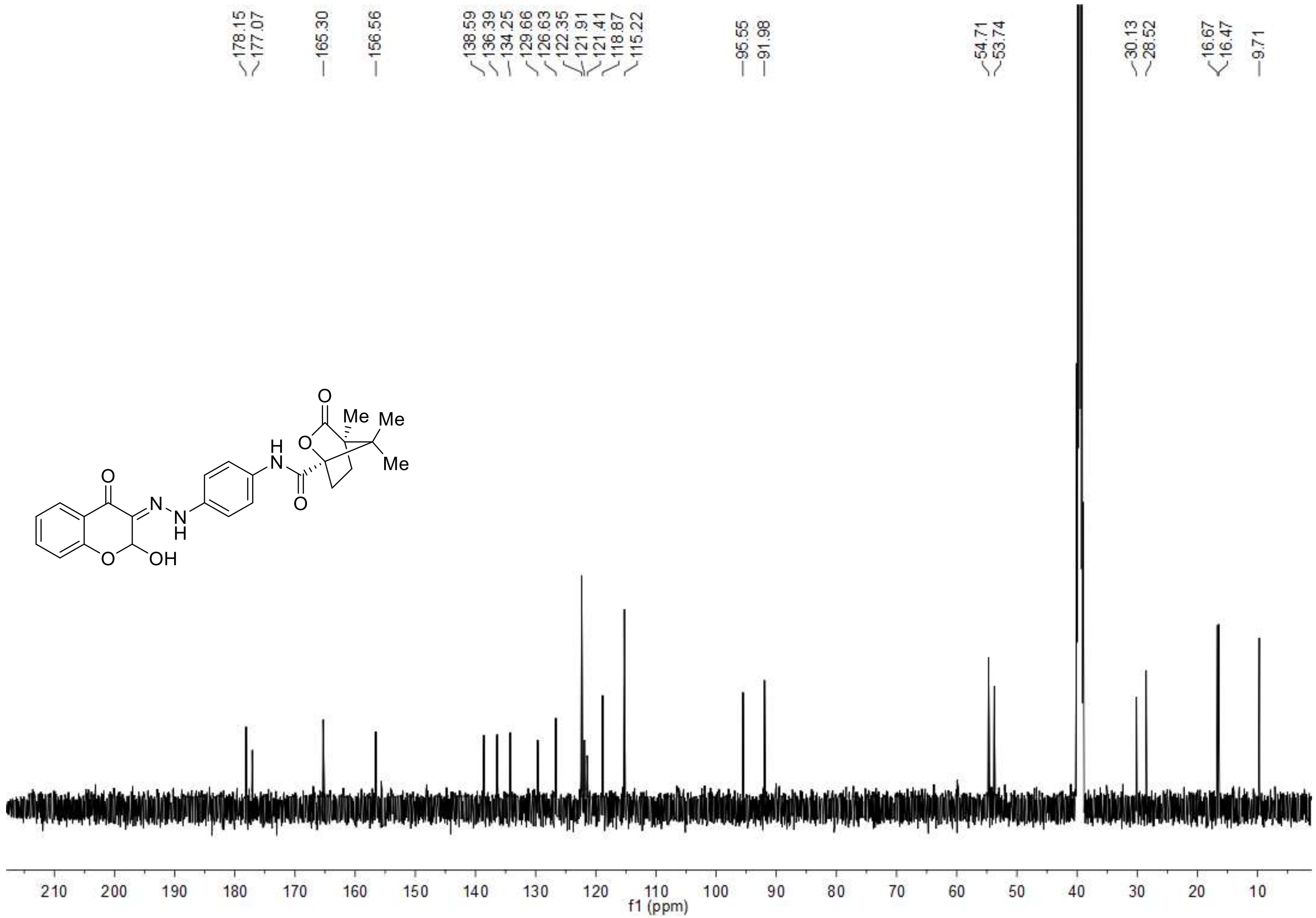

Figure S65. ^{13}C NMR (125 MHz, DMSO-d_6) spectra of compound **3e'**

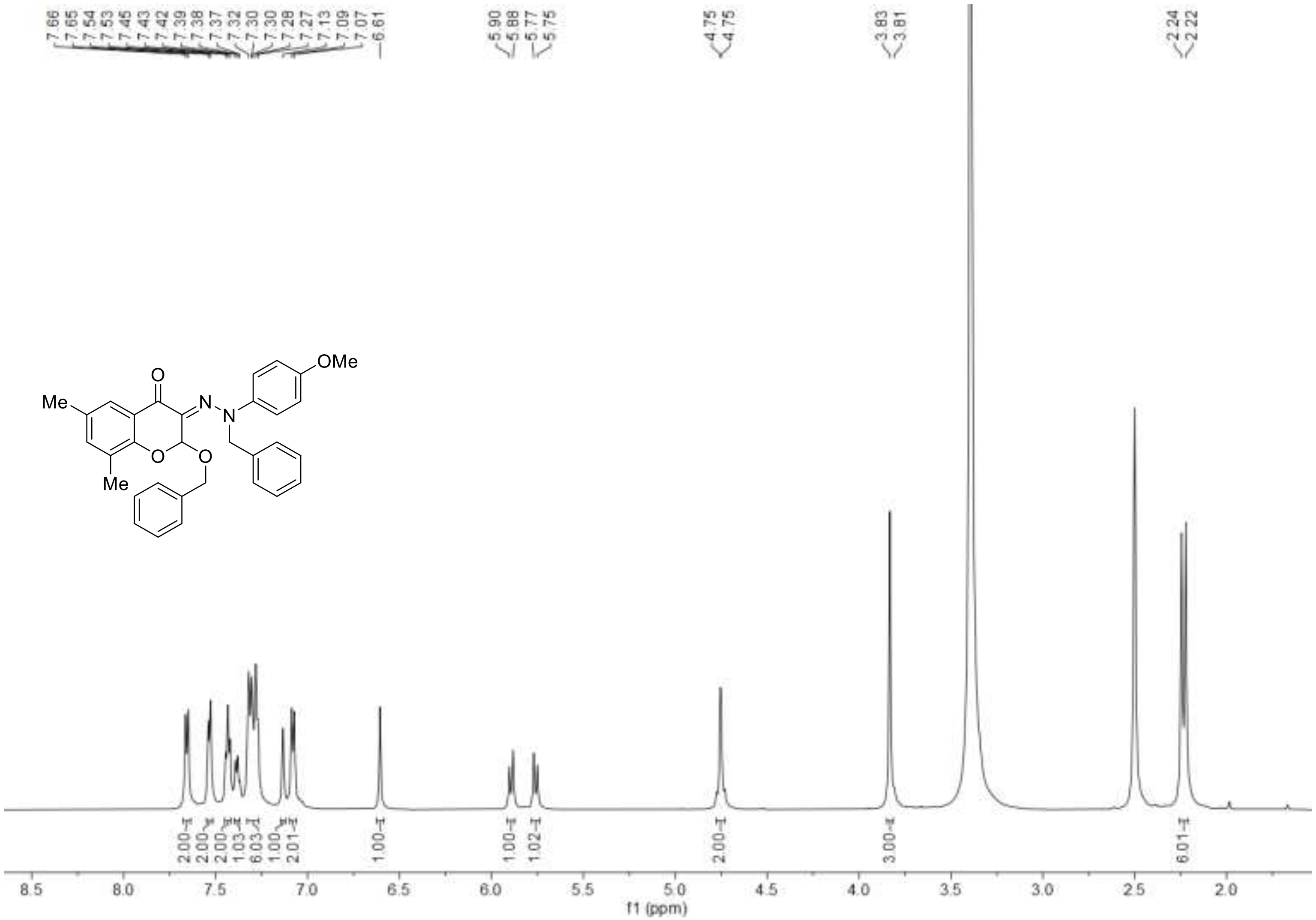

Figure S66. ^{1}H NMR (600 MHz, DMSO-d_6) spectra of compound **4**

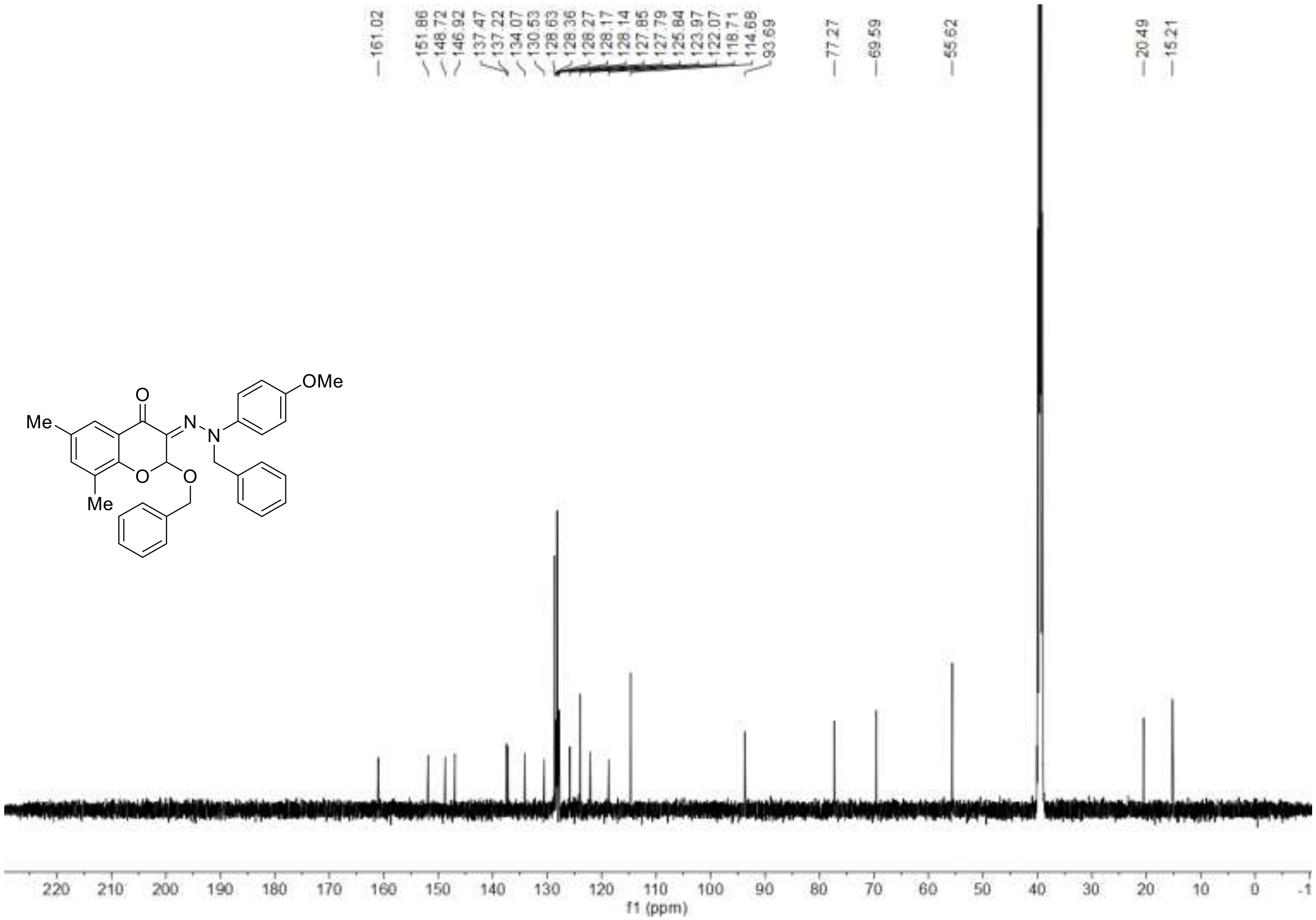

Figure S67. ^{13}C NMR (150 MHz, DMSO-d_6) spectra of compound **4**

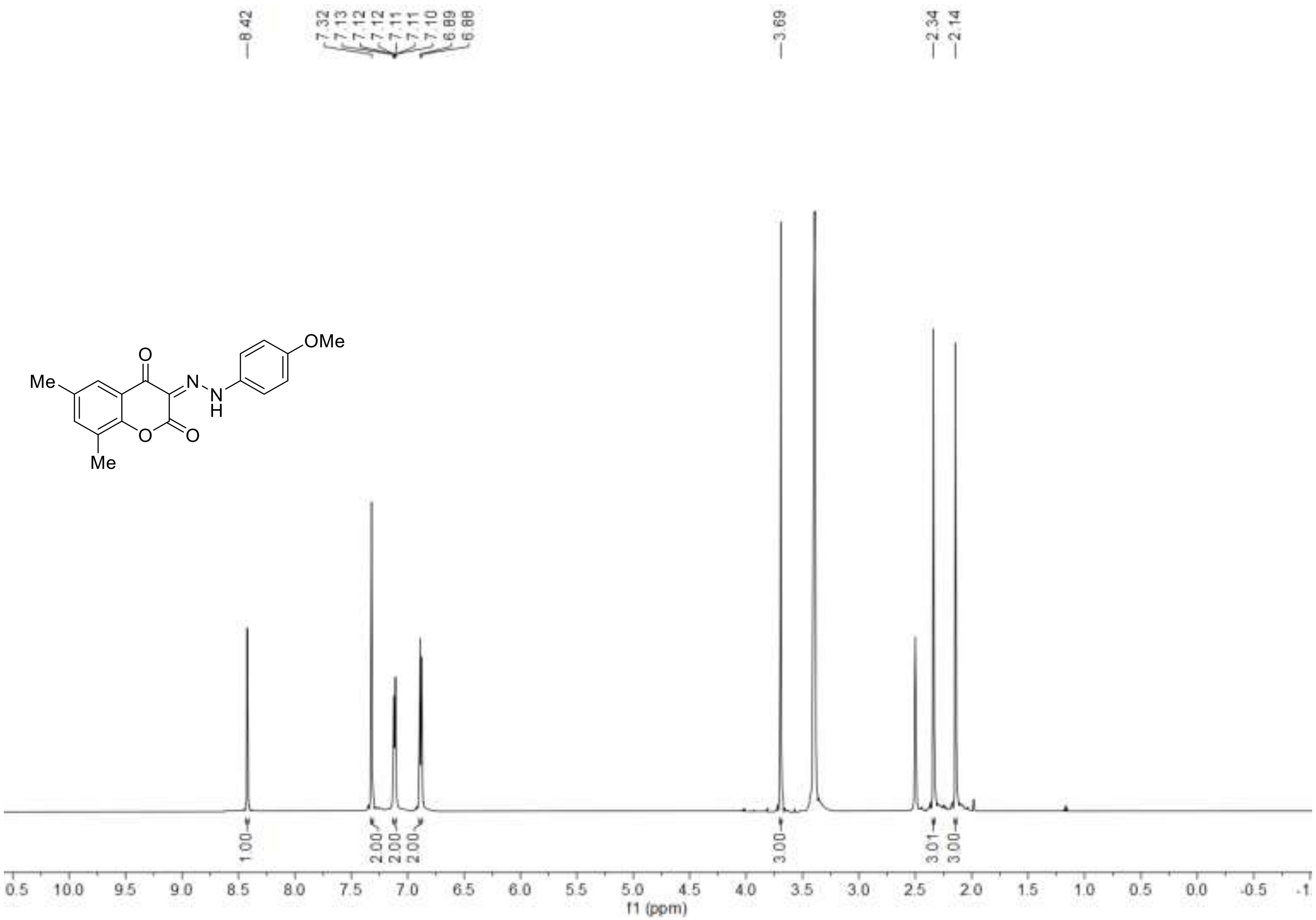

Figure S68. ^{1}H NMR (600 MHz, DMSO-d_6) spectra of compound **5**

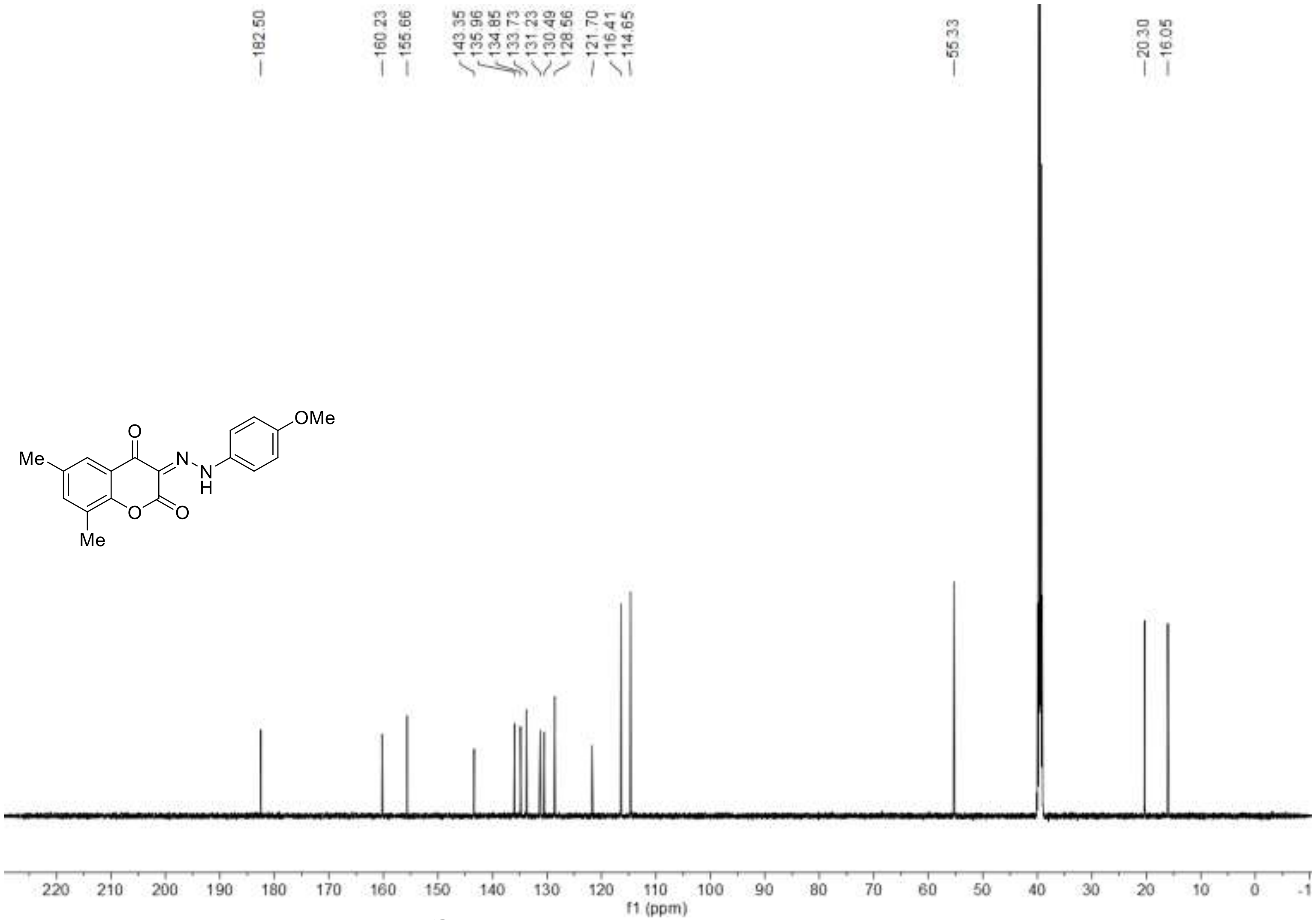

Figure S69. ^{13}C NMR (150 MHz, DMSO-d_6) spectra of compound **5**
